# Hydrogenation of Quinolines and Aldehydes Catalyzed by a Pyrolyzed, Augmented Cobalt-Salen Complex

**DOI:** 10.1021/acsomega.5c04940

**Published:** 2025-08-06

**Authors:** Fabian Schmiedbauer, Uwe Monkowius, Clemens Schwarzinger, Stefan Müllegger, Stephan Bartling, Nils Rockstroh, Christoph Topf

**Affiliations:** † Institute of Inorganic Chemistry, 27266 Johannes Kepler University (JKU), Linz 4040, Austria; ‡ Linz School of Education, Johannes Kepler University (JKU), Linz 4040, Austria; § Institute for Chemical Technology of Organic Materials (CTO), Johannes Kepler University (JKU), Linz 4040, Austria; ∥ Institute of Semiconductor and Solid State Physics, Solid State Physics Division Johannes Kepler University (JKU), Linz 4040, Austria; ⊥ Leibniz Institute for Catalysis at the University of Rostock (LIKAT), Rostock 18059, Germany; # Institute of Catalysis (INCA), Johannes Kepler University (JKU), Linz 4040, Austria

## Abstract

We report on the preparation and characterization of a robust and easy-to-handle composite catalyst based on an enlarged salcomine-type solution-phase precursor. Wet impregnation of silica with an extended, homobimetallic cobalt-salen complex followed by controlled pyrolysis of the imbued and dried support afforded the fully functional catalytically active material. The solid catalyst thus produced affected the heterogeneous hydrogenation of quinolines and aldehydes, which selectively gave 1,2,3,4-tetrahydroquinolines and primary alcohols, respectively. In order to showcase its versatility, our catalyst was tested under batch conditions in steel autoclaves as well as in a continuous flow reaction system, which produces the requisite hydrogen gas *in situ* through water electrolysis (H-Cube Mini Plus from ThalesNano). In both operating modes, we observed very good product yields and decent functional group tolerance.

## Graphical abstract

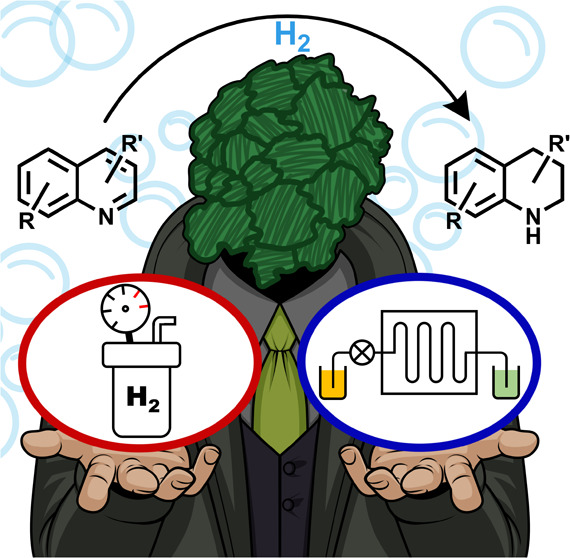

## Introduction

The catalytic reduction of quinolines with H_2_ gas to yield 1,2,3,4-tetrahydroquinolines (THQs) is an important chemical transformation since it provides atom-efficient access to key building blocks that are used for the manufacture of indispensable pharmaceuticals.^1^ Classic homogeneous hydrogenation protocols rest on the use of precious metals from Group 8 (Ru, Os) or 9 (Rh, Ir)^2^, whereat enantioselective versions are, for example, enabled by Pd complexes.^3^ Yet, scarcity issues linked to the ongoing deployment of noble elements have spurred research directed toward the utilization of more abundant metals, including Fe,^4^ Co,^5^ Mn,^6,7^ and Mo.^8,9^ Recently, we have shown that a W-based piano stool complex also promotes the quinoline-to-THQ hydrogenation.^10^ Besides gaseous hydrogen, proper surrogates such as Hantzsch ester,^11^ oxazaborolidine borane,^12^ or ammonia borane^13,14^ can also function as the principal reductants.

With respect to heterogeneous approaches, catalysts made from coinage metals,^15,16^ Group 9,^17,18^ and Group 10 elements^19–21^ are suitable for the hydrogenation of *N*-heteroarenes. Noteworthy, we previously found that cobalt particles that were generated in situ from simple and cheap salts and zinc powder effected the same transformation, even in water.^22^ Again, certain transfer hydrogenation reagents, e.g., formic acid,^23,24^ ammonia borane,^25,26^ and tetrahydroxydiboron^27^ have been described to function as substitutes for H_2_ in a variety of heterogeneously catalyzed quinoline reductions.

Among the many heterogeneous formulations that facilitate (transfer) hydrogenation reactions, those prepared through pyrolyses of supported metal complexes^28^ command a privileged position. This is mainly due to the fact that the properties of the full-fledged catalysts are easily controlled at the stage of a molecularly well-defined precursor. Variation of the ligand scaffold,^29^ metal cation(s), and carrier material allows for great flexibility in setting the catalytic capabilities. Regarding cobalt as principal active metal, combinations with carbon,^30–33^ silica,^34,35^ alumina,^36^ hydroxyapatite,^37^ and ZIF-67 zeolite^38^ were especially effective in the syntheses of a variety of *N*-heterocycles including quinolines. Strikingly, even a merged, forest-derived Co catalyst prepared from pine needles was shown to effect the hydrogenation of various (hetero)­arenes.^39^

Concerning potent precursor compounds, metal-salen complexes are attractive candidates as they are readily crafted into redox active catalysts either by way of inclusion into zeolites^40–43^ or through pyrolytic activation.^44^ Moreover, the diimine ligand platform, which usually coordinates to d-block transition metals, can be amended with oxygen-rich donor atoms that allow for the inclusion of additional Lewis acidic f-block elements.^45–47^ This feature opens up many steering possibilities and allows for the design of heterobimetallic catalysts that operate under very mild reaction conditions. Now, we introduce a further applicability of a vanillin-derived double Schiff base which we earlier used for the fabrication of a NiCe and a monometallic Ni catalyst that were applied to the reduction of nitroarenes^48^ and reductive amination of ketones,^49^ respectively. Herein, we delineate the (de)­hydrogenation potential of a congeneric Co catalyst that was tested in batch mode as well as under continuous flow conditions. Especially the latter is an attractive alternative when safety issues are raised associated with the use of flammable and explosive H_2_ gas. The flow reactor used generates the needed, gaseous H_2_ in situ from water electrolysis and thus dispenses with the need to handle bulky and heavy H_2_ storage bottles. Furthermore, flow hydrogenation processes display excellent automation capacities.^50,51^

## Results and Discussion

### Synthesis of the Dinuclear Precursor Complex

Imine condensation between *ortho*-vanillin **A** (2 equiv) and diamine **B** (1 equiv) furnished the double Schiff base ligand **H**_**2**_ **L** (Scheme 1) which, in addition to the classic diimine donor motif, incorporates four O-based ligands that enable the inclusion of a second metal cation which is usually derived from an f-block element.^48^ Further reaction of **H**_**2**_ **L** with Co­(OAc)_2_·4 H_2_O (1 equiv) exclusively gave, to our surprise, the homobimetallic complex **[Co**_**2**_ **L]** and not the related mononuclear coordination compound similar to the one which was previously prepared by us from the same ligand and Ni­(OAc)_2_·4 H_2_O.^49^ The preferred formation of dinuclear **[Co**_**2**_ **L]** is in stark contrast to prior reports on ligands that only contain the standard N_2_O_2_ donor pattern that naturally ligate only one Co cation.^52,53^ The results of EPR spectroscopy suggest that **[Co**_**2**_ **L]** is a mixed-valence complex that contains both Co­(II) and Co­(III) species whereby the softer Co­(II) coordinates to the diimine moiety, whereas the harder Co­(III) is embedded into the oxygen environment. Furthermore, single crystal X-ray diffraction analysis revealed that both cobalt centers are bridged by two acetate ions. A comprehensive summary of the EPR and X-ray measurements on **[Co**_**2**_ **L]** is given in the Supporting Information.

**Scheme 1:**
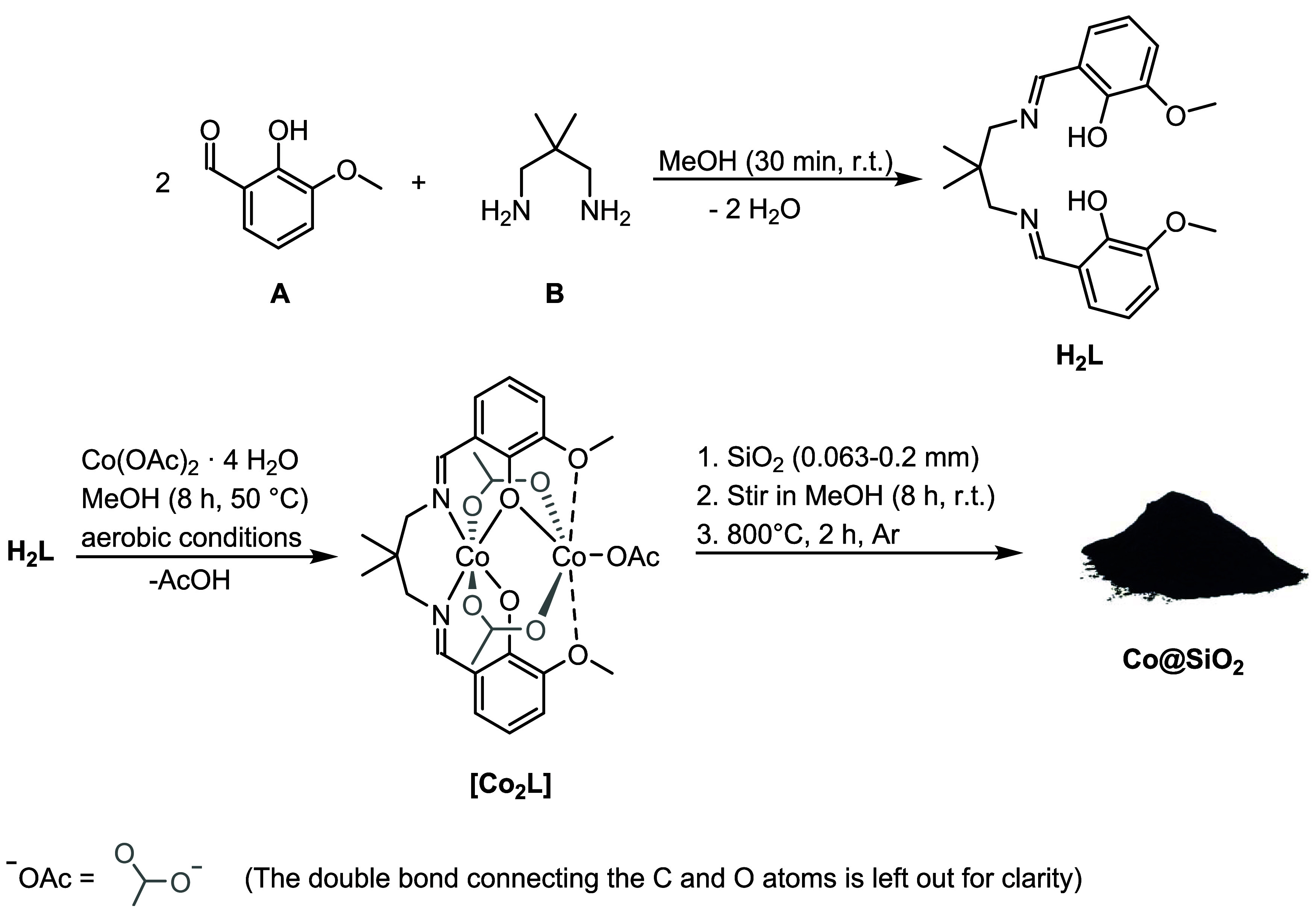
Preparation of the Extended Salen Ligand H_2_L, the Solution-Phase Precursor [Co_2_L], and the Full-Fledged Solid Catalyst **Co@SiO_2_**

### Catalyst Preparation and Characterization

First, finely dispersed silica (0.063–0.200 mm) was impregnated with a solution of precursor complex **[Co**_**2**_ **L]** in methanol. The imbued support was then vacuum-dried (3 days) and thereafter pyrolyzed under controlled conditions in a protective argon atmosphere (800 °C, 2 h). The resultant black solid composite material **Co@SiO**_**2**_ (Scheme 1) was pestled and then ready to use. Noteworthy, any extra activation steps so as to obtain a full-fledged, catalytically active material were not necessary. The bulk elemental analysis showed that the catalyst contained 2.71% Co, 3.97% C, 0.10% H, and 0.18% N. For the sake of comparison, we probed further inorganic oxides and carbon black for their suitability to function as proper carrier materials for pyrolytically activated **[Co**_**2**_ **L]** (vide infra), where the corresponding catalysts were all prepared according to the above-mentioned conditions.

The surface of the catalyst **Co** **@SiO**_**2**_ was investigated by X-ray photoelectron spectroscopy (XPS). The near-surface region is composed of Si and O as the main components (see survey spectrum in SI, Figure S5). To a minor extent C (7.3 atom %), Co (0.3 atom %), and N (0.2 atom %) can be found (see spectra in SI Figure S-XPS1–4). The Co 2p spectrum is shown in Figure 1 and fitted with a multiplet structure together with satellite peaks.^54^ The main peaks are around 780.6 and 795.8 eV, which can be assigned to Co 2p_3/2_ and Co 2p_1/2_ in the oxidic state. The rather small satellite features around 787 and 803 eV suggest the presence of Co_3_O_4_ as the main oxidation state at the surface of the Co particles.^54^ However, the coexistence of Co^2+^ to a minor extent cannot be excluded completely. The observed Co 2p binding energies are about 0.6 eV higher compared to literature values for Co_3_O_4_, which might be caused by the interaction with the support.^54^

**Figure 1:**
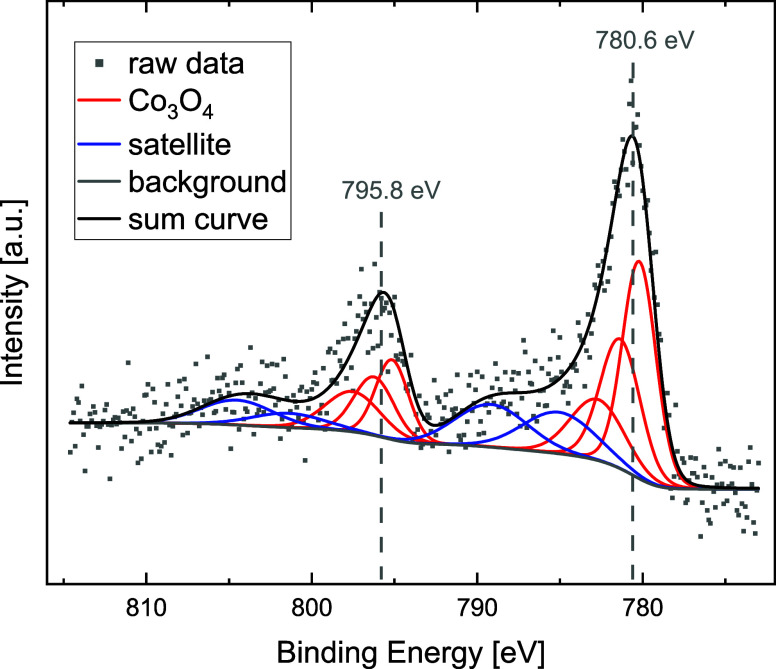
XPS analysis of the Co 2p region of solid **Co@SiO_2_**.

Scanning Transmission Electron Microscopy (STEM) of **Co@SiO_2_** was used to observe the structure of the catalyst. It could be shown that Co-containing nanoparticles are present mainly in sizes between 2 and 8 nm that are not uniformly distributed on the support (Figure 2a–c). However, parts of the precursors transformed to a carbonaceous support-like species, which features cobalt-containing nanoparticles with sizes of up to 60 nm (Figure 2d–f). It should be noted that most of the Co is present in metallic form (Figure 2a,d), which is a consequence of the reaction conditions (800 °C, oxygen-deficient atmosphere, i.e., under Ar) and was also observed for similarly prepared materials with Fe and Co.^28,55^ In the case of large Co nanoparticles, however, considerable amounts of oxidized cobalt form a shell around the metallic core, which has also been observed by contrast (Figure 2e,f). This surface oxidation is in good agreement with XPS results and might originate from the noninert transfer of the materials to the respective machines. The presence of Co as a constituent of the respective nanoparticles was proven by energy-dispersive X-ray spectroscopy (Figure 3).

**Figure 2:**
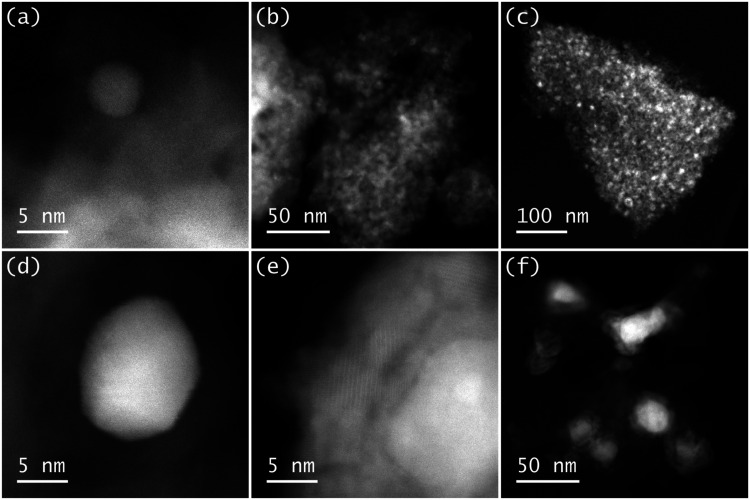
(a–f). Selected HAADF-STEM images were of **Co@SiO**_**2**_.

**Figure 3:**
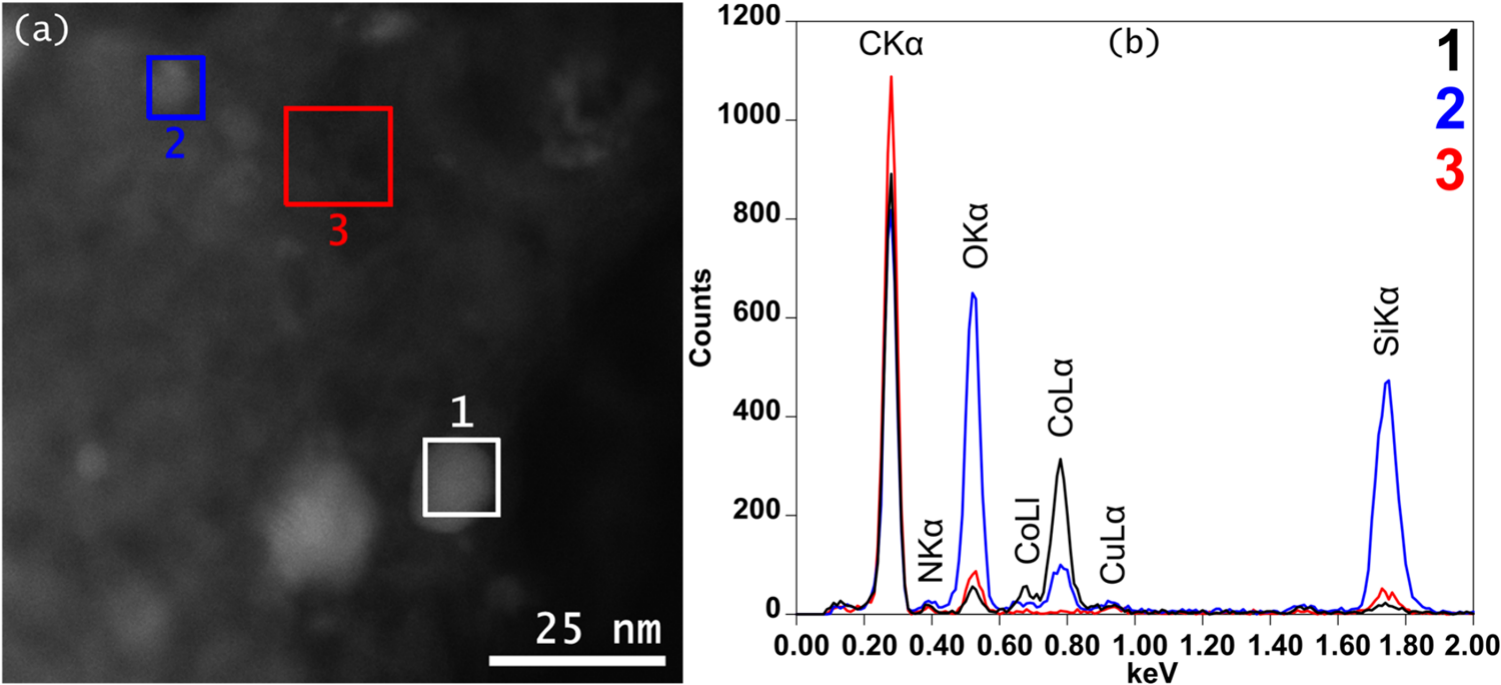
Selected EDX spectra (b) of the highlighted regions in the respective HAADF-STEM image (a) of **Co@SiO_2_**. Cobalt is clearly visible in areas 1 and 2, while it is absent in area 3. The oxygen signal is correlating with the presence of Si, and thus, Co is very likely in its metallic state (see spectrum 1).

### Initial Catalytic Tests

First, we investigated different potential supports for the **[Co**_**2**_ **L]** precursor and assessed the performance of the related catalysts in the batch hydrogenation of quinoline **1a** with the goal of selectively synthesizing 1,2,3,4-tetrahydroquinoline **2a** (Table 1). Since previous reports communicated the aptitude of Vulcan,^56^ CeO_2_,^57^ MgO,^58^ SiO_2_,^49^ and Al_2_O_3_ ^36^ for the fabrication of solid hydrogenation catalysts, we opted for these materials in this study, too. Given the polymorphism and the associated amorphous character of alumina,^59^ we also tested different modifications of this oxide (Supporting Information). We found that Vulcan, CeO_2_, and MgO gave only poor results that were not at all capable of improvement (Entries 1–3). Hence, we focused on SiO_2_ and α-Al_2_O_3_ during the catalyst optimization process. Although the first yields of **2a** obtained with the silica- and alumina-supported catalysts were also quite modest (entries 4 and 5), we were able to devise efficient hydrogenation protocols on simply increasing the catalyst loading (vide infra).

**Table 1:**
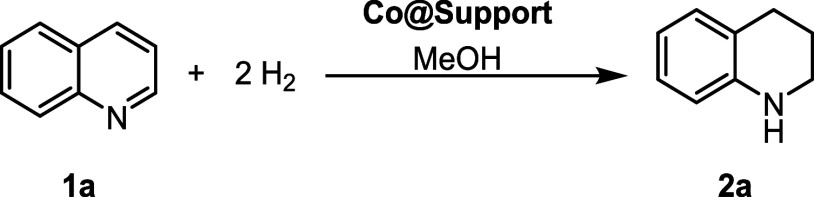
Influence of the Support on the Co-Catalyzed Quinoline Hydrogenation^a^

entry	support	yield **2a**/%
1	Vulcan	8
2	CeO_2_ (5 μm)	n.d.
3	MgO (<50 nm)	4
4	SiO_2_ (0.063 – 0.200 mm)	12
5	activated α-Al_2_O_3_ (<1 μm)	42

a Reaction conditions (batch): **1a** (0.25 mmol), MeOH (2 mL), **Co@support** (15 mg), 80 °C, H_2_ (50 bar), and 16 h reaction time. The reported particle sizes of the support materials are given in parentheses. The yield of **2a** was determined through GCMS analysis, whereby *n*-hexadecane served as internal standard; n.d. means “not detected”.

In another test series we explored the influence of the reaction medium on the performance of **Co@Al**_**2**_ **O**_**3**_ and **Co@SiO**_**2**_ (Table 2). The former gave excellent results in H_2_O and MeOH but interestingly, when similar protic solvents were utilized (EtOH, *i*-PrOH), we observed a steep decline in product yield (Entries 1–4). The use of ethers (Et_2_O, THF, and 1,4-dioxane) as well as aprotic nonpolar hydrocarbons (toluene, *n*-heptane) gave rise to unsatisfactory outcomes and the yields of **2a** ranged from 11 to 30%. Strongly coordinating (pyridine, ACN) and chlorinated solvents (DCM, CHCl_3_) also proved to be unsuitable for the given quinoline hydrogenation (1–21% yield). To our surprise, we established that the silica-supported congener **Co@SiO**_**2**_ is only applicable in MeOH. Inspection of Table 2 reveals that there is no real alternative to this C1 alcohol; even structurally related EtOH caused the activity of this catalyst to cease almost completely (entries 2 and 3).

**Table 2:**
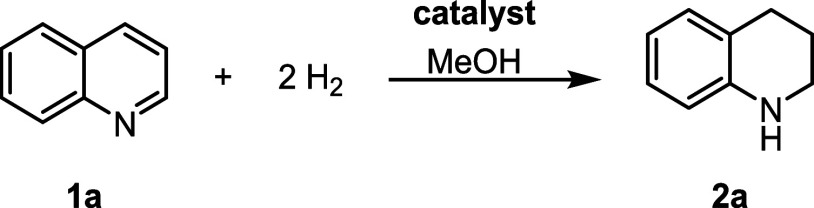
Solvent Variation Study for Alumina- and Silica-Supported Catalysts

entry	solvent	**Co@Al**_**2**_ **O**_**3**_, yield **2a**/%^a^	**Co@SiO**_**2**_, yield **2a**/%^b^
1	H_2_O	>99	1
2	MeOH	>99	>99
3	EtOH	24	1
4	*i*-PrOH	18	2
5	pyridine	1	n.d.
6	ACN	n.d.	n.d.
7	THF	11	1
8	Et_2_O	23	6
9	EtOAc	26	1
10	CHCl_3_	n.d.	n.d.
11	DCM	21	n.d.
12	1,4-dioxane	30	n.d.
13	toluene	24	1
14	*n*-heptane	21	1

a Reaction conditions (batch): **1a** (0.25 mmol), solvent (2 mL), **Co@Al**_**2**_ **O**_**3**_ (30 mg), 80 °C, H_2_ (50 bar), and 16 h.

b Reaction conditions (batch): **1a** (0.25 mmol), solvent (2 mL), **Co@SiO**_**2**_ (30 mg), 100 °C, H_2_ (50 bar), 20 h. The yields were determined through GCMS analyses with *n*-hexadecane or chlorobenzene serving as internal standard.

Having found a common, optimal solvent for both catalysts, we looked into the recycling capabilities of **Co@Al**_**2**_ **O**_**3**_ and **Co@SiO**_**2**_. For that purpose, the model reaction **1a** → **2a** was performed in MeOH and the catalyst was separated by centrifugation with subsequent decantation of the supernatant liquid. Then, the solid residue was washed with MeOH under sonication, and the catalyst thus recovered was reused in the next iteration **1a** → **2a**. Regarding **Co@Al**_**2**_ **O**_**3**_, it was already spent after the first run, and hence this composite was not amenable to recycling. On the contrary, **Co@SiO**_**2**_ exhibited better longevity and was reusable four times without loss of activity. After the fifth cycle, we noticed incipient deactivation of the catalyst, which is likely due to leaching and/or mechanical abrasion while stirring under elevated temperatures. In a comparison experiment, the benchmark transformation **1a** → **2a** was conducted at prolonged reaction time (96 h), and after this period, the catalyst was subjected to elemental analysis. Indeed, the Co content was reduced from 2.71 to 1.01% which proves that active material gradually detaches from the catalyst during the hydrogenation process.

Following the introductory hydrogenation experiments described above, we studied the impact of the crucial reaction parameters (temperature, H_2_ pressure, reaction time) on the catalyst performance. Through performing Maitlis’ filtration test,^60^ we finally ensured that the quinoline reduction described herein indeed proceeds via a heterogeneous pathway. The details of these probes are delineated in the Supporting Information.

### Scope and Limitations of Co@SiO_2_

The hydrogenation of the parent quinoline **1a** → **2a** was impeccable and most of the CH_3_-tagged congeners were neatly converted into the desired THQ derivatives, too (Scheme 2). Yet, placing the methyl group in proximity to the sp^2^ nitrogen of the heterocycle had a detrimental effect for the product yield (**2h**). Moreover, **1d** with the CH_3_ substituent in the seemingly remote 4 position did not allow for any product formation. Notwithstanding this, our protocol was tolerant of bulky *tert*-butyl (**1i**) and phenyl groups, even when the latter was in the direct vicinity of the N atom (**1j**). However, the catalyst could not cope with the presence of acid motifs (**1k**, **1l**) as the protons were likely to capture any catalytically active hydride species. By contrast, the ester group in **1m** was well tolerated and the corresponding THQ **2m** was isolated in almost quantitative yield. Halides (F, Cl, and Br) were also accommodated, and the catalytic transformations were not hampered by hydrodehalogenation processes; only unsatisfactory yields were obtained in the case of the bromo compounds **1u** and **1v** (27 and 18% isolated yield, respectively). Additionally, electron-releasing donor groups (MeO, NH_2_) did not have any adverse effects on the Co-catalyzed hydrogenation described herein, thus enabling good to excellent yields (**2w**–**z**). Notably, when 1,5-naphtyridine (**1ab**) had been hydrogenated over **Co@SiO**_**2**_ only one pyridine ring was fully reduced, whereas the other one remained unaltered. Generally, the introduced Co-based catalytic system seems to be specific for the quinoline scaffold since isoquinoline **1ac** was not converted under the standard reaction conditions. In the case of cinchonine **1ad**, we found that the C=C bound was hydrogenated (100% substrate conversion), whereas the quinoline core remained fully intact.

**Scheme 2:**
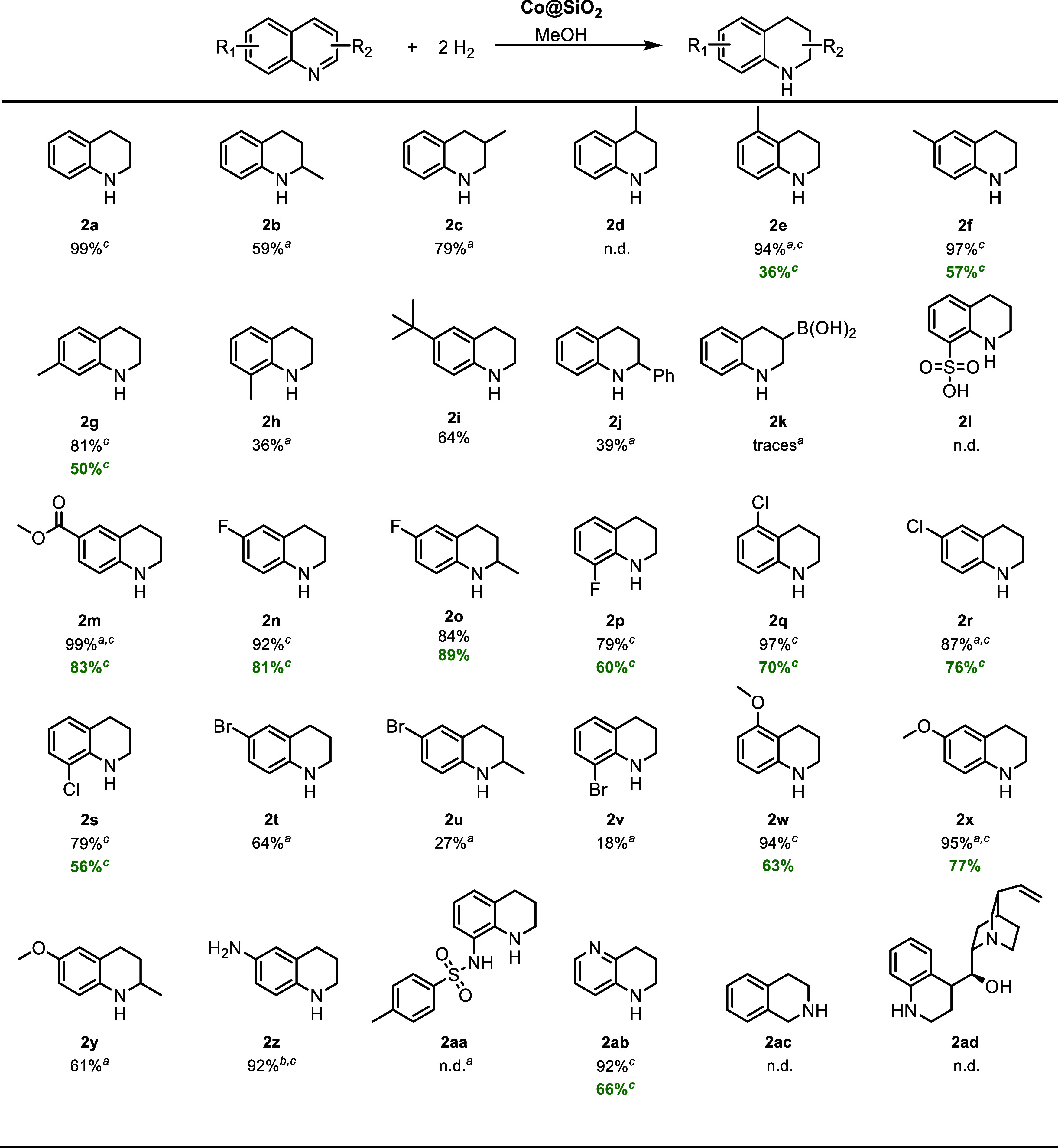
Functional Group Tolerance Survey for the Co-Catalyzed Quinoline Hydrogenation

Standard reaction conditions (batch): substrate (0.5 mmol), MeOH (2 mL), **Co@SiO**_**2**_ (45 mg), 100 °C, H_2_ (40 bar), and 16 h.
a Modified reaction conditions: 120 °C, 20 h, **Co@SiO**_**2**_ (45 mg).
b Modified reaction conditions: 120 °C, 20 h, **Co@SiO**_**2**_ (90 mg). Entries marked in green color refer to continuous-flow hydrogenation. Standard conditions (flow): substrate (0.2 mmol), MeOH (20 mL), 100 °C, H_2_ (50 bar), flow rate: 0.1 mL min^–1^, and 150 mg of catalyst in the cartridge. Entries are presented as isolated yield.
c Complete conversion was proven through GCMS analysis.

Furthermore, we tested our Co catalyst in the continuous flow hydrogenation of selected quinoline substrates, and to our delight, we were able to reproduce the yields from the discontinuous operating mode (Scheme 2, values indicated in green color). However, the discrepancies between GC and isolated yields were larger than that observed in the corresponding batch processes. This is mainly due to the unwanted mixing of the crude THQs with adventitious H_2_O that stems from incomplete electrochemical H_2_ generation inside the flow reactor. Hence, prolonged drying periods were necessary in some cases, which caused a marked decrease in product yields as some THQs were volatile, albeit high-boiling, liquids.

For the purpose of expanding the utility of the **Co@SiO**_**2**_ composite, several (hetero)­arylaldehydes were investigated in Co-catalyzed hydrogenation. The aldehyde-to-alcohol reduction effected by H_2_ gas is a worthwhile chemical transformation as it provides quick access to key intermediates for the manufacture of a host of consumer chemicals.^61–63^

The reduction of benzaldehyde **3a** as well as its monomethylated derivatives **3b**-**d** ran smoothly and the respective alcohols were isolated in good to excellent yields (80–96%). However, the bulky *tert*-butyl group in **3e** provoked a steep loss of product yield (45%), at least in the batch process. Rewardingly, catalytic hydrogenation of the functionalized ester **3f** was flawless, and quantitative amounts of the tagged alcohol were obtained. Regarding the oxygenated substrates **3g**-**i**, the MeO derivatives enabled excellent product yields, whereas the hydrogenation of ortho-vanillin only gave mediocre quantities of the desired alcohol (35%). Obviously, the acidic phenolic OH group next to the aldehyde unit had corrupted the catalytically active species. Note that a similar negative effect of protic groups was identified for quinolines **1k** and **1l** (Scheme 2). The naphthalene derivative **3j** did not pose any problems and the more challenging bromopyridine carbaldehyde **3k** was also convertible into the alcohol to a reasonable extent (60%). On the contrary, the halide-free kindred **4l** was not formed in meaningful amounts.

Once more, a selection of substrates was tested in the flow reactor, and strikingly, the continuous process outperformed the batch hydrogenation in all but one case (Scheme 3, values indicated in green color). The difference in yield is particularly evident for aldehydes **3e** and **3k**. Concerning the discontinuous mode, it is conceivable that the reaction time of 20 h is long enough to allow for consecutive reactions, i.e., deoxygenation and/or hydrodehalogenation once the alcohol has been formed. However, it seems that the shorter residence times in the catalyst cartridges used in the flow hydrogenation circumvented the issue with the ensuing catalytic transformations. Still, for both processes, the GC and isolated yields did not match the vast majority of substrates. This peculiarity can again be traced back to undesired water blending which necessitated extended vacuum treatment of the product/H_2_O mixture (vide supra).

**Scheme 3:**
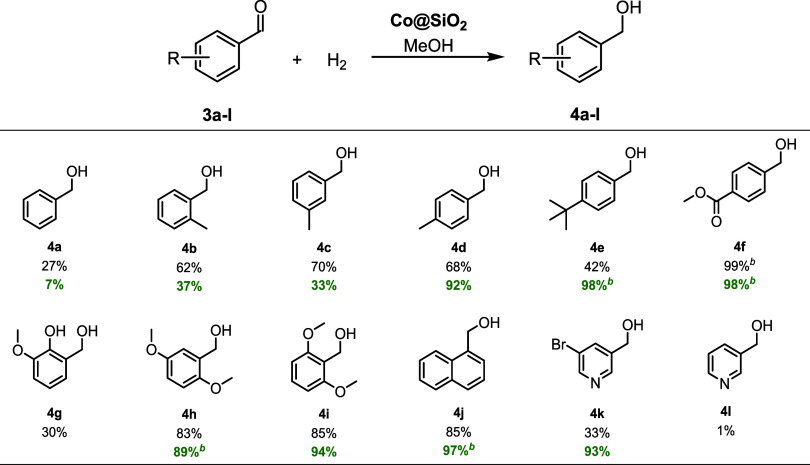
Functional Group Tolerance Survey for the Co-Catalyzed Aldehyde Hydrogenation[Fn s3fn2]

a Standard reaction conditions (batch): substrate (0.5 mmol), MeOH (2 mL), **Co@SiO**_**2**_ (45 mg), 120 °C, H_2_ (40 bar), and 20 h. Entries marked in green color refer to continuous-flow hydrogenation. Standard reaction conditions (flow): substrate (0.2 mmol), MeOH (20 mL), 100 °C, H_2_ (50 bar), flow rate: 0.1 mL min^–1^, and 150 mg of catalyst in the cartridge. Entries are presented as isolated yield. b Complete conversion was proven through GCMS analysis.

Finally, given the fact that various nonprecious-metal-based catalysts, especially those containing cobalt, have been previously reported to promote the (even acceptorless) dehydrogenation of amines^64,65^ and *N*-heterocycles,^66–68^ we deployed our catalyst **Co@SiO**_**2**_ in the reverse manner, i.e., in the THQ-to-quinoline oxidation **2a**→**1a** (Scheme 4). Indeed, the product of the hydrogenation reaction was fully converted back to the starting quinoline on heating in a pressure tube under air. Regrettably, we did not succeed in the somewhat more challenging aerobic oxidation of primary alcohols to the corresponding aldehyde **4m**→**3m**.

**Scheme 4:**
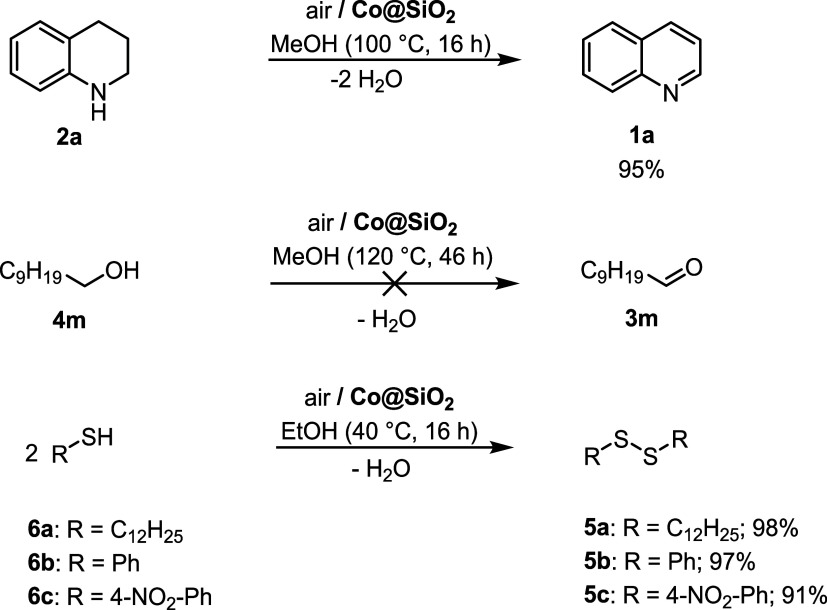
Aerobic Oxidation of Selected Substrates Promoted by Co@SiO_2_ ^a^

a Standard reaction conditions: substrate (0.25 mmol), solvent (2 mL), and **Co@SiO**_**2**_ (25 mg). Entries are presented as isolated yields.

Nevertheless, we managed to synthesize disulfides through the aerobic oxidation of thiols over **Co@SiO**_**2**_. This reaction has some merits on its own because disulfides are promising candidates as cyanide antidotes.^69^ To our delight, aliphatic **6a** as well as the aryl thiols **6b, 6c** were cleanly converted into the desired products under very mild conditions (40 °C). Furthermore, the penetrating smell of the thiols had vanished, which indicated complete conversion of the substrates.

With these preliminary results in hand, we will further evaluate the oxidation capabilities of the pertinent Co catalyst in our laboratories in order to provide a straightforward and robust method for the synthesis of disulfides and heterocycles without recourse to noble metals.^70–73^

## Experimental Section

### Materials and Methods

If not stated otherwise, the commercial chemicals and solvents used in this work were purchased from Acros Organics, Alfa Aesar, BLDpharm, Fluorochem, Macherey-Nagel, Merck, or TCI. The procedural details for reagents and starting materials that were synthesized by ourselves are delineated either in this section or in the Supporting Information part of this publication. Controlled pyrolysis of the impregnated support under Ar (5.0, Linde Gas GmbH) was conducted in an Austromat 626 furnace from Dekema. The batch hydrogenation experiments were done in 300 mL Parr steel autoclaves, whereas the continuous flow reactions were run in an H-Cube Mini Plus reactor from ThalesNano. The catalyst cartridge assembly kit was obtained from the same company and Linde GmbH provided the requisite H_2_ gas (5.0). GCMS analyses were performed on a Shimadzu QP2020 device with an installed SH-Rxi-5Sil MS column (30 m, 0.25 mm, 0.25 μm), whereat electron impact (EI) ionization was achieved at 70 eV and He (5.0, Linde GmbH) served as carrier gas (flow rate 1.7 mL min^–1^). The sample was injected in split mode and processed by way of the following temperature ramp: 50 °C (hold for 1 min) – 100 °C (30 °C min^–1^) – 170 °C (35 °C min^–1^) to 300 °C (40 °C min^–1^, then hold for 1 min). The signals were assigned to the corresponding *m*/*z* ratios, whereby the substrate conversion and product yields (mol %) were calculated from the respective peak areas with the aid of an internal standard (*n*-hexadecane or chlorobenzene). The listed NMR spectra (^1^H, ^13^C, and ^19^F) were recorded on Bruker AVANCE Machines at either 300 or 500 MHz while chemical shifts (δ) of the assigned peaks are given in parts per million (ppm) and were referenced to the residual solvent signal according to the literature.^74^ The coupling constants (*J*) are given in Hz and the following abbreviations were chosen: s-singlet, d-doublet, t-triplet, q-quartet, and m-multiplet. The data pertaining to single crystal X-ray diffraction analysis were collected at 296(2) K on a Bruker D8 Quest Eco diffractometer using graphite monochromated Mo Kα radiation (λ = 0.71073 Å). The results of these measurements, including tabular information, are summarized in the Supporting Information part. Solid state IR spectra were obtained with a Bruker α II Platinum-ATR device and electron paramagnetic resonance (EPR) spectroscopy was performed on a Bruker ELEXYS E580 machine. The EasySpin software package was used for EPR spectrum simulation and analysis.^75^ High resolution mass spectrometry (HRMS) measurements were done through reversed-phase chromatography using a Surveyor HPLC system (Thermo Fisher Scientific) equipped with a Zorbax SB-C18 column from Agilent (150 × 2.1 mm, 5 μm). The column temperature was set to 40 °C and the injection volume amounted to 1 μL. The analytes were separated by gradient elution with mobile phase A containing 0.1% formic acid (FA) in water and mobile phase B with 0.1% FA in acetonitrile at a flow rate of 0.2 mL min^–1^. The elution gradient starting conditions were 95% A and 5% B. After 2 min, the proportion of B was linearly increased to 95% within 25 min, whereupon it was held for a further period of 3 min. High-resolution mass spectra were obtained using an LTQ Orbitrap Velos (Thermo Fisher Scientific) device with APCI and ESI sources operated in positive ionization mode. The resolution was set to 30,000 and diisooctyl phthalate (*m*/*z* = 391.2843) was used as internal standard for mass calibration. Spectra were collected from 100 to 2000 *m*/*z* and data were processed using Xcalibur software (Thermo Fisher Scientific, version 2.2 SP1.48). Elemental analysis was accomplished with a Leco Microanalyzer TruSpec for C, H, N, and S while the metal content was determined by way of atomic absorption spectroscopy (AAS Analyst 300 from PerkinElmer). The XPS (X-ray Photoelectron Spectroscopy) measurements were performed on an ESCALAB 220iXL (Thermo Fisher Scientific) instrument with monochromated Al Kα radiation (*E* = 1486.6 eV). Samples are prepared on a stainless-steel holder with conductive double-sided adhesive carbon tape. The measurements are performed with charge compensation using a flood electron system combining low energy electrons and Ar^+^ ions (p_Ar_ = 1 × 10^–7^ mbar). The electron binding energies are referenced to the C 1s core level of carbon at 284.8 eV (C–C and C–H bonds). For quantitative analysis, the peaks were deconvoluted with Gaussian–Lorentzian curves using Unifit 2023. The peak areas were normalized by the transmission function of the spectrometer and the elemental specific sensitivity factor of Scofield. The scanning transmission electron microscopy (STEM) images were taken with a probe aberration-corrected JEM-ARM200F (JEOL) at an operating voltage of 200 kV. The microscope is equipped with a JED-2300 energy-dispersive X-ray (EDX) spectrometer with a silicon drift detector (dry SD60GV). For general STEM imaging, a High-Angle Annular Dark Field (HAADF) detector and an Annular Bright Field (ABF) detector were applied. The solid powder was deposited without pretreatment on a holey carbon-supported copper grid (mesh 300) and then transferred to the microscope.

### Synthesis of the Solution-Phase Precursor

First, the salen-type ligand **H**_**2**_ **L** was prepared following the rational guidelines from previously published literature procedures.^76,49^ A round-bottom flask (50 mL) was charged with 2,2-dimethyl-1,3-propanediamine **B** (neopentanediamine, 26 mg, 0.25 mmol), which was then dissolved in MeOH (15 mL). After that, 2-hydroxy-3-methoxybenzaldehyde **A** (*ortho*-vanillin, 76 mg, 0.50 mmol) was added while stirring at room temperature upon which the reaction solution immediately turned yellow. The mixture was further agitated overnight, whereupon the volume was reduced under reduced pressure. Hereafter, the formed suspension was filtered, the collected solid washed with ice-cold MeOH (5 mL), and then dried in the desiccator over silica gel. This afforded the ligand as yellow needles (91 mg, 98% yield).

The corresponding dinuclear complex **[Co**_**2**_ **L]** was synthesized according to a report by Appleton.^77^ Initially, compound **H**_**2**_ **L** was prepared in MeOH (12 mL) from aldehyde **A** (304 mg, 2.00 mmol) and diamine **B** (102 mg, 1.00 mmol) following the directions outlined above, whereby the ligand was not isolated in an extra step. The methanolic solution of **H**_**2**_ **L** thus obtained was heated to 50 °C and a suspension of Co­(OAc)_2_·4 H_2_O (301 mg, 1.21 mmol) in H_2_O (3 mL) was added dropwise within 15 min. After a reaction period of 20 h, the volatiles were removed under reduced pressure, and the residue was triturated with H_2_O (3 mL). The resultant precipitate was filtered off and washed with H_2_O (3 mL), leaving behind **[Co**_**2**_ **L]** as brownish-green crystals (297 mg, 44% yield).

### Catalyst Preparation

To a solution of neopentanediamine **B** (33 mg, 0.32 mmol) in methanol (15 mL) were added *ortho*-vanillin **A** (76 mg, 0.50 mmol) and Co­(OAc)_2_·4 H_2_O (76 mg, 0.31 mmol) in that order. The mixture was briefly agitated at room temperature, whereupon solid SiO_2_ (750 mg, particle size range 0.063–0.200 mm) was added portion-wise to the stirred solution over a period of 30 min. On completion of the addition of silica, stirring was continued for 8 h, upon which all volatiles were removed and dried *in vacuo* (3 days). Then, the impregnated support was grinded to a fine powder within 10 min using a pestle and mortar, both made from agate. After that, the charged carrier material was transferred into a ceramic crucible and pyrolyzed under a protective Ar atmosphere (2 h at 800 °C, 25 °C min^–1^ heating rate). Upon cooling to room temperature, the obtained black composite material was again ground (10 min) to afford the ready-to-use catalyst **Co@SiO**_**2**_.

### General Procedure for the Batch Hydrogenations

Without any protection from air, a glass vial (4 mL) was charged with solid catalyst **Co@SiO**_**2**_, the test substrate, and a magnetic stirring bar. Then, a fixed volume (2 mL) of the respective solvent was delivered from a syringe and the reaction flask was sealed with a septum cap. Hereafter, the latter was pierced with a steel cannula and the vial was placed in a drilled Al plate that accommodated up to seven vials. Subsequently, the loaded Al liner was lowered into the pressure vessel, which was tightly screwed and flushed with H_2_ (3 × 20 bar). Upon setting the desired hydrogen pressure, the autoclave was put on a preheated hot plate, and the reaction started. On completion of the hydrogenation experiment, the autoclave was immersed into an ice bath and allowed to reach room temperature. Eventually, the remaining H_2_ gas was released and the reaction mixture filtered, whereupon the clear filtrate was submitted to GCMS analysis. In case an internal standard (*n*-hexadecane or chlorobenzene) was used for the determination of GC yields and conversions, the reference compound was added prior to filtration. If aqueous samples had to be analyzed, ethanol (2 mL) was first added to the reaction mixture, which was then stirred for 10 min. Afterward, analysis proceeded as previously described.

### Product Isolation (Batch)

The solid catalyst was filtered off (Whatman filter), and the clear filtrate evaporated to dryness, thus leaving behind the product. If the residue was still impure, the crude was further purified by way of column chromatography over silica gel, whereby ethyl acetate/*n*-heptane mixtures served as the liquid phase. If tetrahydroquinolines were chromatographed, triethylamine was used to neutralize the stationary phase; the volume ratios were dependent on the used substrate and are stated separately in the Supporting Information.

### General Procedure for the Continuous-Flow Hydrogenations

First, an empty 30 mm cartridge from ThalesNano was filled with the own-developed catalyst **Co@SiO**_**2**_ (150 mg) and then sealed by way of the CatCartPacker assembly kit from the same company. After that, the packed cartridge was inserted into the flow reactor, and the operational parameters (temperature, pressure, flow rate) were set. On stabilization of the system, an Erlenmeyer flask containing the substrate stock solution (0.2 mmol of substrate dissolved in 20 mL of the respective solvent) was connected to the tubing, and the reaction mixture passed through the loaded cartridge. The product flow was continuously analyzed by GCMS whereby *n*-hexadecane or chlorobenzene were used as internal standards. Evaporation of the solvent and, if necessary, column chromatography over (neutralized) silica gel afforded a clean portion of the product (vide supra).

### General Procedure for the Dehydrogenation Reactions

Under standard open flask conditions, a pressure tube was charged with a magnetic stirring bar, solid catalyst **Co@SiO**_**2**_ (25 mg), test substrate (0.25 mmol), and solvent (2 mL) in that order. Then, the tube was tightly sealed and placed in a preheated oil bath whereat the reaction mixture was constantly agitated. After the reaction period had elapsed, the vessel was allowed to reach room temperature, whereupon the reaction mixture was filtered. The clear filtrate was analyzed through GCMS whereby *n*-hexadecane or chlorobenzene were used as internal standards.

## Conclusions

We devised a cheap and user-friendly base metal catalyst that was made through wet impregnation of silica, followed by controlled pyrolysis of the loaded carrier material under an argon atmosphere. The requisite solution-phase precursor was readily assembled prior to use from *ortho*-vanillin, neopentanediamine, and hydrus cobalt acetate. The obtained composite material was then tested in the heterogeneous hydrogenation of quinolines and aldehydes, whereby the intrinsically high metal content of the starting Co complex gave rise to decent product yields. Noteworthy, our catalyst was apt for use in batch hydrogenation as well as in continuous flow mode.

The potential of the deployed double Schiff-base ligand to function as a platform for the pyrolytic syntheses of tailored, heterogeneous catalysts is currently being further explored in our laboratories, especially with respect to aerobic oxidation reactions.

## Supplementary Material



## Data Availability

The data underlying this study are available in the published article and its Supporting Information.

## Notes

Crystallographic data, elemental analyses, influence of the support and the temperature on the hydrogenation of quinoline, yield/time curves, hot filtration test, reusability study, influence of reaction parameters on the continuous flow hydrogenation, catalytic oxidation of thiols (solvent variation study), spectra (IR, UV–vis, EPR, XPS, HRMS, ^1^H, ^13^C {^1^H}, and ^19^F NMR) (PDF)
