# Supplementary material for: Hydrogenation of Quinolines and Aldehydes Catalyzed by a Pyrolyzed, Augmented Cobalt-Salen Complex
Source: ACS Omega. 2025 Aug 6;10(32):36455–65. doi: 10.1021/acsomega.5c04940 (PMC12368676; doi:10.1021/acsomega.5c04940)
Supplement: Supplementary file 1 [file ao5c04940_si_001.pdf]

Supporting Information

Hydrogenation of Quinolines and Aldehydes Catalyzed by a Pyrolyzed, Augmented Cobalt-Salen Complex

Fabian Schmiedbauer[§], Uwe Monkowius[∫], Clemens Schwarzinger[ǂ], Stefan Müllegger[#], Stephan Bartling[†], Nils Rockstroh[†], and Christoph Topf[‡,*]

[§]Institute of Inorganic Chemistry, Johannes Kepler University (JKU), Linz 4040, Austria

[∫]Linz School of Education, Johannes Kepler University (JKU), Linz 4040, Austria

[ǂ]Institute for Chemical Technology of Organic Materials (CTO), Johannes Kepler University (JKU), Linz 4040, Austria

[#]Institute of Semiconductor and Solid State Physics, Solid State Physics Division Johannes Kepler University (JKU), Linz 4040, Austria

[†]Leibniz Institute for Catalysis at the University of Rostock (LIKAT), Rostock 18059, Germany

[‡]Institute of Catalysis (INCA), Johannes Kepler University (JKU), Linz 4040, Austria

*Corresponding Author: christoph.topf@jku.at

Content

1. Crystallographic Data 2

2. Elemental Analyses ....Error! Bookmark not defined.

3. Characterization of **[Co_2L]** through IR, UV/Vis, and EPR Spectroscopy..4

4. XPS Measurement of the Full-Fledged **Co@SiO_2** Catalyst 7

5. Influence of the Support on the Hydrogenation of Quinoline 9

6. Influence of the Temperature on the Hydrogenation of Quinoline Promoted by **Co@support**……….....10

7. Control Experiments of the Solid **Co@SiO_2** Catalyst (Yield/Time Curve, Hot Filtration Test, Reusability Study)..11

8. Influence of the Reaction Conditions on the Continuous-Flow Hydrogenation.....................................14

9. Catalytic Oxidation of Thiols……………………………………………………….……………….....15

10. Procedural Details 17

11. Characterization Data..18

12. References……………………..…………………………………………………………………85

1. Crystallographic Data

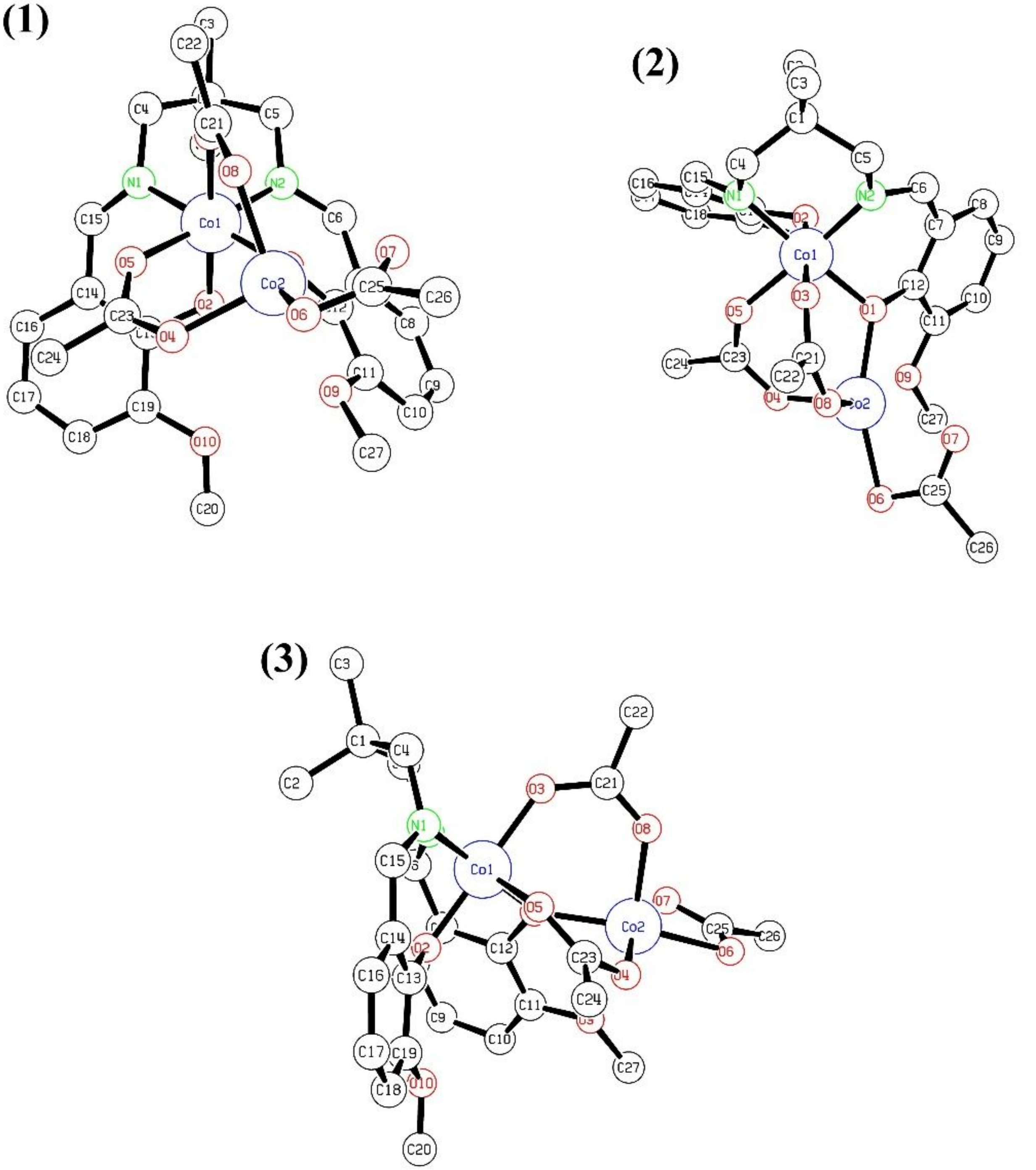

Figure S1. Molecular structure of [Co_2L] as obtained from single crystal X-Ray diffraction analysis viewed from the front (1), from the top (2), and from the left side (3).

Table S1. Crystallographic Data and Refinement of [Co_2L] as Determined by Single Crystal X-Ray Diffraction Analysis

**Compound**	**[Co_2L]**
Empirical formula	$C_{27}H_{35}Co_2N_2O_{10}$
Formula weight	663.41
Crystal size (mm)	0.44 × 0.42 × 0.09 mm
Appearance	plate, light brown
Crystal system	monoclinic
Space group	$P2_1/c$
a (Å)	14.537(3)
b (Å)	12.199(2)
c (Å)	17.531(3)
β (°)	101.910(8)
Z	4
Cell volume ($Å^3$)	3042.0(10)
D_{calc} (g cm^{-3})	1.449
θ (°)	2.4 – 22.0
μ (mm^{-1})	1.15
No. of reflections	102603
No. of independent reflections	5417
No. of reflections with $I > 2\sigma(I)$	3122
R_{int}	0.128
Final R_1 $[I > 2\sigma(I)]$	0.092
Final $wR_2(F^2)$ $[I > 2\sigma(I)]$	0.195
Final R_1 *(all data)*	0.156
Final $wR_2(F^2)$ *(all data)*	0.217

Δp_{min}, Δp_{max} (e Å^{-3})	-0.63, 0.92
CCDC	2449798

2. Elemental Analyses

Table S2. Results of the Elemental Analysis

Entry	Co / %	C / %	H / %	N / %
1[a]	17.77	47.15	4.07	3.69
2[a]	17.83	47.43	4.21	3.73
3[b]	2.73	3.93	0.10	0.18
4[b]	2.70	4.00	0.09	0.19

[a]Elemental analysis of the isolated solution phase precursor **[Co_2L]**; [b]Elemental analysis of the full-fledged catalyst **Co@SiO_2**.

3. Characterization of [Co_2L] by IR, UV/Vis, and EPR Spectroscopy

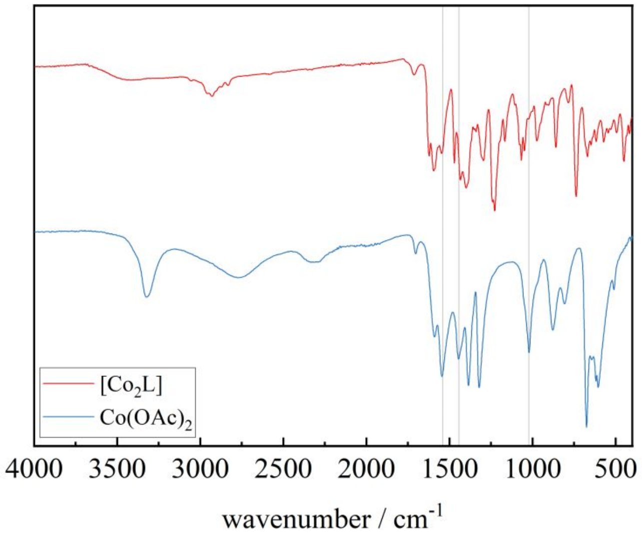

Figure S2. ATR-FTIR spectra of **[Co_2L]** (prepared under ambient conditions) and unmodified $Co(OAc)_2$. The bands characteristic of the acetate group in $Co(OAc)_2$, i.e., 1546 cm^{-1}, 1442 cm^{-1}, and 1025 cm^{-1},[1] (blue trace) are partially shifted in the metal complex **[Co_2L]** indicating the presence of the AcO groups that are now likely in a different coordination environment.

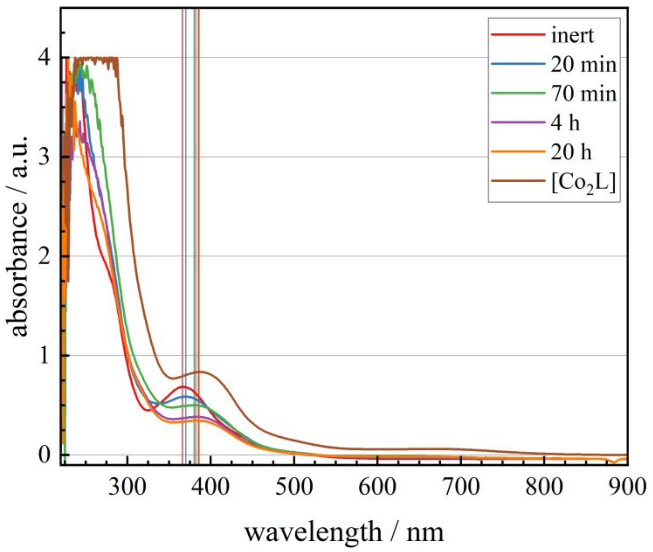

Figure S3. UV/Vis spectrum of **[Co_2L]** prepared under Ar followed by exposure to air for varying intervals together with the spectrum of **[Co_2L]** synthesized without any protection from air. The absorption maximum of the complex prepared under inert conditions is at 366 nm. Exposure to air lead to a continuous bathochromic shift toward the characteristic spectroscopic feature of **[Co_2L]** (388 nm) that was prepared under ambient conditions.

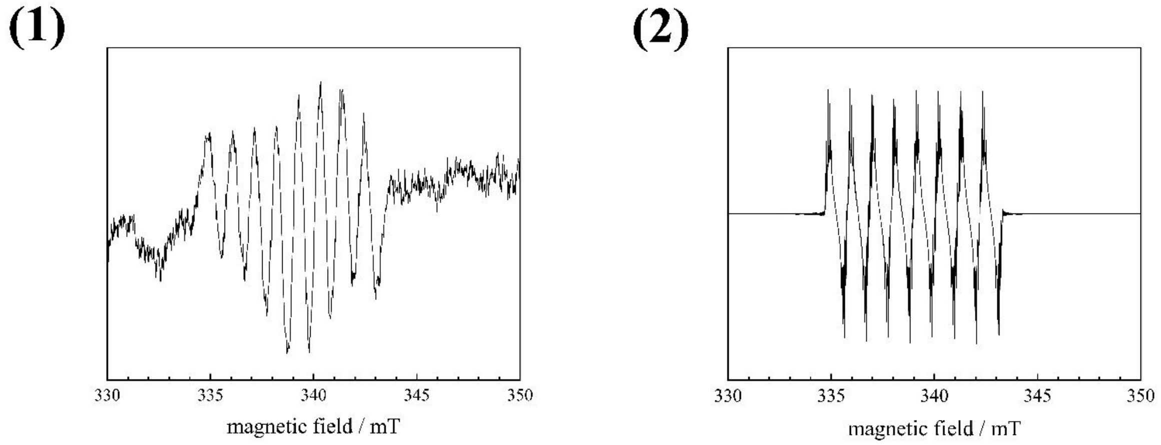

Figure S4. EPR spectra of **[Co_2L]** in $CDCl_3$ measured at 27 ˚C (1) and a simulated spectrum calculated by EasySpin[2] (2).

The measured spectrum indicates a paramagnetic species which caused an octet signal around a magnetic field strength of 340 mT which probably stems from hyperfine splitting due to the orbiting electrons of the surrounding nuclei (Scheme 3, (1)). On using EasySpin, an isotropic system was computed considering a central Co atom bound to two N^{14} atoms and two O^{16} atoms. The resulting spectra showed a similar octet at a comparable magnet field strength, hinting towards the proposed structure. The g-factor was calculated using the approximation shown in Equation (1) which is in accordance with the literature values (g = 2.0028 reported by Rindone and coworkers)[3] as well as with a value of g = 2.0019 obtained from a least-squares fit of the experimental spectrum with the help of EasySpin package assuming isotropic cw EPR and linear background.

$$g = 0.714489 \cdot \frac{\text{frequency [GHz]}}{\text{magnetic field strength [G]}} = 0.714489 \cdot \frac{9.49882}{3.390} = 2.002 \qquad (1)$$

Files:

HC007.DTA

EPR_Fit.m

4. XPS Measurement of the Full-Fledged Co@SiO_2 Catalyst

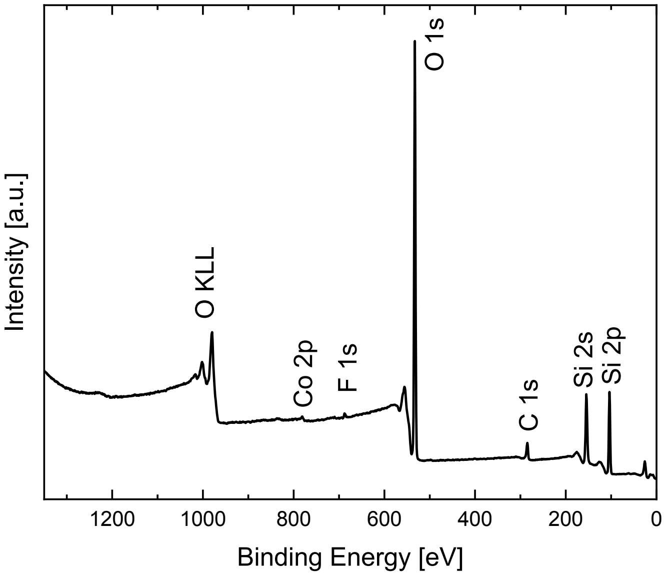

Figure S5. XPS survey spectrum of solid **Co@SiO_2**.

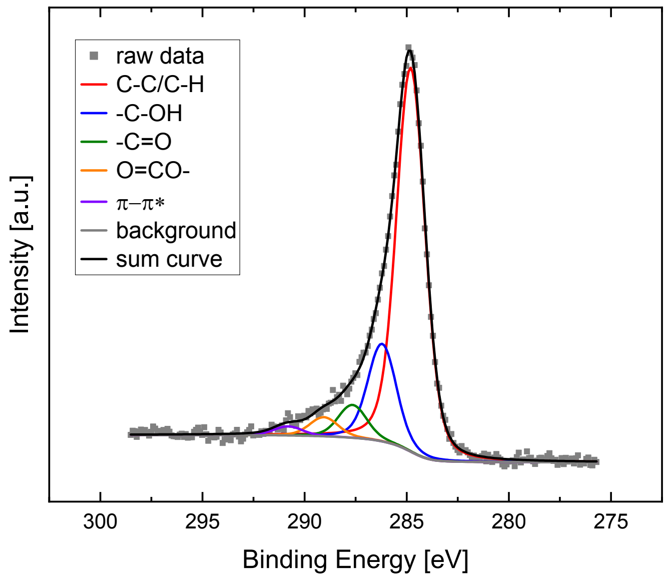

Figure S-XPS1. XPS analysis of the C 1s region of sample **Co@SiO$_2$**.

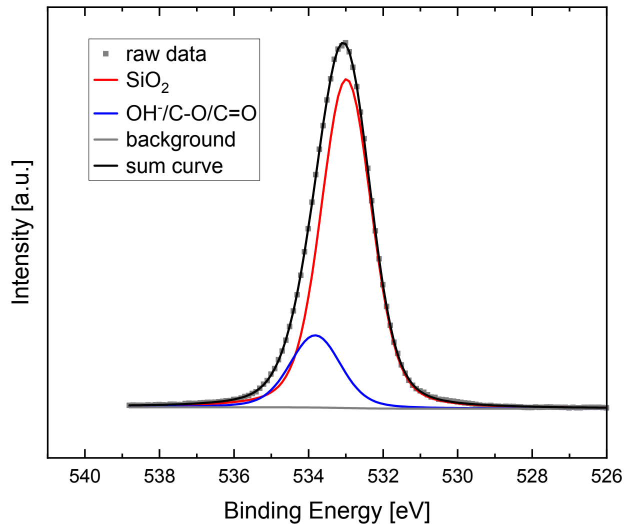

Figure S-XPS2. XPS analysis of the O 1s region of **Co@SiO$_2$**.

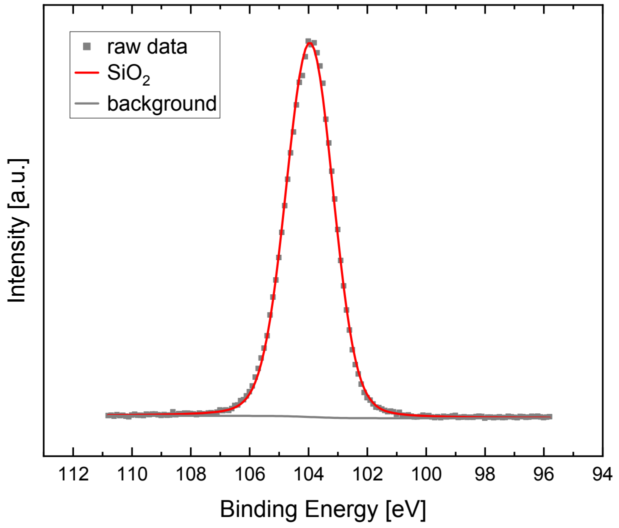

Figure S-XPS3. XPS analysis of the Si 2p region of **Co@SiO$_2$**.

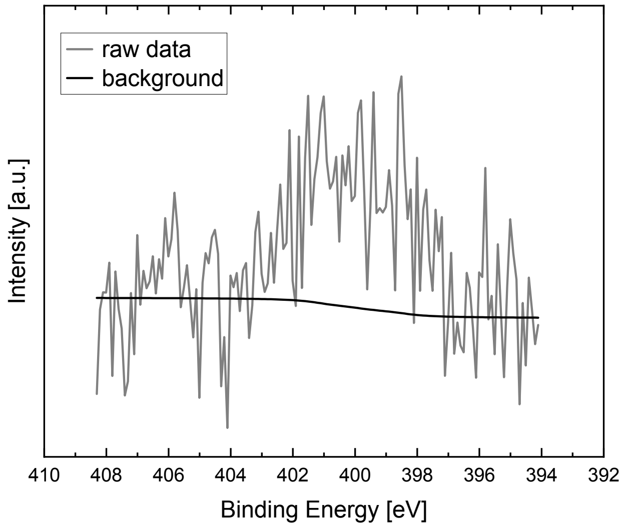

Figure S-XPS4. XPS measurement of the N 1s region of the sample **Co@SiO$_2$.**

5. Influence of the Support on the Hydrogenation of Quinoline

Table S3. Influence of the Support on the Co-Catalyzed Quinoline Hydrogenation[a]

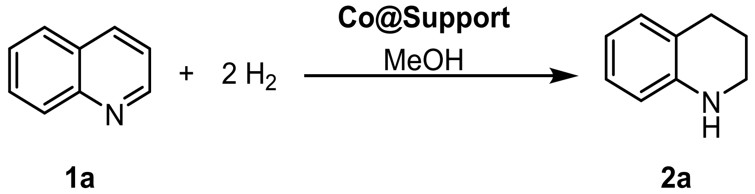

Entry	Support	Yield 2a / %
1	SiO_2 (0.063 - 0.200 mm)	12
2	SiO_2 (0.04 - 0.064 mm)	0
3	SiO_2 (10 - 20 nm)	0
4	MgO (< 50 nm)	4
5	CeO_2 (5 µm)	0
6	Vulcan	8
7	activated α-Al_2O_3 (< 1 µm)	42
8[b]	γ-Al_2O_3	0
9[b]	activated acidic Al_2O_3 (0.063 - 0.200 mm)	9
10[b]	activated basic Al_2O_3 (0.063 - 0.200 mm)	9
11[b]	activated acidic Al_2O_3 (60 mesh powder)	4
12[b]	basic Al_2O_3 (50 - 200 nm)	21
13[b]	activated neutral Al_2O_3 (50 - 200 micron)	11
14[b]	activated neutral Al_2O_3 (60 mesh powder)	5

[a]Reaction conditions (batch): **1a** (0.25 mmol), MeOH (2 mL), **Co@support** (15 mg), 80 ˚C, H_2 (50 bar), and 16 h reaction time. [b]Alternative conditions: **1a** (0.25 mmol), MeOH (2 mL), **Co@support** (22.5 mg), 100 ˚C, H_2 (50 bar), and 16 h reaction time. The yield of **2a** was determined by way of GCMS analysis whereby *n*-hexadecane served as internal standard. The particle sizes are given in parentheses; n.d. means 'not detected'.

6. Influence of the Temperature on the Hydrogenation of Quinoline Promoted by Co@support

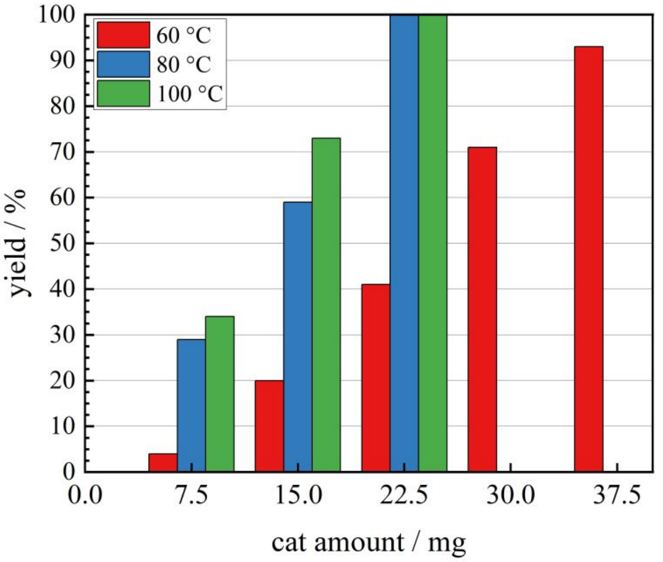

Figure S6. Effect of different temperatures on the hydrogenation of quinoline **1a** (0.25 mmol) catalyzed by **Co@Al$_2$O$_3$** in H_2O (2 mL) at 50 bar H_2 (16 h). The yields were determined by means of GCMS using *n*-hexadecane as internal standard.

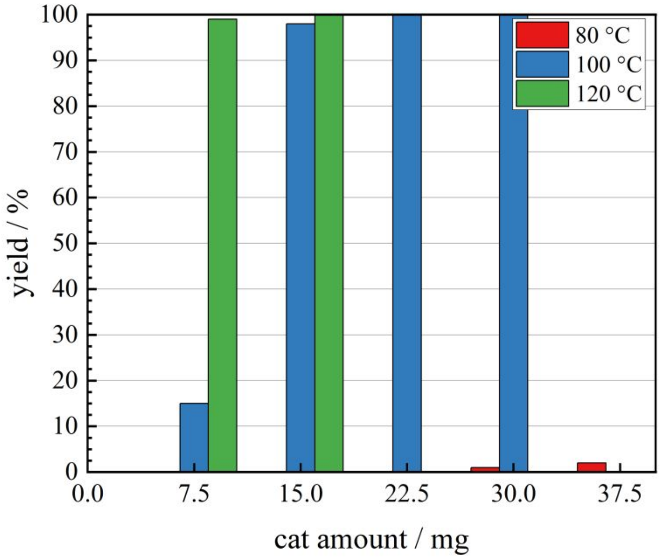

Figure S7. Temperature variation study conducted with quinoline **1a** (0.25 mmol) **Co@SiO$_2$** in 2 mL MeOH (50 bar H_2, 16 h). The yields were determined by means of GCMS using *n*-hexadecane as internal standard.

7. Control Experiments of the Solid Co@SiO$_2$ Catalyst (Yield/Time Curve, Hot Filtration Test, Reusability Study)

Table S4. Deviations from the Standard Catalytic Protocol

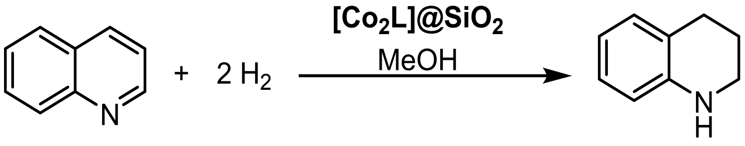

1a **2a**

Entry	Catalyst deviation	Yield 2a / %
1	none	98
2	**L@SiO$_2$** (0.063 - 0.200 mm)	0
3	**Co@SiO$_2$** (0.063 - 0.200 mm)	2
4	SiO_2 (0.063 - 0.200 mm)	0
5[a]	**[Co$_2$L]**	0
6[b]	Co^0 (2 µm)	11
7[c]	Co_3O_4 (powder)	3

Reaction conditions (batch): **1a** (0.25 mmol), MeOH (2 mL), **Co@SiO$_2$** (15 mg), 100 °C, H_2 (50 bar), and 16 h. The yield of **2a** was determined by way of GCMS analysis whereby *n*-hexadecane served as internal standard. The particle sizes are given in parentheses. [a]A quantity of 2.3 mg of the complex was used as homogeneous catalyst. [b]An amount of 0.5 mg was used under inert conditions. [c]A quantity of 0.6 mg was applied.

To determine the amount of catalyst (Table S4, entries 5 – 7) the amount of Co (2.71 %) on **Co@SiO$_2$** was considered and a molar equivalent of metal was used.

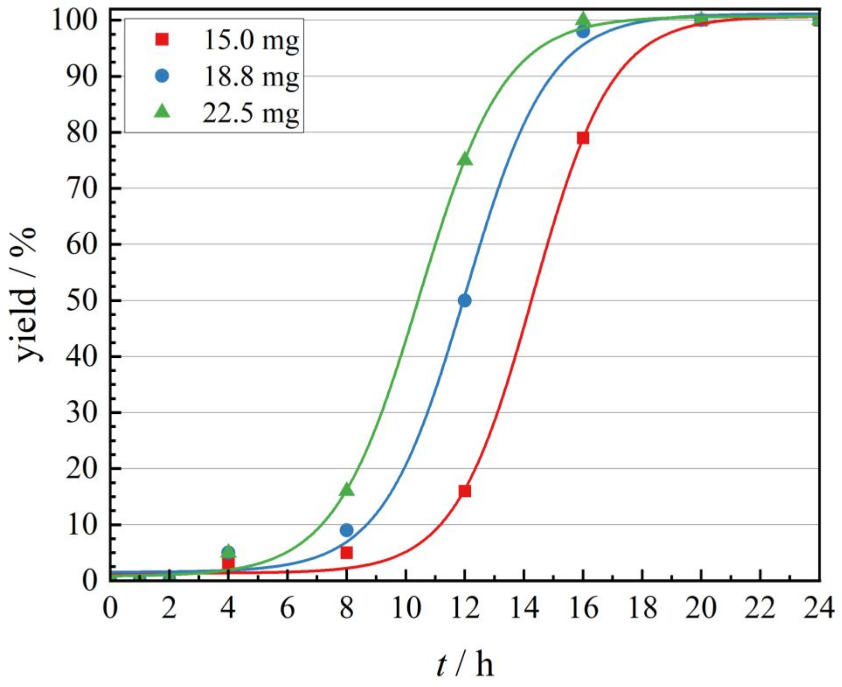

Figure S8. Yield / time curve recorded for varying amounts of **Co@SiO$_2$** (0.25 mmol 1a, 2 mL MeOH, 40 bar H_2, and 80 °C. The yields were determined by means of GCMS using *n*-hexadecane as internal standard.

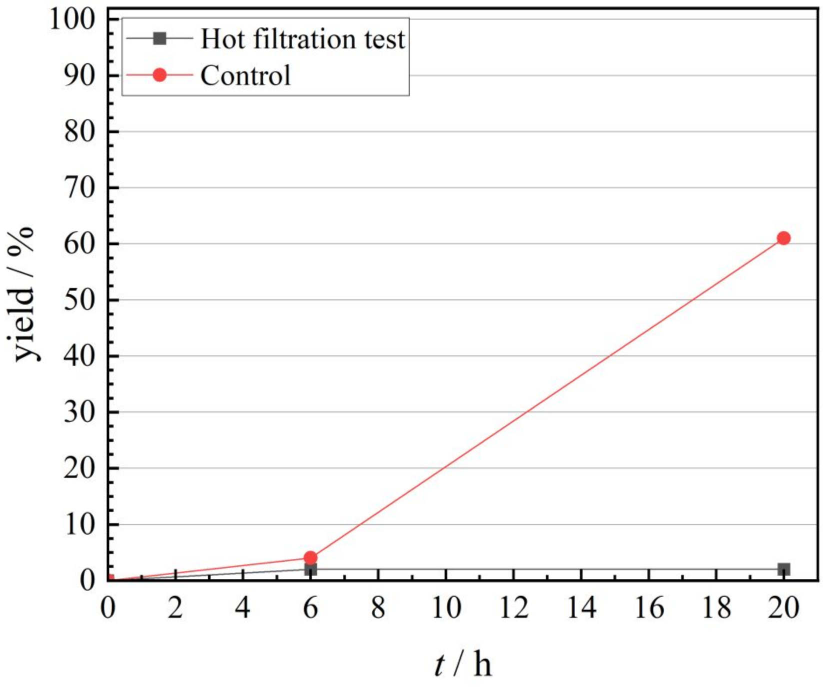

Scheme S9. Hot filtration test performed with **1a** (0.25 mmol) in MeOH (2 mL) at 100 ˚C and 40 bar H_2 in the presence of **Co@SiO$_2$** (22.5 mg). The yields were determined by means of GCMS using *n*-hexadecane as internal standard.

For the hot filtration test a standard hydrogenation experiment was set up as described earlier. The reaction was then halted after 6 h whereupon the catalyst was filtered off under inert conditions. After that the quinoline hydrogenation was resumed and continued until expected full conversion. A control experiment that left out the filtration step was performed in parallel and Scheme S9 clearly indicates that a heterogeneous scenario.[4]

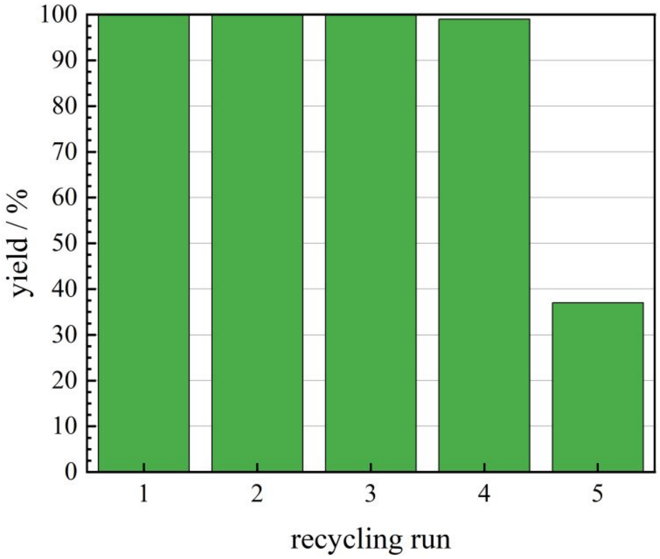

Figure S10. Reusability study performed with **1a** (0.25 mmol) in MeOH (2 mL) at 100 ˚C, 40 bar H_2, and 16 h in the presence of **Co@SiO$_2$** (22.5 mg). The yields were determined by means of GCMS using *n*-hexadecane as internal standard.

8. Influence of the Reaction Conditions on the Continuous-Flow Hydrogenation

Table S5. Optimization of the Reaction Conditions for the Continuous-Flow Hydrogenation of 1a

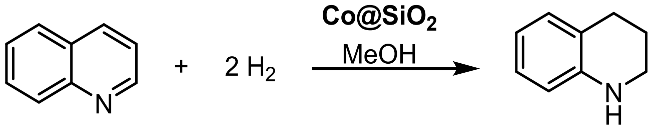

1a **2a**

Entry [a]	*T* / ˚C	Yield **2a** / %	Entry [b]	*p* / bar	Yield **2a** / %
1	60	14	4	30	63

2	80	92	5	40	92
3	100	>99	6	50	>99

[a]Standard conditions (flow): **1a** (0.2 mmol), MeOH (20 mL), H_2 (50 bar), flow rate: of 0.1 mL min^{-1}, and 150 mg of catalyst in the cartridge. [b]Alternative conditions: **1a** (0.2 mmol), MeOH (20 mL), 100 °C, flow rate: of 0.1 mL min^{-1}, and 150 mg of catalyst in the cartridge. The yields were determined through GCMS analyses with *n*-hexadecane serving as internal standard.

9. Catalytic Oxidation of Thiols

Table S6. Solvent Variation Study for the Oxidation of 1-Dodecanethiol 6a

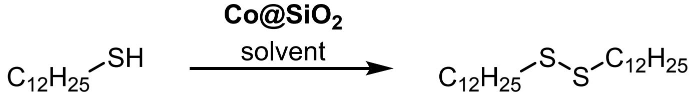

6a **5a**

Entry	Solvent	Yield **5a** / %
1	H_2O	4
2	MeOH	29
3	EtOH	48
4	*i*-PrOH	10
5	pyridine	29
6	ACN	4
7	THF	1
8	Et_2O	1
9	EtOAc	1
10	$CHCl_3$	2
11	DCM	2
12	1,4-dioxane	1
13	toluene	2
14	*n*-heptane	4

Standard reaction conditions: **6a** (0.25 mmol), solvent (2 mL), **Co@SiO$_2$** (6.2 mg), room temperature, and 16 h.

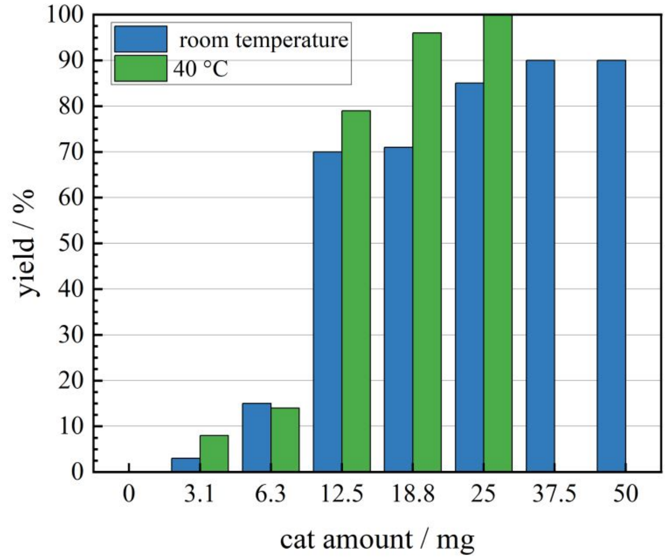

Figure S11. Temperature variation study performed with **6a** (0.25 mmol) in EtOH (2 mL) in the presence of varying amounts of **Co@SiO$_2$** (16 h). The yields were determined by means of GCMS Analyses using *n*-hexadecane as internal standard.

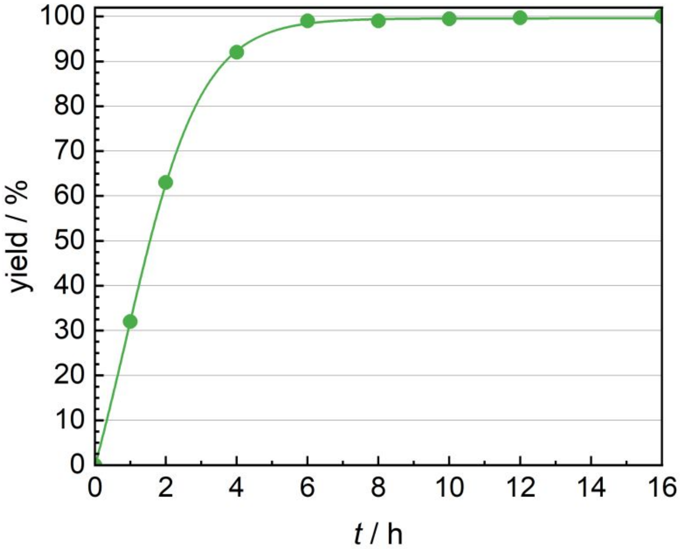

Figure S12. Yield / time curve recorded using 0.25 mmol of **6a** in 2 mL pyridine at 40 ˚C in the presence of **$Co@SiO_2$** (25 mg). The yields were determined by GCMS analyses using *n*-hexadecane as internal standard.

10. Procedural Details

General Procedure for the Batch Hydrogenation: The hydrogenation experiments were set up as described in the main text (Experimental Section). Reaction conditions **A**: substrate (0.5 mmol), MeOH (2 mL), **$Co@SiO_2$** (45 mg), 100 °C, H_2 (40 bar), and 16 h. Reaction conditions **B**: substrate (0.5 mmol), MeOH (2 mL), **$Co@SiO_2$** (45 mg), 120 °C, H_2 (40 bar), and 20 h. Reaction conditions **C**: substrate (0.5 mmol), MeOH (2 mL), **$Co@SiO_2$** (90 mg), 120 °C, H_2 (40 bar), and 20 h. The product was isolated and precipitated by HCl (1M in Et_2O) using DCM as solvent.

General Procedure for the Flow Hydrogenation: The hydrogenation experiments were set up as described in the main text (Experimental Section). Reaction conditions: substrate (0.2 mmol), MeOH (20 mL), 100 ˚C, H_2 (50 bar), flow rate: 0.1 mL min^{-1}, and 150 mg of solid catalyst in the cartridge.

General Procedure for the Batch Dehydrogenation Reactions: The dehydrogenation experiments were set up as described in the main paper (Experimental Text). Reaction conditions: substrate (0.5 mmol), EtOH (2 mL), **$Co@SiO_2$** (50 mg), 40 ˚C, and 16 h.

Characterization of the Substrates: NMR spectra were recorded on a Magnet System 300 MHz / 54 mm Ultrashield from Bruker. The spectra were recorded at 300 MHz for ^{1}H NMR, 75.5 MHz for 13C NMR, and 282 MHz for ^{19}F NMR. The xxes were calibrated using the residual nondeuterated solvent peak for ^{1}H and $^{13}C\{^{1}H\}$ NMR as referenced on the delta scale (δ) with chemical shift listed in parts per million (ppm).

Mass spectra were obtained by way of HPLC-MS measurements using reversed-phase chromatography and an ESI ion source.

11. Characterization Data

*1,2,3,4-tetrahydroquinoline (**2a**).* The title compound was synthesized according to procedure **A**. 65.9 mg (0.51 mmol) of **1a** were used, 45.1 mg catalyst. Appearance: yellow oil, 67.2 mg (0.505 mmol, 99% yield). Analytical data: ^{1}H NMR (300 MHz, $CDCl_3$, 25 °C): δ = 7.01 – 6.95 (m, 2H), 6.64 – 6.59 (td, J = 7.4, 1.1 Hz, 1H), 6.50 – 6.47 (d, J = 7.8 Hz, 1H), 3.81 (s, 1H), 3.33 – 3.29 (m, 2H), 2.80 – 2.76 (t, J = 6.4 Hz, 2H), 2.00 – 1.92 (m, 2H) ppm; $^{13}C\{^1H\}$ NMR (75.5 MHz, $CDCl_3$, 25 °C): δ = 144.9, 129.6, 126.8, 121.5, 117.0, 114.3, 42.1, 27.1, 22.3 ppm. HRMS (APCI-Orbitrap) *m/z*: $[MH]^+$ calc. for $C_9H_{12}N$: 134.0970; found: 134.0983.

*2-methyl-1,2,3,4-tetrahydroquinoline (**2b**).* The title compound was synthesized according to procedure **B**. 73.7 mg (0.515 mmol) of **1b** were used, 45.5 mg catalyst. The product was purified through column chromatography (silica gel, EtOAc/*n*-heptane 1:10, 5% triethylamine). Appearance: clear oil, 44.7 mg (0.304 mmol, 59% yield). Analytical data: ^{1}H NMR (300 MHz, $CDCl_3$, 25 °C): δ = 7.01 – 6.96 (m, 2H), 6.65 – 6.60 (td, J = 7.4, 1.1 Hz, 1H), 6.50 – 6.47 (m, 1H), 3.70 (s, 1H), 3.47 – 3.37 (m, 1H), 2.92 – 2.70 (m, 2H), 1.99 – 1.91 (m, 1H), 1.68 – 1.54 (m, 1H), 1.24 – 1.22 (d, J = 6.3 Hz, 3H) ppm; $^{13}C\{^1H\}$ NMR (75.5 MHz, $CDCl_3$, 25 °C): δ = 144.4, 129.6, 126.8, 121.2, 117.0, 114.0, 49.0, 35.6, 27.3, 19.2 ppm. HRMS (APCI-Orbitrap) *m/z*: $[MH]^+$ calc. for $C_{10}H_{14}N$: 148.1126; found: 148.1119.

*3-methyl-1,2,3,4-tetrahydroquinoline (**2c**).* The title compound was synthesized according to procedure **B**. 69.0 mg (0.482 mmol) of **1c** were used, 45.3 mg catalyst. The product was purified through column chromatography (silica gel, *n* EtOAc/*n*-heptane 1:10, 5% triethylamine). Appearance: yellow oil, 56.0 mg (0.380 mmol, 79% yield). Analytical data:^{1}H NMR (300 MHz, $CDCl_3$, 25 °C): δ = 7.02 – 6.96 (m, 2H), 6.66 – 6.61 (td, J = 7.4, 1.1 Hz, 1H), 6.52 – 6.49 (d, J = 8.0 Hz, 1H), 3.85 (s, 1H), 3.31 – 3.26 (m, 1H), 2.95 – 2.88 (m, 1H), 2.84 – 2.77 (dd, J = 4.9, 1.7 Hz, 1H), 2.50 – 2.41 (m, 1H), 2.14 – 2.02 (m, 1H), 1.09 – 1.06 (d, J = 6.6 Hz, 3H) ppm; $^{13}C\{^1H\}$ NMR (75.5 MHz, $CDCl_3$, 25 °C): δ = 144.9, 129.4, 126.8, 121.2, 117.1, 114.1, 47.3, 30.2, 26.7, 22.7 ppm. HRMS (APCI-Orbitrap) *m/z*: $[MH]^+$ calc. for $C_{10}H_{14}N$: 148.1126; found: 148.1120.

*5-methyl-1,2,3,4-tetrahydroquinoline (**2e**).* The title compound was synthesized according to procedure **B**. 72.7 mg (0.508 mmol) of **1e** were used, 45.4 mg catalyst. Appearance: yellow oil, 70.0 mg (0.475 mmol, 94% yield). Analytical data: ^1H NMR (300 MHz, $CDCl_3$, 25 °C): δ = 6.97 – 6.92 (t, J = 7.7 Hz, 1H), 6.59 – 6.56 (d, J = 7.4 Hz, 1H), 6.43 – 6.40 (d, J = 8.0 Hz, 1H), 3.78 (s, 1H), 3.32 – 3.28 (m, 2H), 2.72 -2.67 (t, J = 6.6 Hz, 2H), 2.23 (s, 1H), 2.08 – 2.00 (m, 2H) ppm; $^{13}C\{^1H\}$ NMR (75.5 MHz, $CDCl_3$, 25 °C): δ = 145.0, 137.2, 126.2, 120.2, 119.0, 112.5, 41.7, 24.1, 22.6, 19.4 ppm. HRMS (APCI-Orbitrap) *m/z*: $[MH]^+$ calc. for $C_{10}H_{14}N$: 148.1126; found: 148.1119.

*6-methyl-1,2,3,4-tetrahydroquinoline (**2f**).* The title compound was synthesized according to procedure **A**. 70.3 mg (0.491 mmol) of **1f** were used, 45.3 mg catalyst. Appearance: clear oil, 70.1 mg (0.476 mmol, 97% yield). Analytical data: ^1H NMR (300 MHz, $CDCl_3$, 25 °C): δ = 6.82 – 6.80 (m, 2H), 6.44 – 6.42 (m, 1H), 3.70 (s, 1H), 3.31 – 3.27 (m, 2H), 2.78 – 2.73 (t, J = 6.4 Hz, 2H), 2.23 (s, 3H), 1.99 – 1.91 (m, 2H) ppm; $^{13}C\{^1H\}$ NMR (75.5 MHz, $CDCl_3$, 25 °C): δ = 142.5, 130,2 127.3, 126.3, 121.7, 114.5, 42.3, 27.0, 22.5, 20.5 ppm. HRMS (APCI-Orbitrap) *m/z*: $[MH]^+$ calc. for $C_{10}H_{14}N$: 148.1126; found: 148.1120.

*7-methyl-1,2,3,4-tetrahydroquinoline (**2g**).* The title compound was synthesized according to procedure **A**. 69.1 mg (0.487 mmol) of **1g** were used, 45.3 mg catalyst. Appearance: clear oil, 57.9 mg (0.393 mmol, 81% yield). Analytical data: ^1H NMR (300 MHz, $CDCl_3$, 25 °C): δ = 6.87 – 6.84 (d, J = 7.6 Hz, 1H), 6.47 – 6.44 (td, J = 7.6, 1.1 Hz, 1H), 6.32 (s, 1H), 3.76 (s, 1H), 3.31 – 3.28 (m, 2H), 2.76 – 2.72 (t, J = 6.4 Hz, 2H), 2.23 (s, 3H), 1.98 – 1.90 (m, 2H) ppm; $^{13}C\{^1H\}$ NMR (75.5 MHz, $CDCl_3$, 25 °C): δ = 144.7, 136.5, 129.5, 118.7, 118.0, 114.9, 42.1, 26.7, 22.5, 21.2 ppm. HRMS (APCI-Orbitrap) *m/z*: $[MH]^+$ calc. for $C_{10}H_{14}N$: 148.1126; found: 148.1121.

*8-methyl-1,2,3,4-tetrahydroquinoline (**2h**).* The title compound was synthesized according to procedure **B**. 72.4 mg (0.506 mmol) of **1h** were used, 45.6 mg catalyst. The product was purified through column chromatography (silica gel, EtOAc/*n*-heptane 1:10, 5% triethylamine). Appearance: yellow oil, 27.0 mg (0.183 mmol, 36% yield). Analytical data:^1H NMR (300 MHz, $CDCl_3$, 25 °C): δ = 6.91 – 6.86 (m, 2H),

6.60 – 6.55 (t, *J* = 7.4 Hz, 1H), 3.66 (s, 1H), 3.41 – 3.37 (m, 2H), 2.83 – 2.29 (t, *J* = 6.4 Hz, 2H), 2.10 (s, 3H), 2.00 – 1.92 (m, 2H) ppm; $^{13}C\{^1H\}$ NMR (75.5 MHz, $CDCl_3$, 25 °C): δ = 142.8, 128.0, 127.5, 121.3, 121.0, 116.5, 42.5, 27.4, 22.3, 17.3 ppm. HRMS (APCI-Orbitrap) *m*/*z*: $[MH]^+$ calc. for $C_{10}H_{14}N$: 148.1126; found: 148.1119.

*6-(tert-butyl)-1,2,3,4-tetrahydroquinoline (**2i**).* The title compound was synthesized according to procedure **A**. 91.6 mg (0.494 mmol) of **1i** were used, 45.4 mg catalyst. The product was purified through column chromatography (silica gel, EtOAc/*n*-heptane 1:10, 5% triethylamine). Appearance: yellow oil, 60.0 mg (0.317 mmol, 64% yield). Analytical data: ^1H NMR (300 MHz, $CDCl_3$, 25 °C): δ = 7.04 – 7.00 (m, 2H), 6.48 – 6.46 (d, *J* = 8.3 Hz, 1H), 3.71 (s, 1H), 3.31 – 3.29 (m, 2H), 2.80 – 2.78 (t, *J* = 6.4 Hz, 2H), 2.00 – 1.95 (m, 2H), 1.30 (s, 9H) ppm; $^{13}C\{^1H\}$ NMR (75.5 MHz, $CDCl_3$, 25 °C): δ = 142.6, 140.0, 126.4, 123.8, 121.0, 114.3, 42.2, 33.9, 31.7, 27.3, 22.6 ppm. HRMS (APCI-Orbitrap) *m*/*z*: $[MH]^+$ calc. for $C_{13}H_{20}N$: 190,1596; found: 190.1589.

*2-phenyl-1,2,3,4-tetrahydroquinoline (**2j**).* The title compound was synthesized according to procedure **B**. 101.0 mg (0.492 mmol) of **1j** was used, 45.5 mg catalyst. The product was purified through column chromatography (silica gel, EtOAc/*n*-heptane 1:50, 5% triethylamine). Appearance: clear oil, 40.1 mg (0.192 mmol, 39% yield). Analytical data: ^1H NMR (300 MHz, $CDCl_3$, 25 °C): δ = 7.44 – 7.26 (m, 5H), 7.06 – 7.01 (m, 2H), 6.70 – 6.65 (td, *J* = 7.4, 1.1 Hz, 1H), 6.58 – 6.54 (m, 1H), 4.48 – 4.44 (dd, *J* = 9.2, 3.3 Hz, 1H), 4.05 (s, 1H), 3.00 – 2.90 (m, 1H), 2.80 – 2.72 (m, 1H), 2.19 – 2.10 (m, 1H), 2.08 – 1.95 (m, 1H) ppm; $^{13}C\{^1H\}$ NMR (75.5 MHz, $CDCl_3$, 25 °C): δ = 144.9, 144.8, 129.4, 128.7, 127.6, 127.0, 126.7, 121.0, 117.3, 114.1, 56.4, 31.1, 26.5 ppm. HRMS (APCI-Orbitrap) *m*/*z*: $[MH]^+$ calc. for $C_{15}H_{16}N$: 210.1283; found: 210.1276.

*methyl 1,2,3,4-tetrahydroquinoline-6-carboxylate (**2m**).* The title compound was synthesized according to procedure **B**. 92.6 mg (0.495 mmol) of **1m** were used, 45.2 mg catalyst. Appearance: white solid, 93.4 mg (0.488 mmol, 99% yield). Analytical data: ^1H NMR (300 MHz, $CDCl_3$, 25 °C): δ = 7.66 – 7.62 (m, 2H), 6.40 – 6.37 (m, 1H), 4.30 (s, 1H), 3.83 (s, 3H), 3.37 – 3.33 (t, *J* = 5.5 Hz, 2H), 2.79 – 2.75 (t, *J* = 6.3 Hz,

2H), 1.97 – 1.89 (m, 2H) ppm; $^{13}C\{^{1}H\}$ NMR (75.5 MHz, $CDCl_3$, 25 °C): δ = 167.6, 148.9, 131.5, 129.3, 120.1, 117.7, 112.8, 51.6, 41.9, 27.0, 21.6 ppm. HRMS (APCI-Orbitrap) *m*/*z*: $[MH]^+$ calc. for $C_{11}H_{14}NO_2$: 192.1025; found: 192.1018.

*6-fluoro-1,2,3,4-tetrahydroquinoline (**2n**).* The title compound was synthesized according to procedure **A**. 74.2 mg (0.504 mmol) of **1n** were used, 45.3 mg catalyst. Appearance: yellow oil, 69.9 mg (0.462 mmol, 92% yield). Analytical data: ^{1}H NMR (300 MHz, $CDCl_3$, 25 °C): δ = 6.72 – 6.67 (m, 2H), 6.43 – 6.38 (m, 1H), 3.65 (s, 1H), 3.29 – 3.25 (m, 2H), 2.77 – 2.73 (t, J = 6.4 Hz, 2H), 1.97 – 1.89 (p, J = 5.5 Hz, 2H) ppm; $^{13}C\{^{1}H\}$ NMR (75.5 MHz, $CDCl_3$, 25 °C): δ = 157.1, 154.0, 141.0, 141.0, 122.9, 122.8, 115.8, 115.5, 115.0, 114.9, 113.4, 113.1, 42.1, 27.1, 27.1, 22.1 ppm. ^{19}F NMR (282 MHz, $CDCl_3$, 25 °C): δ = -128.4 ppm. HRMS (APCI-Orbitrap) *m*/*z*: $[MH]^+$ calc. for $C_9H_{11}FN$: 152.0876; found: 152.0867.

*6-fluoro-2-methyl-1,2,3,4-tetrahydroquinoline (**2o**).* The title compound was synthesized according to procedure **A**. 84.0 mg (0.521 mmol) of **1o** were used, 45.4 mg catalyst. Appearance: white solid, 72.7 mg (0.437 mmol, 84% yield). Analytical data: ^{1}H NMR (300 MHz, $CDCl_3$, 25 °C): δ = 6.70 – 6.63 (m, 2H), 6.42 – 6.37 (m, 1H), 3.57 (s, 1H), 3.40 – 3.30 (m, 1H), 2.88 – 2.65 (m, 2H), 1.96 – 1.88 (m, 1H), 1.63 – 1.49 (m, 1.5H), 1.26 (s, 0.5H), 1.21 – 1.19 (d, J = 6.3 Hz, 3H) ppm; $^{13}C\{^{1}H\}$ NMR (75.5 MHz, $CDCl_3$, 25 °C): δ = 157.2, 154.1, 141.1, 141.1, 122.6, 122.6, 115.7, 115.4, 114.9, 114.8, 113.4, 113.1, 47.4, 30.0, 26.9, 26.8, 22.6 ppm. ^{19}F NMR (282 MHz, $CDCl_3$, 25 °C): δ = -128.3 ppm. HRMS (APCI-Orbitrap) *m*/*z*: $[MH]^+$ calc. for $C_{10}H_{13}FN$: 166.1032; found: 166.1026.

*8-fluoro-1,2,3,4-tetrahydroquinoline (**2p**).* The title compound was synthesized according to procedure **A**. 85.4 mg (0.580 mmol) of **1p** were used, 45.5 mg catalyst. Appearance: clear oil, 69.0 mg (0.456 mmol, 79% yield). Analytical data:^{1}H NMR (300 MHz, $CDCl_3$, 25 °C): δ = 6.86 – 6.76 (m, 2H), 6.57 – 6.50 (m, 1H), 4.02 (s, 1H), 3.38 – 3-34 (m, 2H), 2.83 – 2.79 (t, J =6.4 Hz, 2H), 2.02 – 1.94 (m, 2H) ppm; $^{13}C\{^{1}H\}$ NMR (75.5 MHz, $CDCl_3$, 25 °C): δ = 152.6, 149.4, 133.4, 133.2, 124.6, 124.5, 123.7, 123.6, 115.6, 115.5,

112.3, 112.1, 41.3, 26.7, 26.6, 21.9 ppm. ^{19}F NMR (282 MHz, $CDCl_3$, 25 ˚C): δ = -139.0 ppm. HRMS (APCI-Orbitrap) *m/z*: $[MH]^+$ calc. for $C_9H_{11}FN$: 152.0876; found: 152.0869.

5-chloro-1,2,3,4-tetrahydroquinoline (2q). The title compound was synthesized according to procedure **A**. 77.4 mg (0.473 mmol) of **1q** were used, 45.5 mg catalyst. Appearance: yellow oil, 77.1 mg (0.460 mmol, 97% yield). Analytical data:^{1}H NMR (300 MHz, $CDCl_3$, 25 ˚C): δ = 6.93 – 6.87 (t, *J* = 8.0 Hz, 1H), 6.70 – 6.67 (d, *J* = 7.8 Hz, 1H), 6.38 – 35 (d, *J* = 8.0 Hz, 1H), 3.85 (s, 1H), 3.31 (s, 2H), 2.84 – 2.79 (t, *J* = 6.6 Hz, 2H), 2.01 – 1.93 (m, 2h) ppm; ^{13}C{^{1}H} NMR (75.5 MHz, $CDCl_3$, 25 ˚C): δ = 146.4, 134.8, 127.1, 119.3, 117.5, 112.6, 41.4, 24.7, 22.0 ppm. HRMS (APCI-Orbitrap) *m/z*: $[MH]^+$ calc. for $C_9H_{11}ClN$: 168.0580; found: 168.0574.

6-chloro-1,2,3,4-tetrahydroquinoline (2r). The title compound was synthesized according to procedure **B**. 80.4 mg (0.491 mmol) of **1r** were used, 45.2 mg catalyst. Appearance: yellow oil, 71.3 mg (0.425 mmol, 87% yield). Analytical data: ^{1}H NMR (300 MHz, $CDCl_3$, 25 ˚C): δ = 6.92 – 6.89 (m, 2H), 6.39 – 6.36 (m, 1H), 3.65 (s, 1H), 3.31 (s, 2H), 2.76 – 2.71 (t, *J* = 6.4 Hz, 2H), 1.95 – 1.87 (m, 2H) ppm; ^{13}C{^{1}H} NMR (75.5 MHz, $CDCl_3$, 25 ˚C): δ = 143.3, 129.1, 126.6, 123.0, 121.3, 115.3, 41.9, 27.0, 21.9 ppm. HRMS (APCI-Orbitrap) *m/z*: $[MH]^+$ calc. for $C_9H_{11}ClN$: 168.0580; found: 168.0575.

8-chloro-1,2,3,4-tetrahydroquinoline (2s). The title compound was synthesized according to procedure **A**. 87.4 mg (0.534 mmol) of **1s** were used, 45.4 mg catalyst. Appearance: yellow oil, 71.1 mg (0.424 mmol, 79% yield). Analytical data: ^{1}H NMR (300 MHz, $CDCl_3$, 25 ˚C): δ = 7.09 – 7.06 (m, 1H), 6.88 – 6.85 (m, 1H), 6.54 – 6.49 (t, *J* = 7.7 Hz, 1H), 4.41 (s, 1H), 3.42 – 3.38 (m, 2H), 2.81 – 2.77 (t, *J* = 6.4 Hz, 2H), 1.99 – 1.91 (m, 2H) ppm; ^{13}C{^{1}H} NMR (75.5 MHz, $CDCl_3$, 25 ˚C): δ = 140.8, 127.8, 126.9, 122.7, 118.1, 116.4, 41.9, 27.3, 21.8 ppm. HRMS (APCI-Orbitrap) *m/z*: $[MH]^+$ calc. for $C_9H_{11}ClN$: 168.0580; found: 168.0575.

6-bromo-1,2,3,4-tetrahydroquinoline (2t). The title compound was synthesized according to procedure **B**. 100.5 mg (0.483 mmol) of **1t** were used, 45.6 mg catalyst. The product was purified through column chromatography (silica gel, EtOAc/*n*-heptane 1:10, 5% triethylamine). Appearance: yellow oil, 65.1 mg

(0.307 mmol, 64% yield). Analytical data: ^{1}H NMR (300 MHz, $CDCl_3$, 25 ˚C): δ = 7.04 – 7.01 (m, 2H), 6.35 – 6.32 (d, J = 8.2 Hz, 1H), 3.83, (s, 1H), 3.30 – 3.26 (m, 2H), 2.75 – 2.71 (t, J = 6.4 Hz, 2H), 1.95 – 1.87 (m, 2H) ppm; ^{13}C{^{1}H} NMR (75.5 MHz, $CDCl_3$, 25 ˚C): δ = 143.9, 132.0, 129.5, 123.5, 115.6, 108.3, 41.9, 27.0, 21.8 ppm. HRMS (APCI-Orbitrap) *m/z*: $[MH]^+$ calc. for $C_9H_{11}BrN$: 214.0054 (corresponds to ^{81}Br); found: 214.0047.

*6-bromo-2-methyl-1,2,3,4-tetrahydroquinoline (**2u**).* The title compound was synthesized according to procedure **B**. 109.7 mg (0.494 mmol) of **1u** were used, 45.1 mg catalyst. The product was purified through column chromatography (silica gel, EtOAc/*n*-heptane 1:10, 5% triethylamine). Appearance: white solid, 29.8 mg (0.132 mmol, 27% yield). Analytical data: ^{1}H NMR (300 MHz, $CDCl_3$, 25 ˚C): δ = 7.07 – 7.01 (m, 2H), 6.35 – 6.32 (d, J = 8.4 Hz, 1H), 3.71 (s, 1H), 3.43 – 3.32 (m, 1H), 2.86 – 2.65 (m, 2H), 1.96 – 1.87 (m, 1H), 1.61 – 1.48 (m, 1H), 1.22 – 1.19 (d, J = 6.3 Hz, 3H) ppm; ^{13}C{^{1}H} NMR (75.5 MHz, $CDCl_3$, 25 ˚C): δ = 143.9, 131.8, 129.4, 123.2, 115.5, 108.4, 47.2, 29.7, 26.5, 22.6 ppm. HRMS (APCI-Orbitrap) *m/z*: $[MH]^+$ calc. for $C_{10}H_{13}BrN$: 226.0231; found: 226.0230.

*8-bromo-1,2,3,4-tetrahydroquinoline (**2v**).* The title compound was synthesized according to procedure **B**. 105.6 mg (0.508 mmol) of **1v** were used, 45.5 mg catalyst. The product was purified through column chromatography (silica gel, EtOAc/*n*-heptane 1:10, 5% triethylamine). Appearance: clear oil, 19.8 mg (0.093 mmol, 18% yield). Analytical data: ^{1}H NMR (300 MHz, $CDCl_3$, 25 ˚C): δ = 7.24 – 7.21 (m, 1H), 6.90 – 6.87 (m, 1H), 6.48 – 6.43 (t, J = 7.7 Hz, 1H), 4.43 (s, 1H), 3.42 – 3.37 (m, 2H), 2.80 – 2.76 (t, J = 6.4 Hz, 2H), 1.97 – 1.89 (m, 2H) ppm; ^{13}C{^{1}H} NMR (75.5 MHz, $CDCl_3$, 25 ˚C): δ = 141.8, 130.1, 128.5, 123.0, 117.0, 108.8, 42.2, 27.6, 21.8 ppm. HRMS (APCI-Orbitrap) *m/z*: $[MH]^+$ calc. for $C_9H_{11}BrN$: 214.0054 (corresponds to ^{81}Br); found: 214.0047.

*5-methoxy-1,2,3,4-tetrahydroquinoline (**2w**).* The title compound was synthesized according to procedure **A**. 76.6 mg (0.482 mmol) of **1w** were used, 45.1 mg catalyst. Appearance: clear oil, 74.1 mg (0.454 mmol, 94% yield). Analytical data: ^{1}H NMR (300 MHz, $CDCl_3$, 25 ˚C): δ = 6.97 – 6.92 (t, J = 8.1 Hz, 1H), 6.24 – 6.21 (d, J = 8.1 Hz, 1H), 6.18 – 6.16 (d, J = 8.1 Hz, 1H), 3.83 – 3.80 (m, 4H), 3.28 – 3.24 (m, 2H), 2.69

– 2.65 (t, $J = 6.6$ Hz, 2H), 1.98 – 1.90 (m, 2H) ppm; $^{13}C\{^1H\}$ NMR (75.5 MHz, $CDCl_3$, 25 ˚C): $\delta = 158.1$, 146.0, 126.7, 109.9, 107.8, 99.2, 55.4, 41.7, 22.0, 20.7 ppm. HRMS (APCI-Orbitrap) *m/z*: $[MH]^+$ calc. for $C_{10}H_{14}NO$: 164.1075; found: 164.1070.

*6-methoxy-1,2,3,4-tetrahydroquinoline (**2x**).* The title compound was synthesized according to procedure **B**. 82.9 mg (0.521 mmol) of **1x** were used, 45.3 mg catalyst. Appearance: clear oil, 80.7 mg (0.494 mmol, 95% yield). Analytical data: ^1H NMR (300 MHz, $CDCl_3$, 25 ˚C): $\delta = 6.63$ – 6.58 (m, 2H), 6.47 – 6.45 (d, $J = 8.4$ Hz, 1H), 3.74 (s, 3H), 3.54 (s, 1H), 3.28 – 3.24 (m, 2H), 2.79 – 2.75 (t, $J = 6.5$ Hz, 2H), 1.98 – 1.90 (m, 2H) ppm; $^{13}C\{^1H\}$ NMR (75.5 MHz, $CDCl_3$, 25 ˚C): $\delta = 151.9$, 139.0, 122.9, 115.6, 114.9, 113.0, 55.9, 42.4, 27.3, 22.5 ppm. HRMS (APCI-Orbitrap) *m/z*: $[MH]^+$ calc. for $C_{10}H_{14}NO$: 164.1075; found: 164.1068.

*6-methoxy-2-methyl-1,2,3,4-tetrahydroquinoline (**2y**).* The title compound was synthesized according to procedure **B**. 86.5 mg (0.499 mmol) of **1y** were used, 45.5 mg catalyst. The product was purified through column chromatography (silica gel, EtOAc/*n*-heptane 2:1, 5% triethylamine). Appearance: orange oil, 54.2 mg (0.306 mmol, 61% yield). Analytical data: ^1H NMR (300 MHz, $CDCl_3$, 25 ˚C): $\delta = 6.62$ – 6-58 (m, 2H), 6.47 – 6.44 (m, 1H), 3.73 (s, 3H), 3.39 – 3.28 (m, 2H), 2.91 – 2.80 (m, 1H), 2.75 – 2.67 (m, 1H), 1.96 – 1.88 (m, 1H), 1.65 – 1.51 (m, 1H), 1.22 – 1.20 (d, $J = 6.3$ Hz, 3H) ppm; $^{13}C\{^1H\}$ NMR (75.5 MHz, $CDCl_3$, 25 ˚C): $\delta = 152.0$, 139.0, 122.6, 115.4, 114.8, 113.0, 55.9, 47.6, 30.4, 27.0, 22.7 ppm. HRMS (APCI-Orbitrap) *m/z*: $[MH]^+$ calc. for $C_{11}H_{16}NO$: 178.1232; found: 178.1227.

*1,2,3,4-tetrahydroquinolin-6-amine (**2z**).* The title compound was synthesized according to procedure **C**. 71.7 mg (0.497 mmol) of **1z** were used, 90.3 mg catalyst. Appearance: white solid, 83.9 mg (0.456 mmol, 92% yield). Analytical data: ^1H NMR (300 MHz, D_2O, 25 ˚C): $\delta = 7.48$ – 7.36 (m, 3H), 3.59 – 3.56 (m, 2H), 3.01 – 2.96 (t, $J = 6.5$ Hz, 2H), 2.19 – 2.11 (m, 2H) ppm; $^{13}C\{^1H\}$ NMR (75.5 MHz, D_2O, 25 ˚C): $\delta = 134.4$, 130.7, 130.1, 125.4, 124.9, 122.1, 42.5, 24.4, 18.6 ppm. HRMS (APCI-Orbitrap) *m/z*: $[MH]^+$ calc. for $C_9H_{13}N_2$: 149.1079; found: 149.1072.

*1,2,3,4-tetrahydro-1,5-naphthyridine (**2ab**).* The title compound was synthesized according to procedure A. 65.5 mg (0.503 mmol) of **1ab** were used, 45.1 mg catalyst. Appearance: white solid, 62.3 mg (0.464 mmol,

92% yield). Analytical data: ^{1}H NMR (300 MHz, $CDCl_3$, 25 °C): δ = 7.84 – 7.82 (dd, J = 4.7, 1.4 Hz, 1H), 6.88 – 6.84 (m, 1H), 6.72 – 6.68 (m, 1H), 3.84 (s, 1H), 3.29 – 3.26 (m, 2H), 2.93 – 2.89 (t, *J* = 6.5 Hz, 2H), 3.05 – 1.97 (m, 2H) ppm; ^{13}C{^{1}H} NMR (75.5 MHz, $CDCl_3$, 25 °C): δ = 142.9, 141.0, 138.1, 122.0, 120.3, 41.6, 30.5, 21.9 ppm. HRMS (APCI-Orbitrap) *m*/*z*: $[MH]^+$ calc. for $C_8H_{11}N_2$: 135.0922; found: 135.0915.

phenylmethanol (4a). The title compound was synthesized according to procedure **B**. 50.7 mg (0.478 mmol) of **3a** were used, 45.2 mg catalyst. The product was purified through column chromatography (silica gel, EtOAc/*n*-heptane 1:1,). Appearance: clear oil, 14.1 mg (0.130 mmol, 27% yield). Analytical data:^{1}H NMR (300 MHz, $CDCl_3$, 25 °C): δ = 7.40 – 7.28 (m, 5H), 4.68 (s, 2H), 1.72 (s, 1H) ppm; ^{13}C{^{1}H} NMR (75.5 MHz, $CDCl_3$, 25 °C): δ = 141.0, 128.7, 127.8, 127.1, 65.5 ppm. HRMS (APCI-Orbitrap) *m*/*z*: $[MH – H_2O]^+$ calc. for $C_7H_9^+$: 91.0542; found: 91.0542.

o-tolylmethanol (4b). The title compound was synthesized according to procedure **B**. 57.3 mg (0.477 mmol) of **3b** were used, 45.0 mg catalyst. The product was purified through column chromatography (silica gel, EtOAc/*n*-heptane 1:1,). Appearance: white solid, 36.4 mg (0.298 mmol, 62% yield). Analytical data: ^{1}H NMR (300 MHz, $CDCl_3$, 25 °C): δ = 7.28 – 7.19 (m, 4H), 4.63 (s, 2H), 2.39 (s, 3H), 2.31 (s, 1H) ppm; ^{13}C{^{1}H} NMR (75.5 MHz, $CDCl_3$, 25 °C): δ = 138.0, 137.3, 129.3, 127.2, 65.1, 21.2 ppm. HRMS (APCI-Orbitrap) *m*/*z*: $[MH – H_2O]^+$ calc. for $C_8H_9^+$: 105.0699; found: 105.0699.

m-tolylmethanol (4c). The title compound was synthesized according to procedure **B**. 56.6 mg (0.471 mmol) of **3c** were used, 45.0 mg catalyst. The product was purified through column chromatography (silica gel, EtOAc/*n*-heptane 1:1,). Appearance: clear liquid, 40.2 mg (0.329 mmol, 70% yield). Analytical data: ^{1}H NMR (300 MHz, $CDCl_3$, 25 °C): δ = 7.29 – 7.11 (m, 4H), 4.64 (s, 2H), 2.37 (s, 3H), 1.94 (s, 1H) ppm; ^{13}C{^{1}H} NMR (75.5 MHz, $CDCl_3$, 25 °C): δ = 140.9, 138.3, 128.6, 128.5, 127.9, 124.1, 65.4, 21.5 ppm. HRMS (APCI-Orbitrap) *m*/*z*: $[MH – H_2O]^+$ calc. for $C_8H_9^+$: 105.0699; found: 105.0699.

p-tolylmethanol (4d). The title compound was synthesized according to procedure **B**. 60.1 mg (0.500 mmol) of **3d** were used, 45.2 mg catalyst. The product was purified by column chromatography (silica gel, EtOAc/*n*-heptane 1:1). Appearance: white solid, 41.6 mg (0.341 mmol, 68% yield). Analytical data: ^{1}H NMR (300 MHz, $CDCl_3$, 25 °C): δ = 7.37 – 7.34 (m, 1H), 7.24 – 7.17 (m, 3H), 4.66 (s, 2), 2.36 (s, 3H), 2.10 (s, 1H) ppm; ^{13}C{^{1}H} NMR (75.5 MHz, $CDCl_3$, 25 °C): δ = 138.8, 136.1, 130.4, 127.8, 127.6, 126.1, 63.4, 18.7 ppm. HRMS (APCI-Orbitrap) *m/z*: $[MH – H_2O]^+$ calc. for $C_8H_9^+$: 105.0699; found: 105.0706.

(4-(tert-butyl)phenyl)methanol (4e). The title compound was synthesized according to procedure **B**. 87.7 mg (0.541 mmol) of **3e** were used, 45.6 mg catalyst. The product was purified through column chromatography (silica gel, EtOAc/*n*-heptane 1:1). Appearance: clear liquid, 37.6 mg (0.229 mmol, 42% yield). Analytical data: ^{1}H NMR (300 MHz, $CDCl_3$, 25 °C): δ = 7.42 – 7.39 (m, 2H), 7.32 – 7.29 (m, 2H), 4.65 (s, 2H), 2.04 (s, 1H), 1.35 (s, 9H) ppm; ^{13}C{^{1}H} NMR (75.5 MHz, $CDCl_3$, 25 °C): δ = 150.8, 138.0, 127.0, 125.6, 65.2, 34.6, 31.5 ppm. HRMS (APCI-Orbitrap) *m/z*: $[MH – H_2O]^+$ calc. for $C_{11}H_{15}^+$: 147.1168; found: 147.1170.

methyl 4-(hydroxymethyl)benzoate (4f). The title compound was synthesized according to procedure **B**. 80.5 mg (0.490 mmol) of **3f** were used, 45.6 mg catalyst. Appearance: white solid, 80.3 mg (0.483 mmol, 99% yield). Analytical data: ^{1}H NMR (300 MHz, $CDCl_3$, 25 °C): δ = 7.96 – 7.93 (d, *J* = 8.0 Hz, 2H), l7.37 – 7.35 (d, *J* = 7.9 Hz, 2H), 4.68 (s, 2H), 3.86 (s, 3H), 2.94 (s, 1H) ppm; ^{13}C{^{1}H} NMR (75.5 MHz, $CDCl_3$, 25 °C): δ = 167.2, 146.3, 129.8, 129.1, 126.5, 64.5, 52.2 ppm. HRMS (APCI-Orbitrap) *m/z*: $[MH]^+$ calc. for $C_9H_{11}O_3$: 167.0708; found: 167.0704.

2-(hydroxymethyl)-6-methoxyphenol (4g). The title compound was synthesized according to procedure **B**. 83.3 mg (0.547 mmol) of **3g** were used, 45.3 mg catalyst. The product was purified through column chromatography (silica gel, EtOAc/*n*-heptane 1:1). Appearance: white solid, 25.4 mg (0.165 mmol, 30% yield). Analytical data: ^{1}H NMR (300 MHz, $CDCl_3$, 25 °C): δ = 6.86 – 6.82 (m, 3H), 6.15 (s, 1H), 4.74 (s, 2H), 3.89 (s, 3H). 2.43 (s, 1H) ppm; ^{13}C{^{1}H} NMR (75.5 MHz, $CDCl_3$, 25 °C): δ = 146.7, 143.9, 126.6,

121.0, 119.9, 110.6, 61.9, 56.2 ppm. HRMS (APCI-Orbitrap) *m/z*: $[MH - H_2O]^+$ calc. for $C_8H_9O_2^+$: 137.0597; found: 137.0599.

(2,5-dimethoxyphenyl)methanol (4h). The title compound was synthesized according to procedure **B**. 83.7 mg (0.504 mmol) of **3h** were used, 45.6 mg catalyst. Appearance: clear liquid, 70.5 mg (0.419 mmol, 83% yield). Analytical data: ^{1}H NMR (300 MHz, $CDCl_3$, 25 °C): δ = 6.89 – 6.88 (d, J = 2.1 Hz, 1H), 6.79 – 6.78 (m, 2H), 4.66 – 4.64 (d, J=6.0 Hz, 2H), 3.81 (s, 3H), 3.77 (s, 3H), 2.49 – 2.45 (m, 1H) ppm; ^{13}C{^{1}H} NMR (75.5 MHz, $CDCl_3$, 25 °C): δ = 153.7, 151.6, 130.2, 114.9, 113.1, 111.2, 62.1, 55.9 ppm. HRMS (APCI-Orbitrap) *m/z*: $[MH - H_2O]^+$ calc. for $C_9H_{11}O_2^+$: 151.0754; found: 151.0756.

(2,6-dimethoxyphenyl)methanol (4i). The title compound was synthesized according to procedure **B**. 85.6 mg (0.515 mmol) of **3i** were used, 45.2 mg catalyst. Appearance: white solid, 73.3 mg (0.436 mmol, 85% yield). Analytical data: ^{1}H NMR (300 MHz, $CDCl_3$, 25 °C): δ = 7.24 – 7.19 (t, J = 8.4 Hz, 1H), 6.58 – 6.55 (d, J = 8.4 Hz, 2H), 4.80 – 4.79 (d, J = 6.4 Hz, 2H), 3.84 (s, 6H), 2.50 – 2.45 (t, J = 6.5 Hz, 1H) ppm; ^{13}C{^{1}H} NMR (75.5 MHz, $CDCl_3$, 25 °C): δ = 158.5, 129.3, 117.1, 103.9 55.9, 54.8 ppm. HRMS (APCI-Orbitrap) *m/z*: $[MH - H_2O]^+$ calc. for $C_9H_{11}O_2^+$: 151.0754; found: 151.0756.

naphthalen-2-ylmethanol (4j). The title compound was synthesized according to procedure **B**. 90.6 mg (0.580 mmol) of **3j** were used, 45.5 mg catalyst. Appearance: white solid, 77.7 mg (0.491 mmol, 85% yield). Analytical data: ^{1}H NMR (300 MHz, $CDCl_3$, 25 °C): δ = 8.15 – 8.12 (m, 1H), 7.90 – 7.81 (m, 2H), 7.59 – 7.43 (m, 4H), 5.16 – 5.15 (d, J = 3.8 Hz, 2H), 1.81 (s, 1H) ppm; ^{13}C{^{1}H} NMR (75.5 MHz, $CDCl_3$, 25 °C): δ = 136.4, 133.9, 131.4, 128.8, 128.7, 126.5, 125.5, 125.5, 123.8, 63.9 ppm. HRMS (APCI-Orbitrap) *m/z*: $[MH - H_2O]^+$ calc. for $C_{11}H_9^+$: 141.0699; found: 141.0701.

(5-bromopyridin-3-yl)methanol (4k). The title compound was synthesized according to procedure **B**. 90.9 mg (0.489 mmol) of **3k** were used, 45.1 mg catalyst. Appearance: white solid, 30.0 mg (0.160 mmol, 33% yield). Analytical data: ^{1}H NMR (300 MHz, $CDCl_3$, 25 °C): δ = 8.57 (s, 1H), 8.47 (s, 1H), 7.89 (s,

1H), 4.72 (s, 2H), 2.71 (s, 1H) ppm; $^{13}C\{^1H\}$ NMR (75.5 MHz, $CDCl_3$, 25 °C): δ = 150.0, 146.4, 138.3, 137.5, 121.1, 61.9 ppm. HRMS (APCI-Orbitrap) *m/z*: $[MH]^+$ calc. for C_6H_7BrNO: 187.9711; found: 187.9709.

1,2-didodecyldisulfane (5a). The title compound was synthesized according to the general procedure for thiol oxidation. 102.6 mg (0.507 mmol) of **6a** were used, 51.0 mg catalyst. Appearance: white solid, 101.1 mg (0.251 mmol, 99% yield). Analytical data: ^1H NMR (300 MHz, $CDCl_3$, 25 °C): δ = 2.70 – 2.66 (t, *J* = 7.3 Hz, 2H), 1.72 – 1.62 (m, 2H), 1.40 – 1.26 (m, 18H), 0.90 – 0.86 (m, 3H) ppm; $^{13}C\{^1H\}$ NMR (75.5 MHz, $CDCl_3$, 25 °C): δ = 39.4, 32.1, 29.8, 29.8, 29.8, 29.7, 29.5, 29.4, 28.7, 22.8, 14.3 ppm. HRMS (APCI-Orbitrap) *m/z*: The molecule was not ionizable.

1,2-diphenyldisulfane (5b). The title compound was synthesized according to the general procedure for thiol oxidation. 60.0 mg (0.545 mmol) of **6a** were used, 25.5 mg catalyst. Appearance: white solid, 57.7 mg (0.264 mmol, 97% yield). Analytical data: ^1H NMR (300 MHz, $CDCl_3$, 25 °C): δ = 7.52 – 7.48 (m, 4H), 7.33 – 7.27 (m, 4H), 7.25 – 7.20 (m, 2H) ppm; $^{13}C\{^1H\}$ NMR (75.5 MHz, $CDCl_3$, 25 °C): δ = 137.2, 129.2, 127.7, 127.3 ppm. HRMS (APCI-Orbitrap) *m/z*: $[MH]^+$ calc. for $C_{12}H_{10}S_2$: 218.0224; found: 218.0221.

1,2-bis(4-nitrophenyl)disulfane (5c). The title compound was synthesized according to the general procedure for dehydrogenation. 76.9 mg (0.496 mmol) of **6c** were used, 52.3 mg catalyst. Appearance: off-white solid, 69.3 mg (0.225 mmol, 91% yield). Analytical data:^1H NMR (300 MHz, $CDCl_3$, 25 °C): δ = 8.22 – 8.17 (m, 4H), 7.64 – 7.59 (m, 4H) ppm; $^{13}C\{^1H\}$ NMR (75.5 MHz, $CDCl_3$, 25 °C): δ = 147.1, 144.2, 126.5, 124.6 ppm. HRMS (APCI-Orbitrap) *m/z*: $[MH]^+$ calc. for $C_{12}H_8N_2O_4S_2$: 307.9925; found: 307.9923.

Spectra

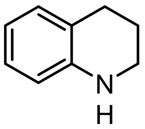

2a

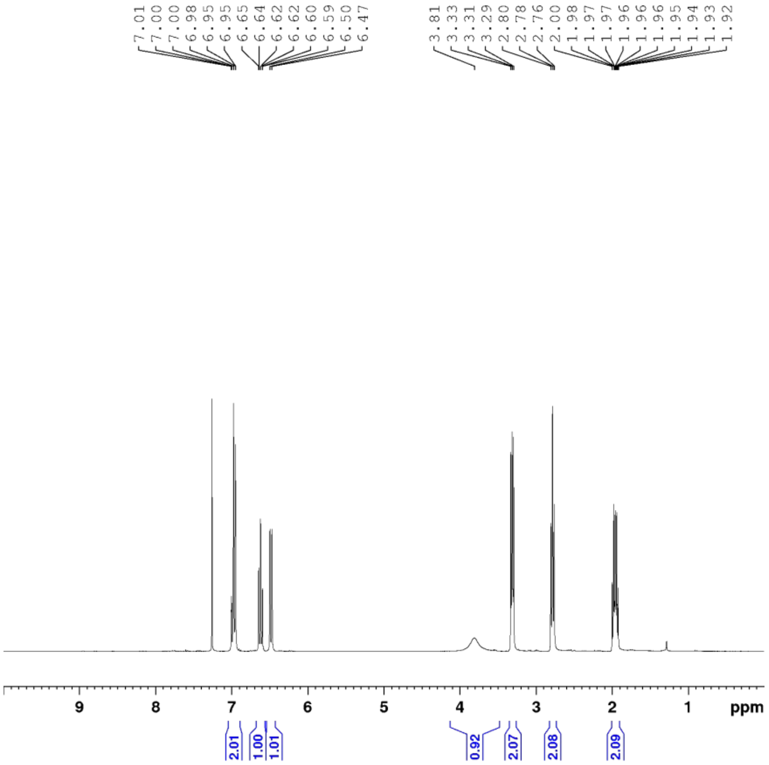

Figure S13. ^{1}H NMR spectrum of **2a** in $CDCl_3$.

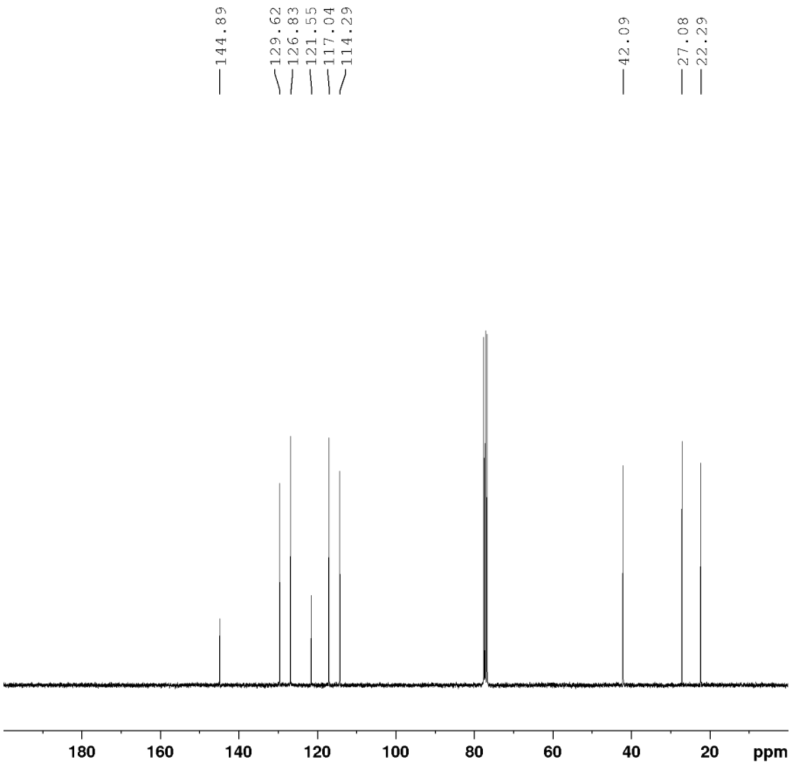

Figure S14. $^{13}C\{^{1}H\}$ NMR spectrum of **2a** in $CDCl_3$.

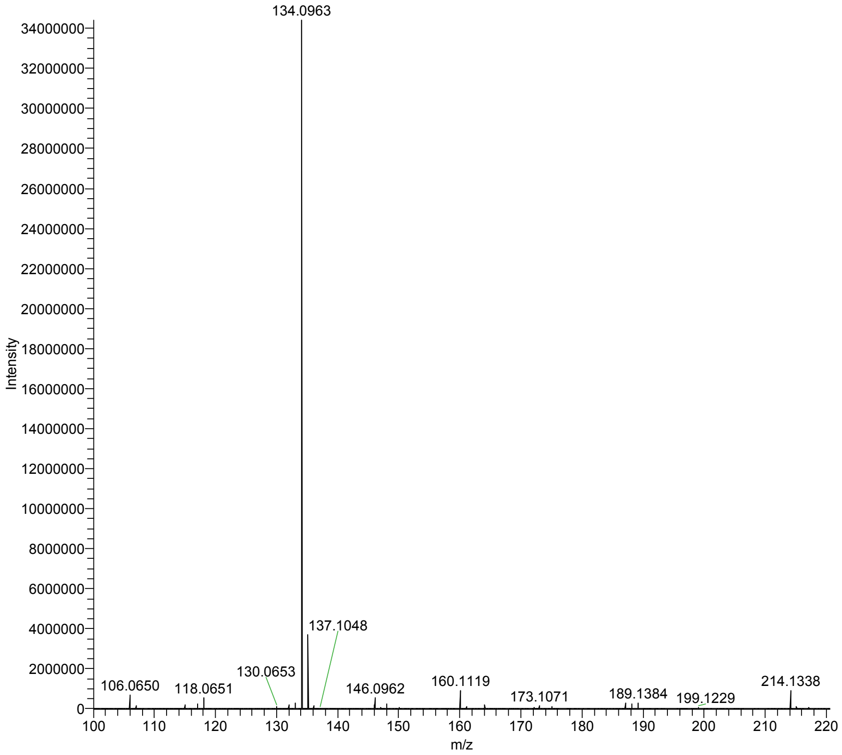

Figure S15. HRMS of **2a**.

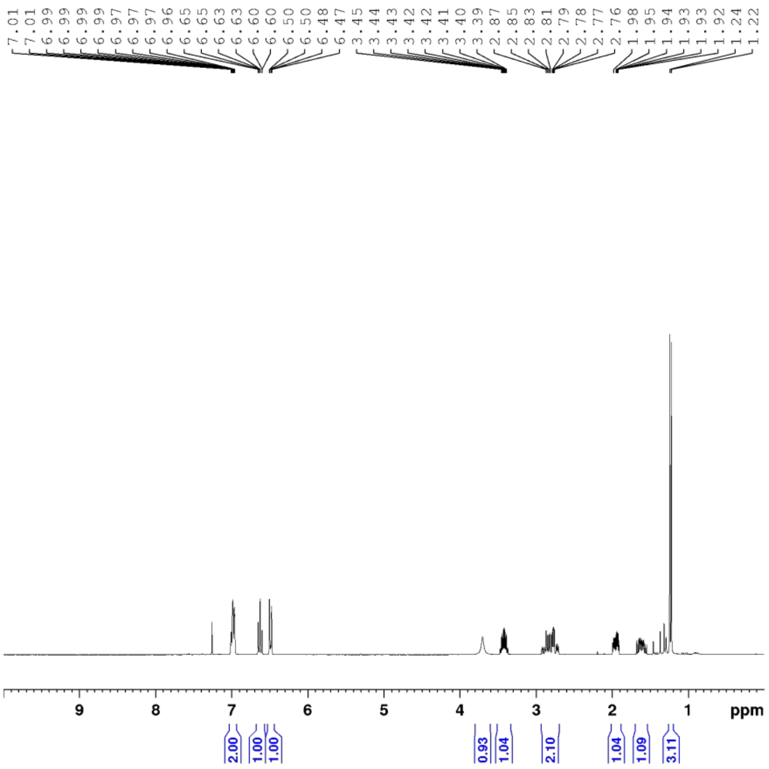

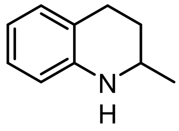

2b

Figure S16. ^{1}H NMR spectrum of **2b** in $CDCl_3$.

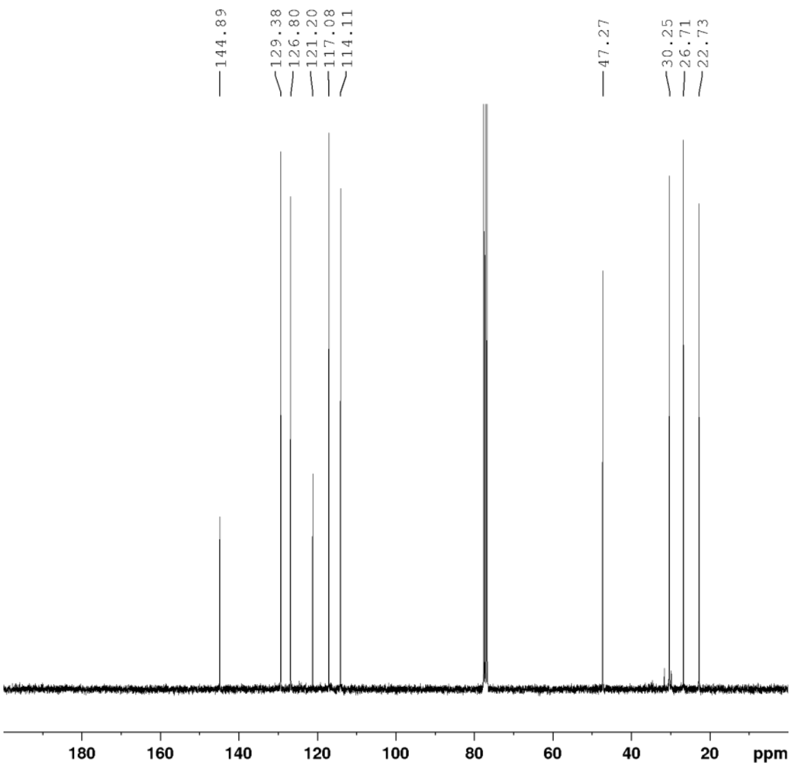

Figure S17. $^{13}C\{^{1}H\}$ NMR spectrum of **2b** in $CDCl_3$.

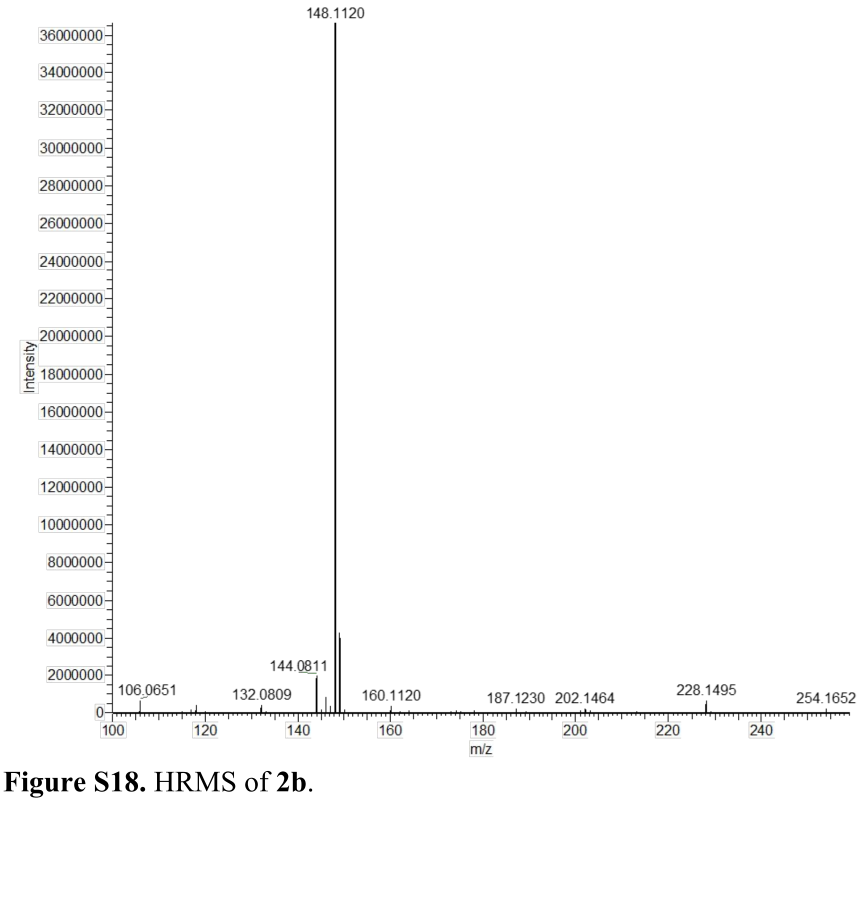

Figure S18. HRMS of **2b**.

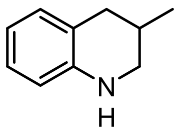

2c

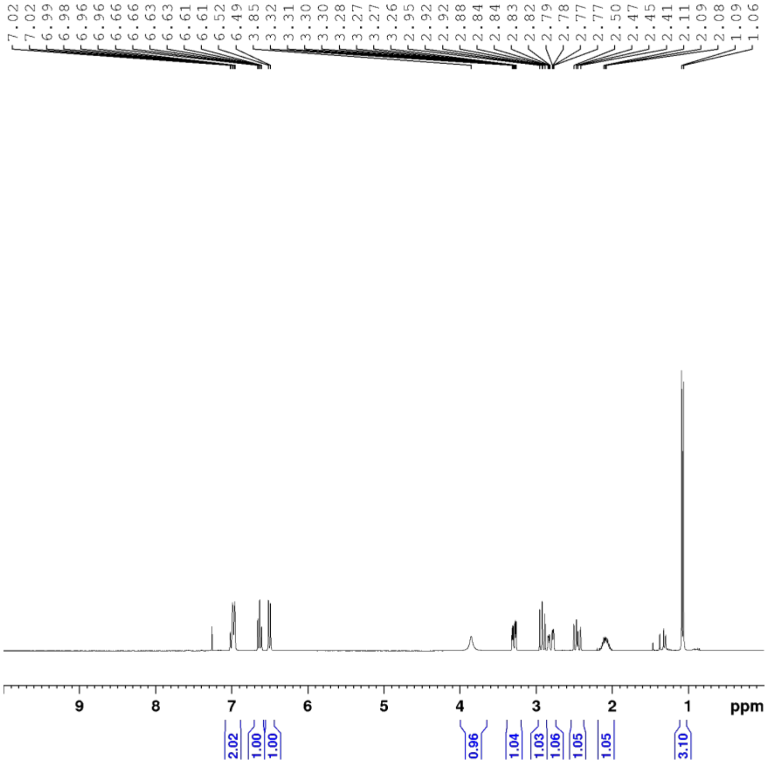

Figure S19. ^{1}H NMR spectrum of **2c** in $CDCl_3$.

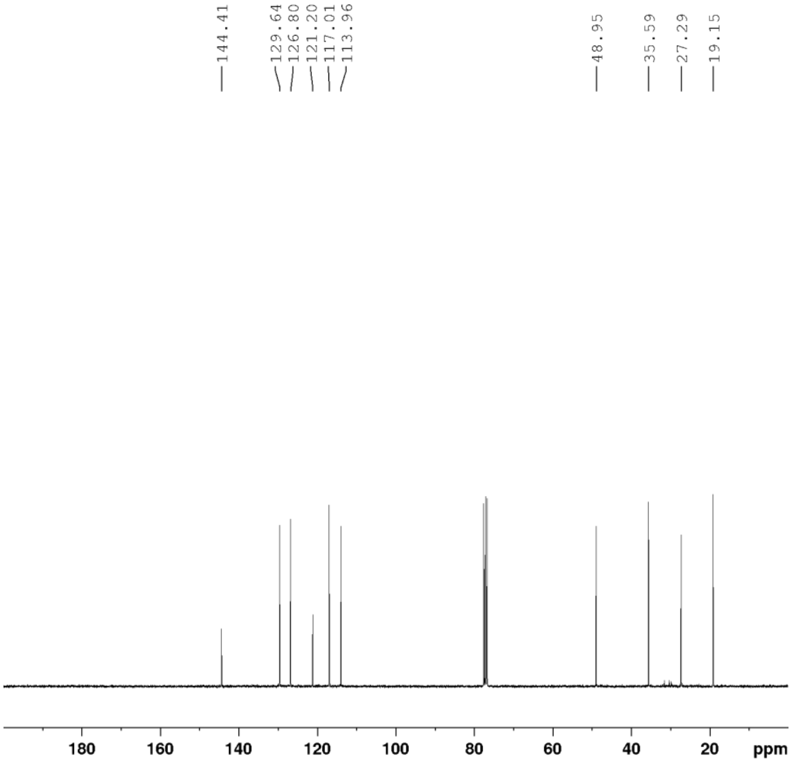

Figure S20. $^{13}C\{^{1}H\}$ NMR spectrum of **2c** in $CDCl_3$.

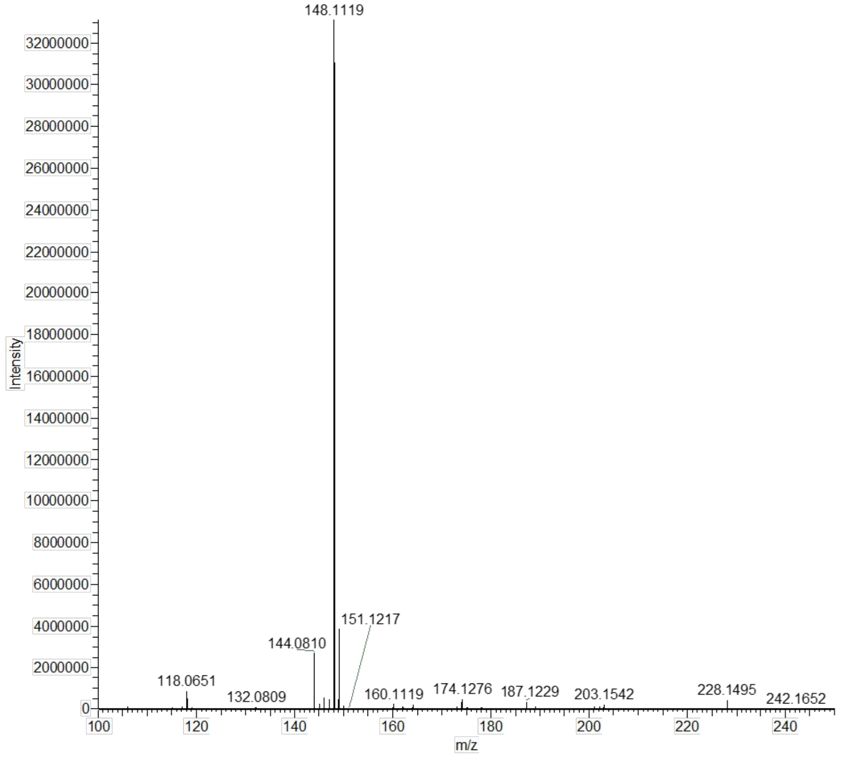

Figure S21. HRMS of **2c**.

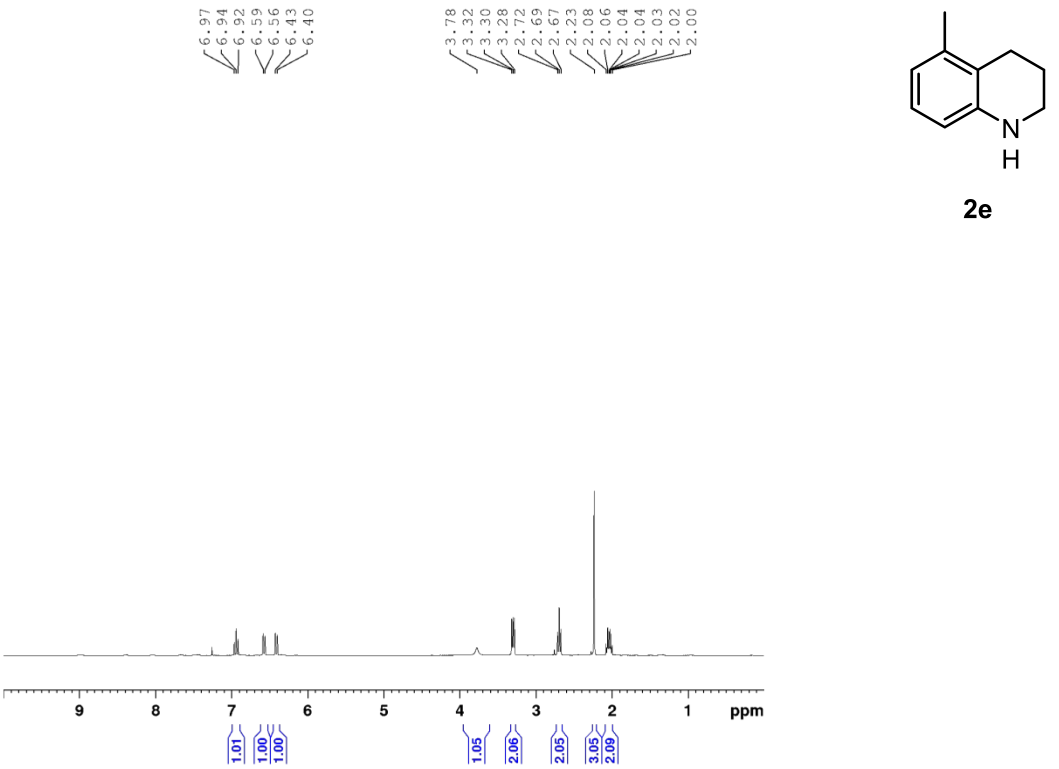

Figure S22. ^{1}H NMR spectrum of **2e** in $CDCl_3$.

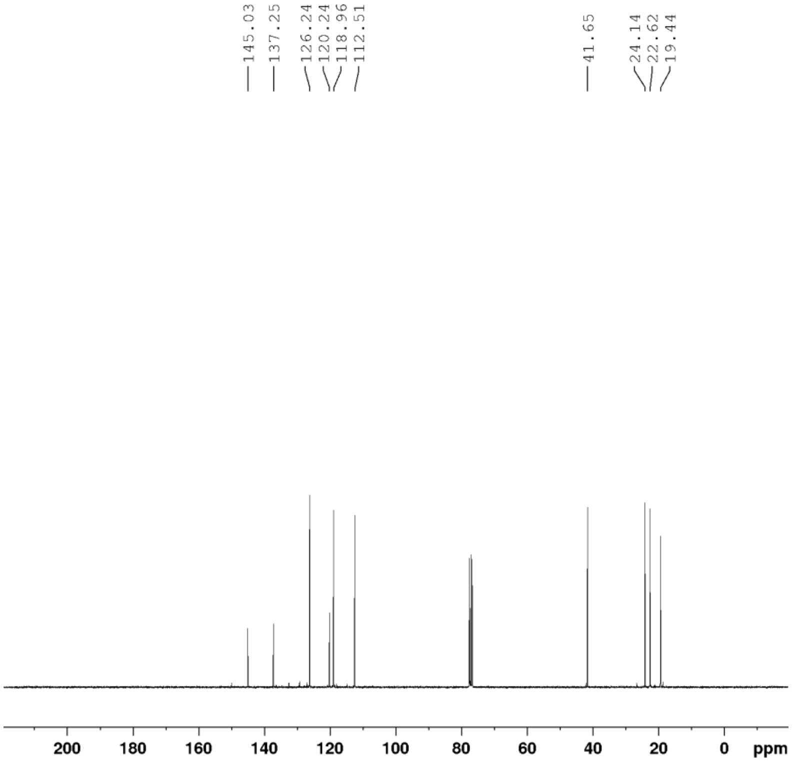

Figure S23. $^{13}C\{^{1}H\}$ NMR spectrum of **2e** in $CDCl_3$.

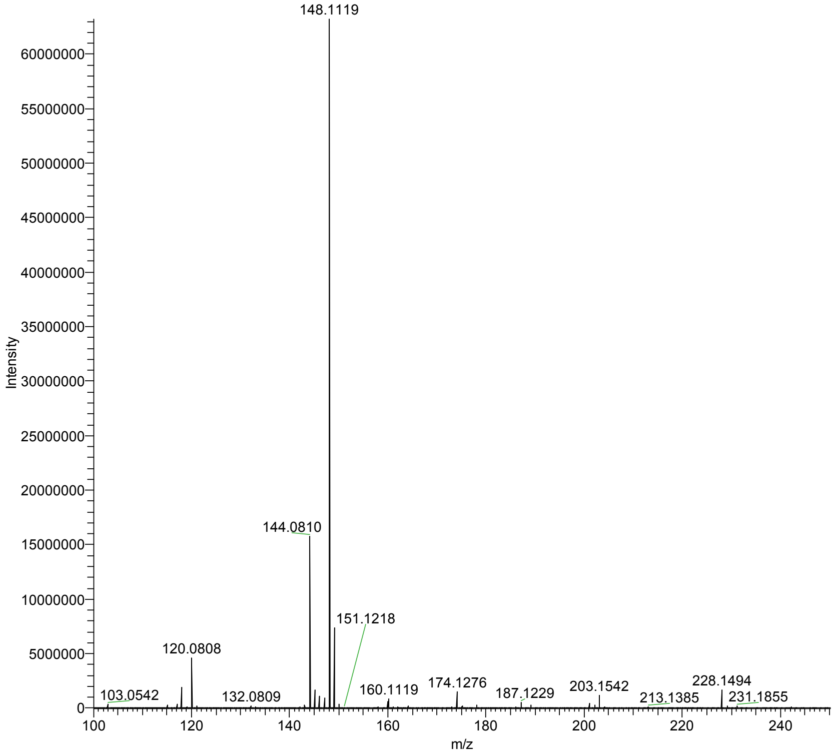

Figure S24. HRMS of **2e**.

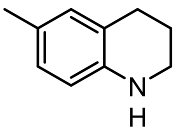

2f

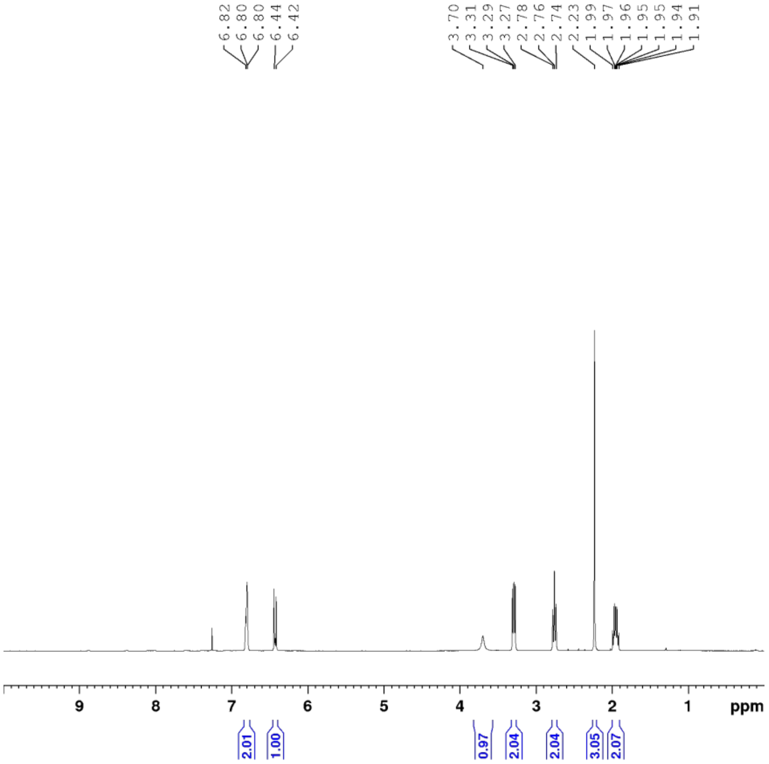

Figure S25. ^{1}H NMR spectrum of **2f** in $CDCl_3$.

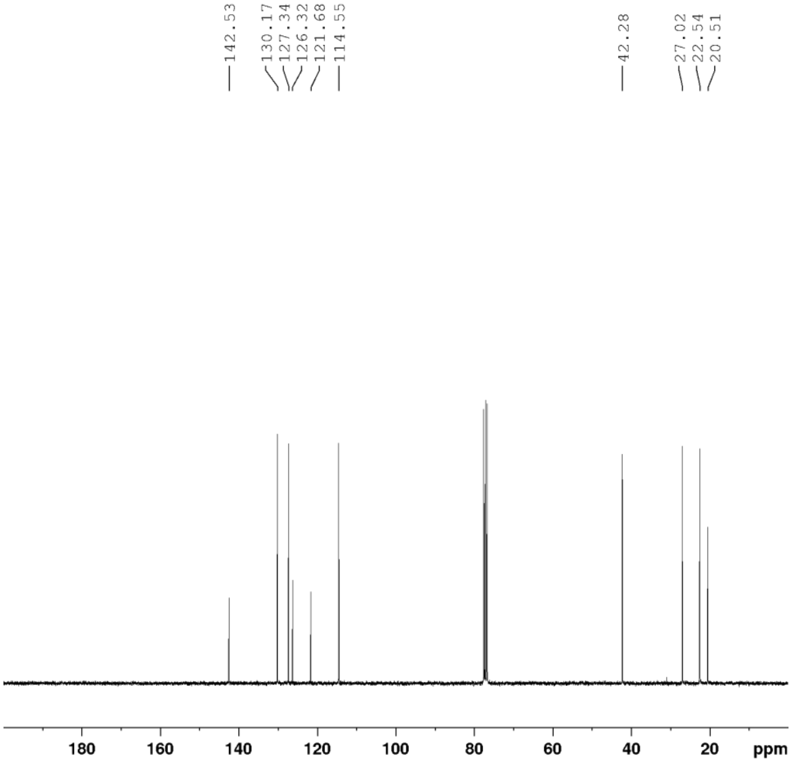

Figure S26. $^{13}C\{^{1}H\}$ NMR spectrum of **2f** in $CDCl_3$.

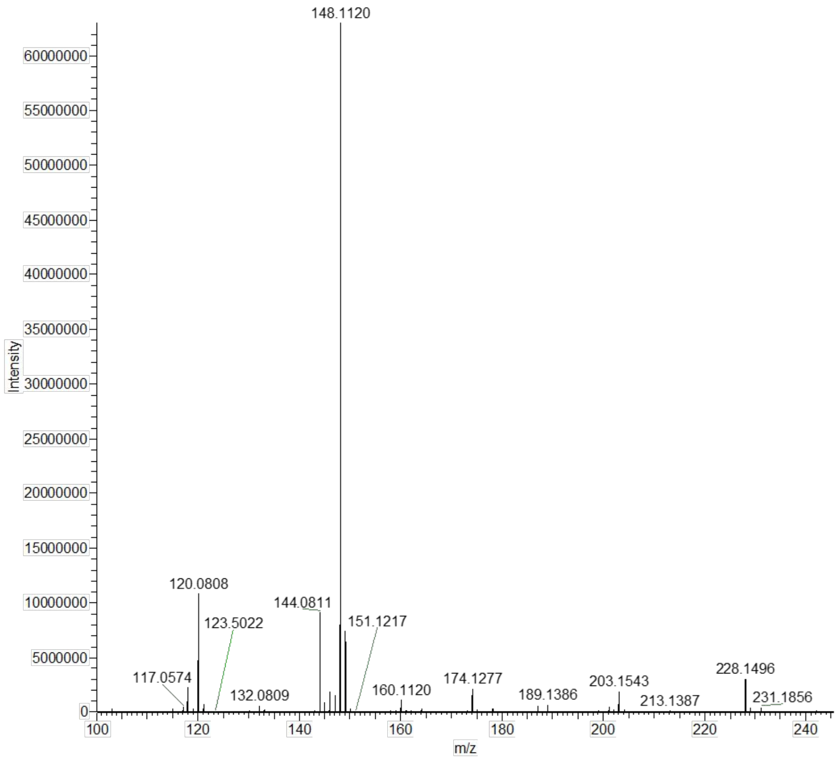

Figure S27. HRMS of **2f**.

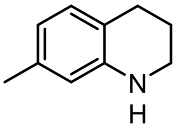

2g

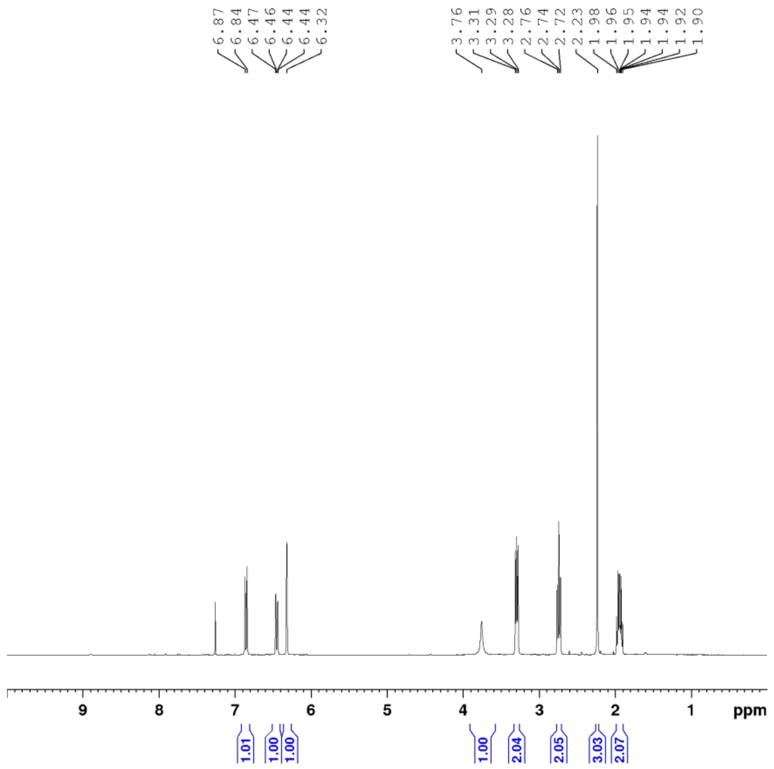

Figure S28. ^{1}H NMR spectrum of **2g** in $CDCl_3$.

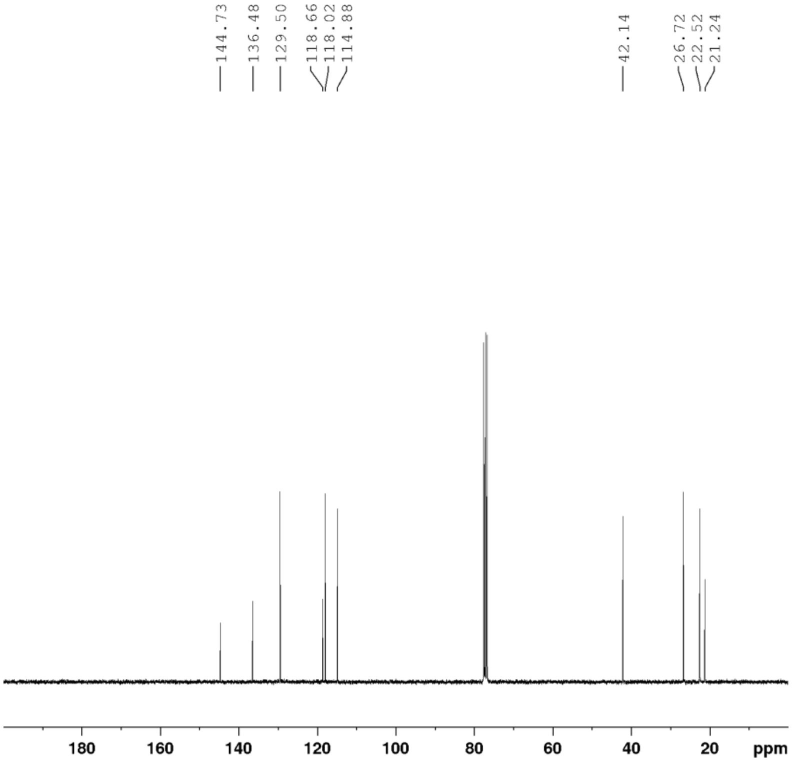

Figure S29. $^{13}C\{^{1}H\}$ NMR spectrum of **2g** in $CDCl_3$.

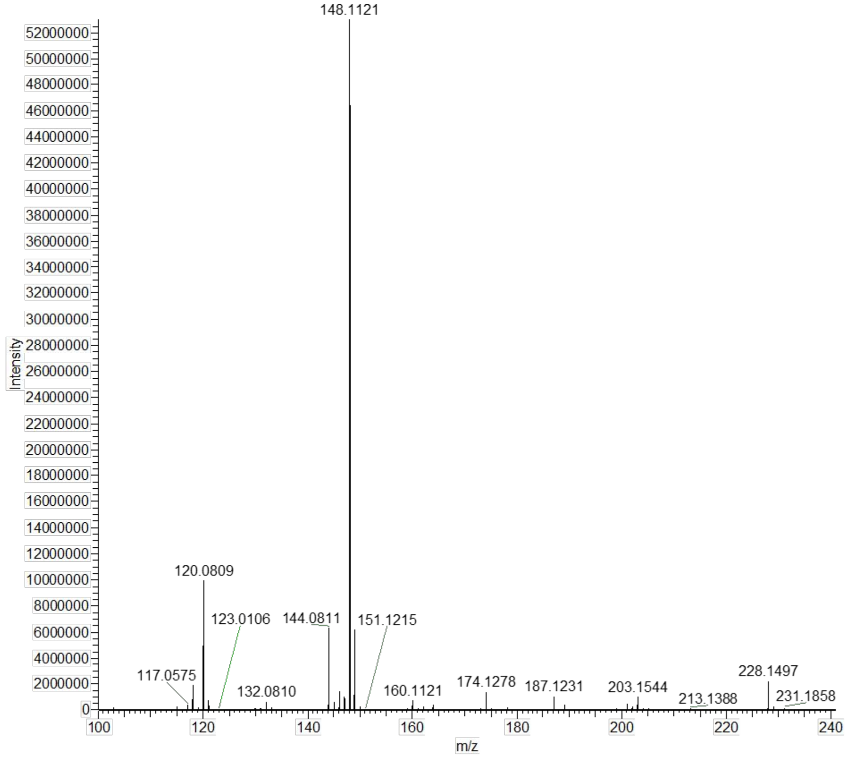

Figure S30. HRMS of **2g**.

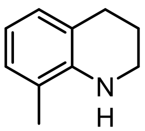

2h

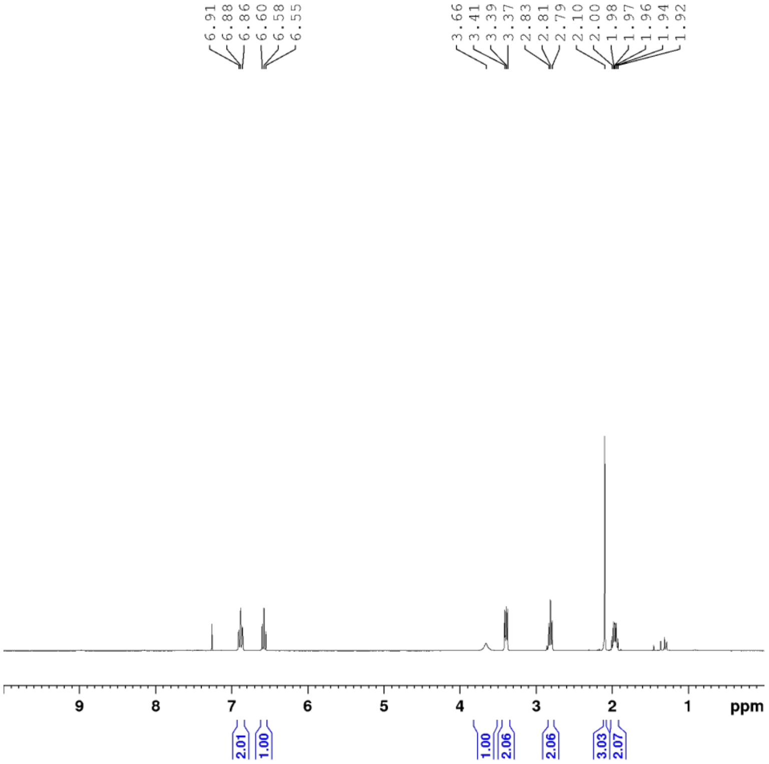

Figure S31. ^{1}H NMR spectrum of **2h** in $CDCl_3$.

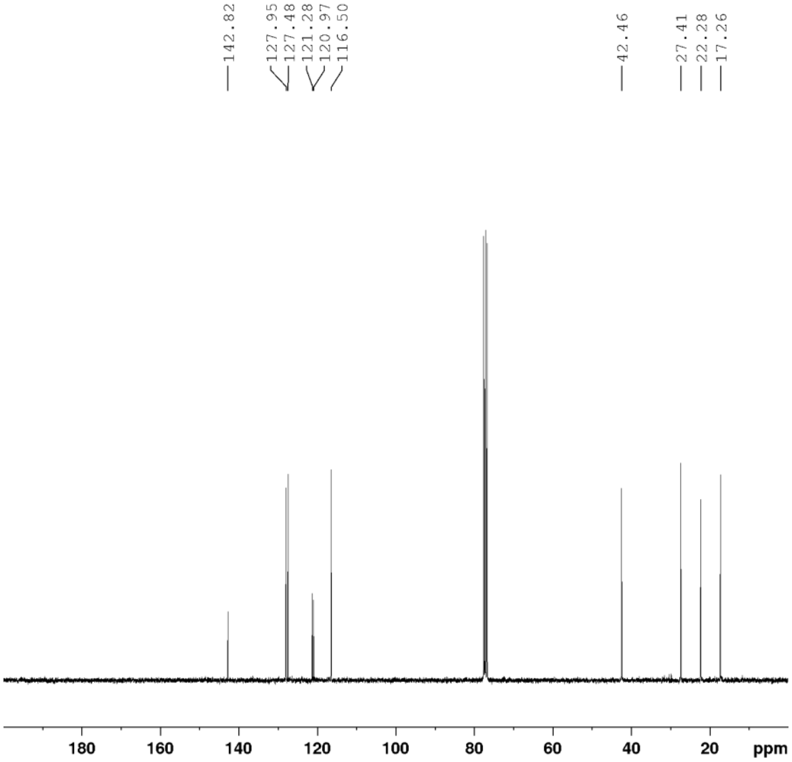

Figure S32. $^{13}C\{^{1}H\}$ NMR spectrum of **2h** in $CDCl_3$.

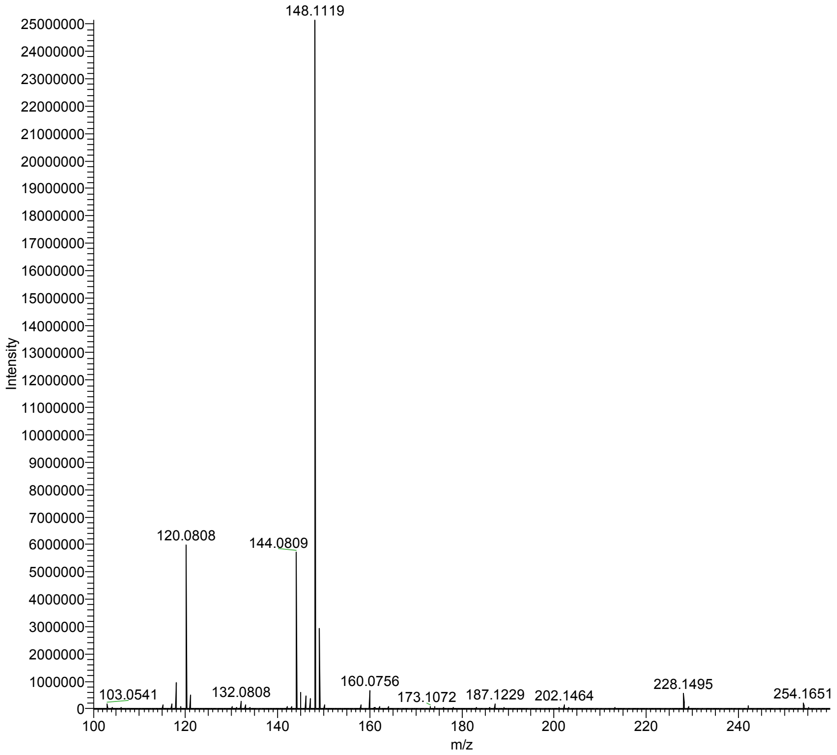

Figure S33. HRMS of **2h**.

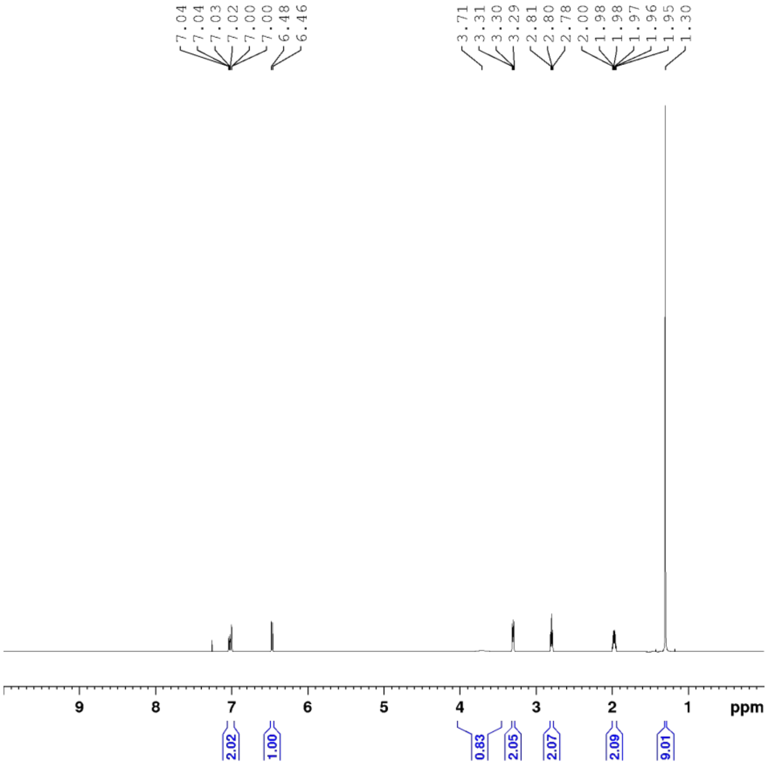

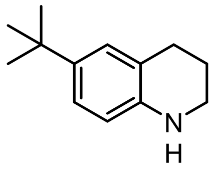

2i

Figure S34. ^{1}H NMR spectrum of **2i** in $CDCl_3$.

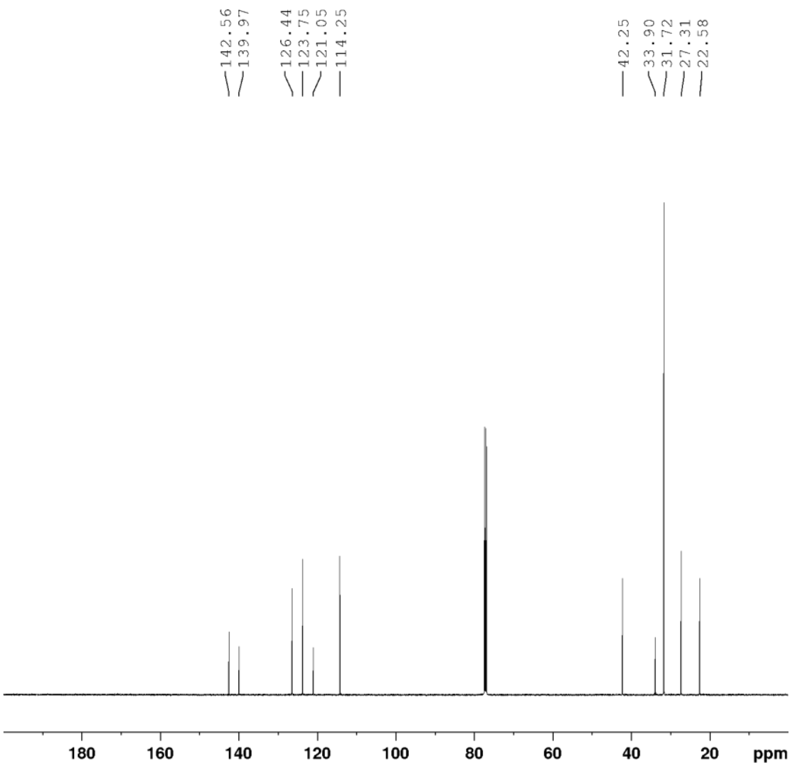

Figure S35. $^{13}C\{^{1}H\}$ NMR spectrum of **2i** in $CDCl_3$.

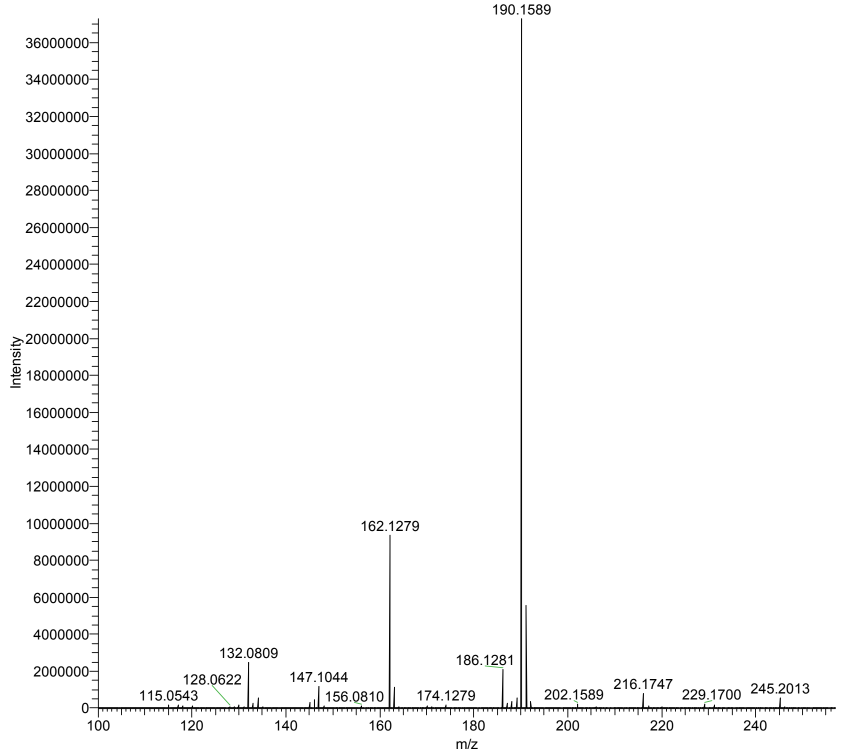

Figure S36. HRMS of **2i**.

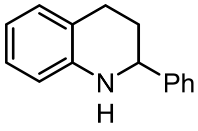

2j

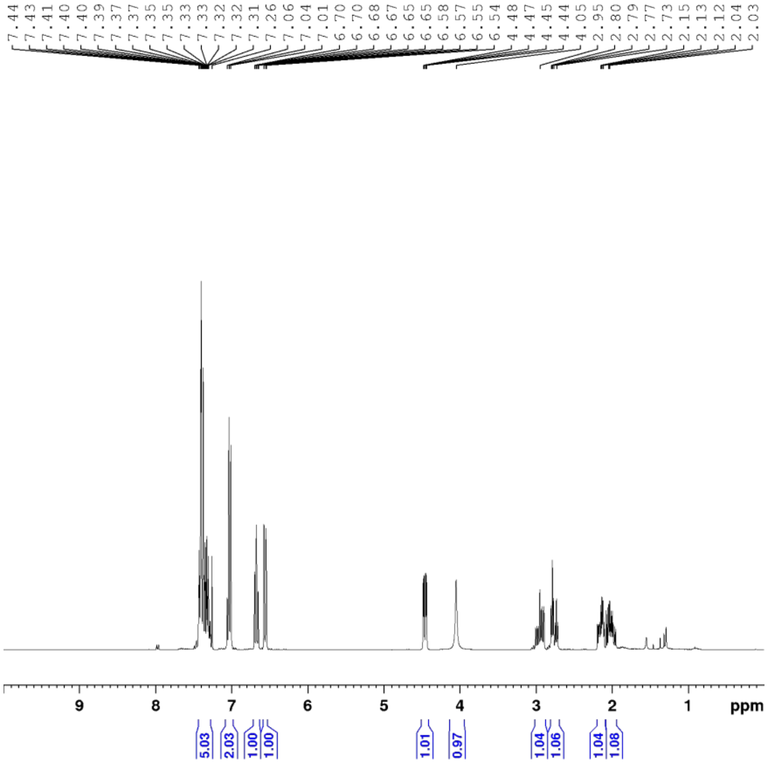

Figure S37. ^{1}H NMR spectrum of **2j** in $CDCl_3$.

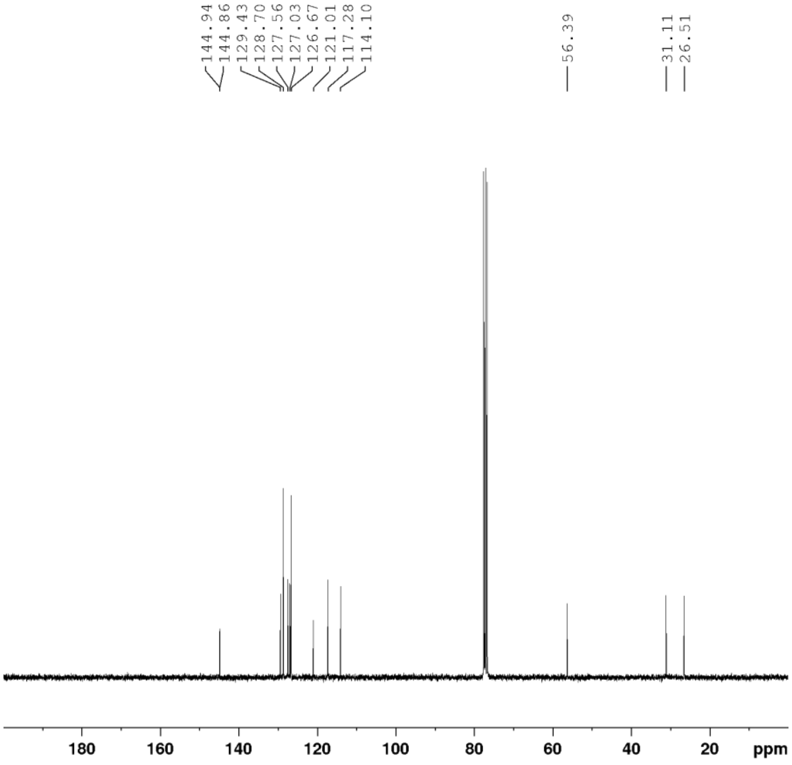

Figure S38. $^{13}C\{^{1}H\}$ NMR spectrum of **2j** in $CDCl_3$.

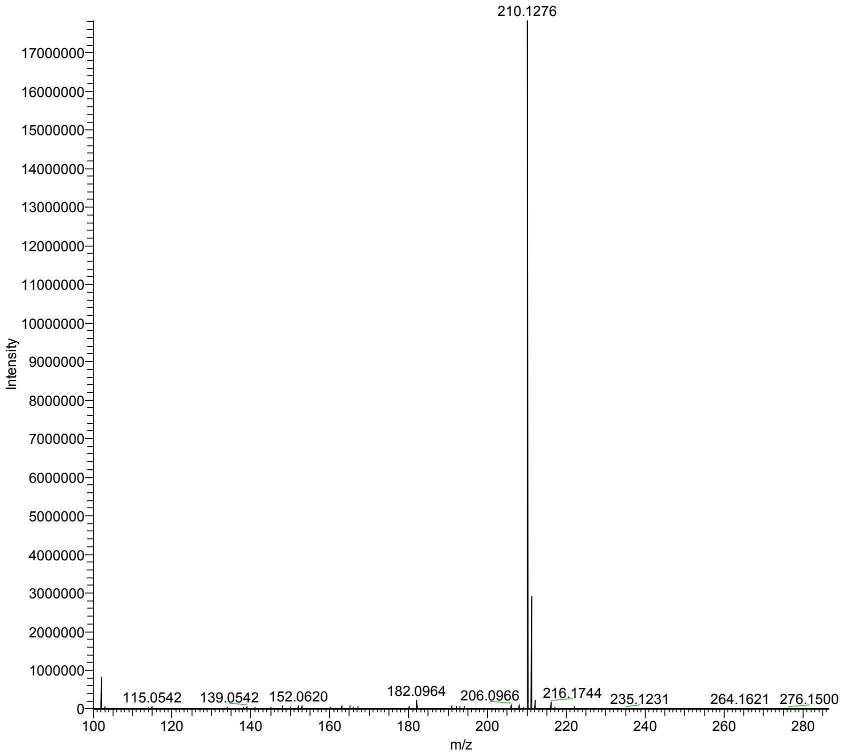

Figure S39. HRMS of **2j**.

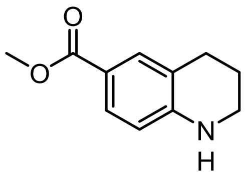

2m

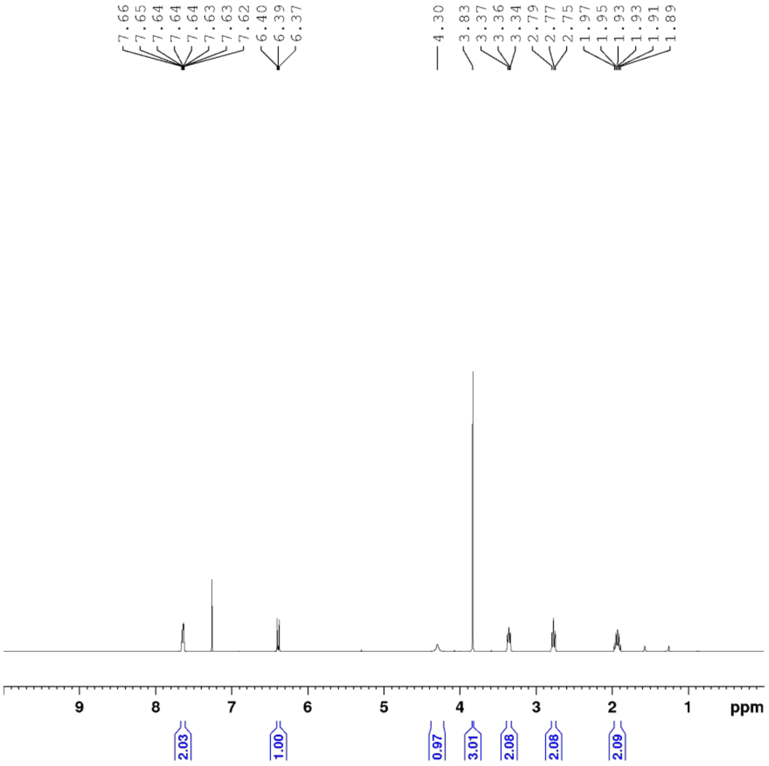

Figure S40. ^{1}H NMR spectrum of **2m** in $CDCl_3$.

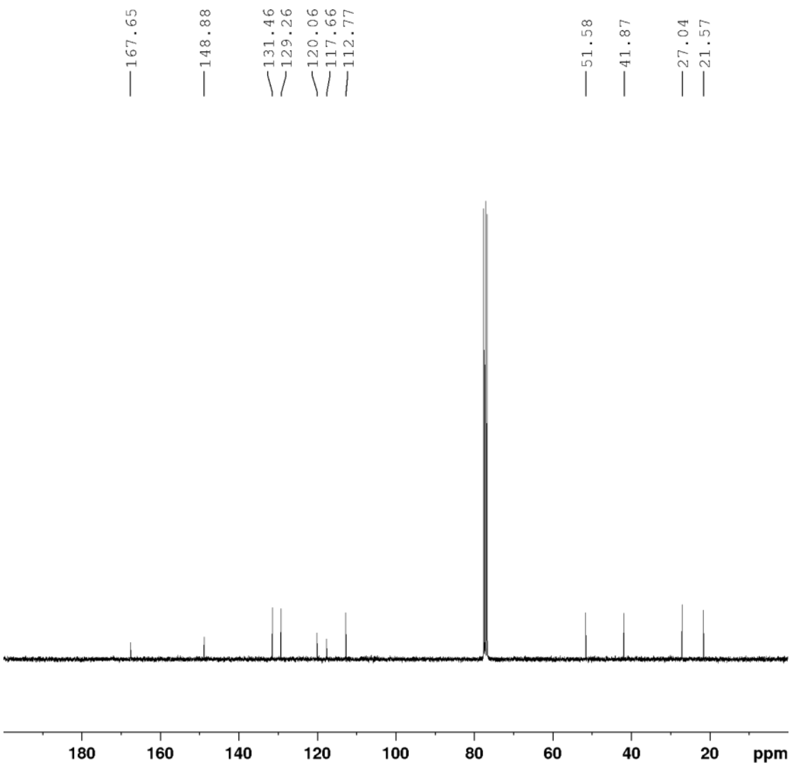

Figure S41. $^{13}C\{^{1}H\}$ NMR spectrum of **2m** in $CDCl_3$.

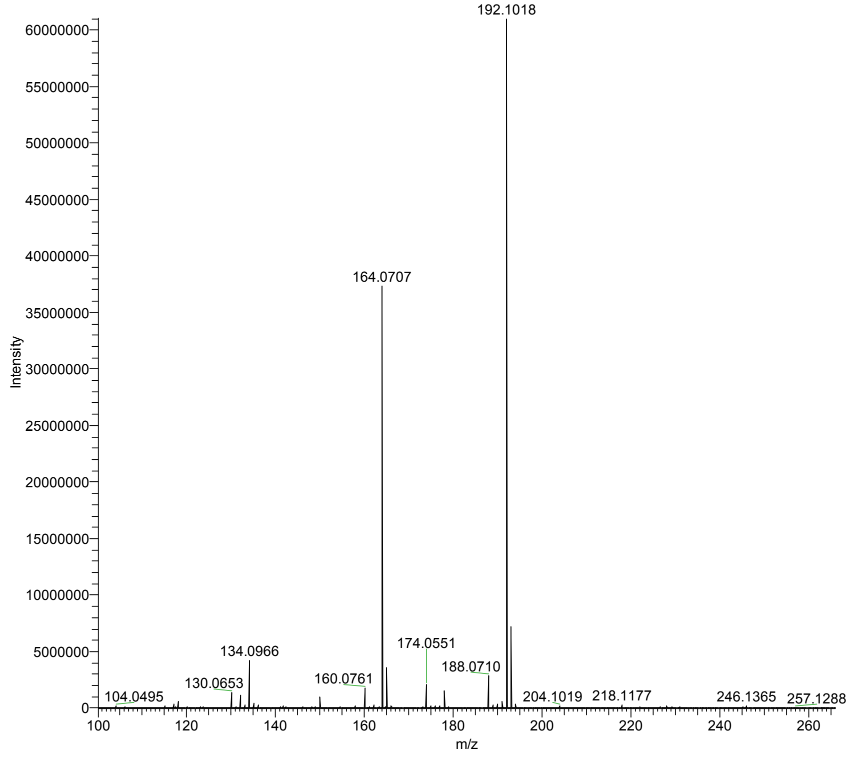

Figure S42. HRMS of **2m**.

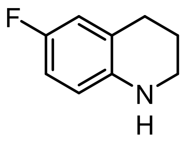

2n

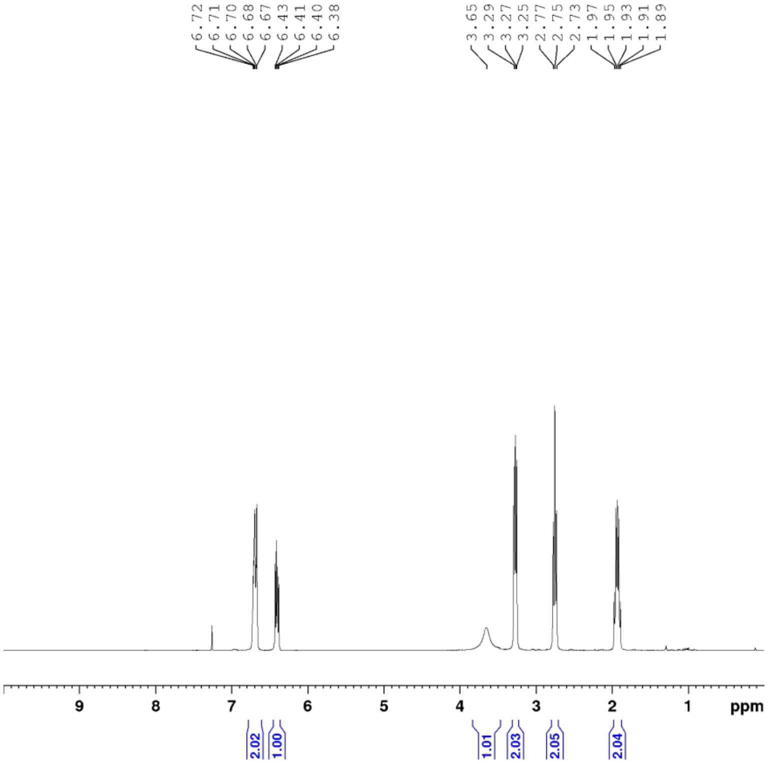

Figure S43. ^{1}H NMR spectrum of **2n** in $CDCl_3$.

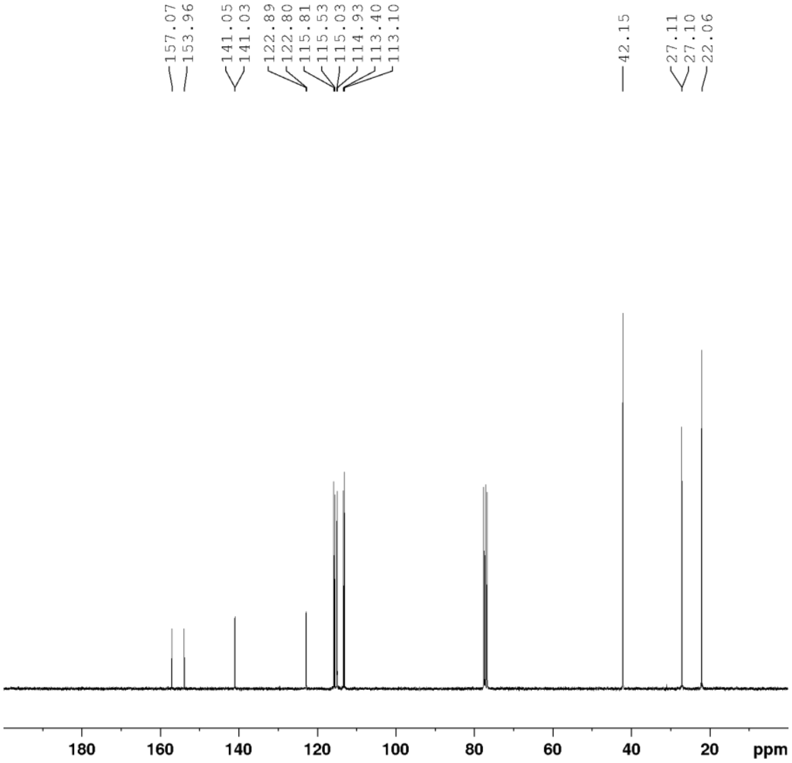

Figure S44. $^{13}C\{^{1}H\}$ NMR spectrum of **2n** in $CDCl_3$.

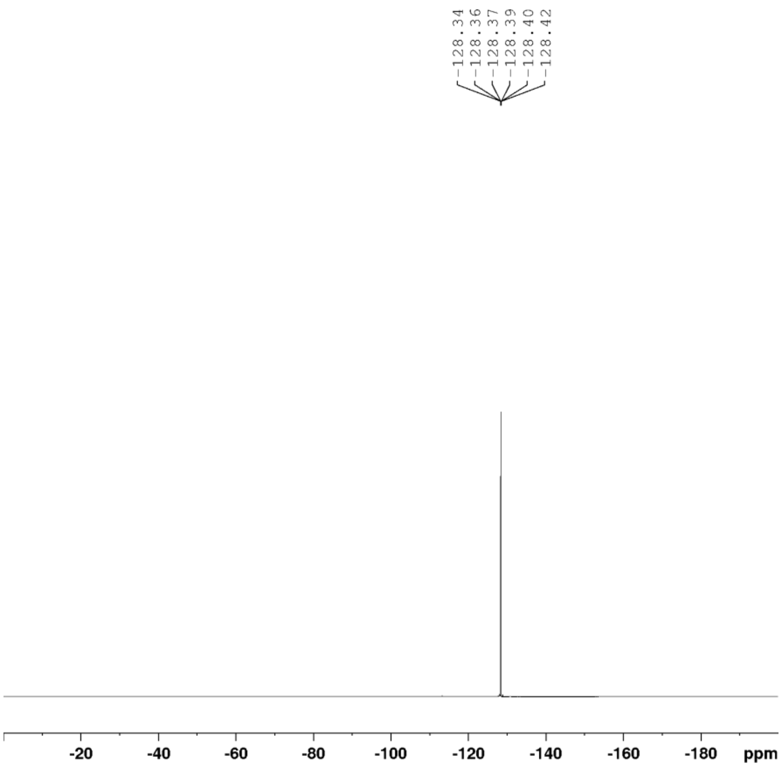

Figure S45. ^{19}F NMR spectrum of **2n** in $CDCl_3$.

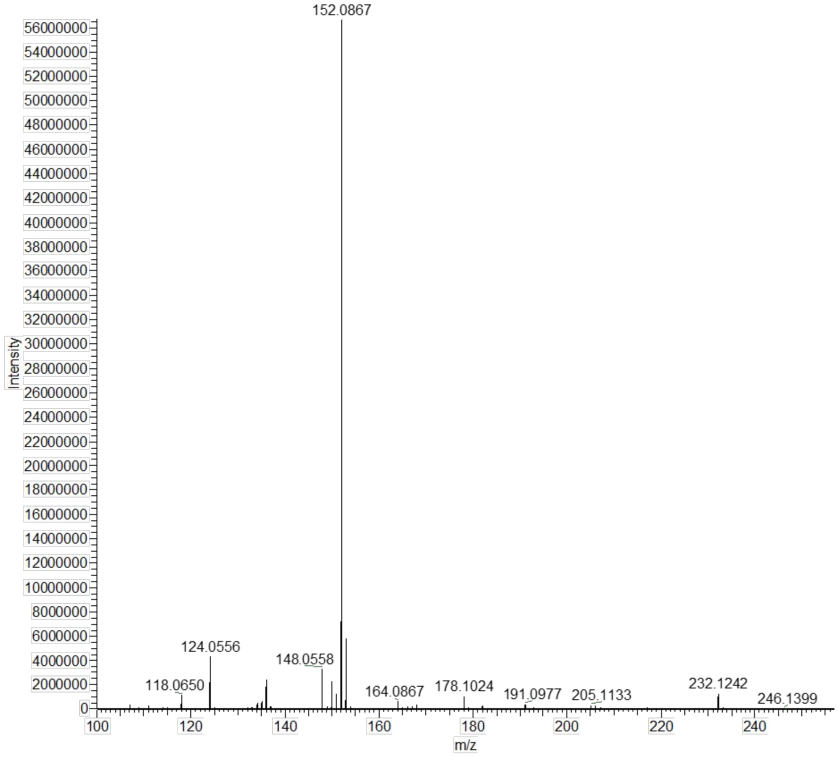

Figure S46. HRMS of **2n**.

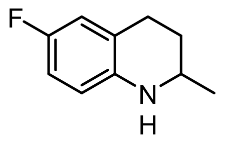

2o

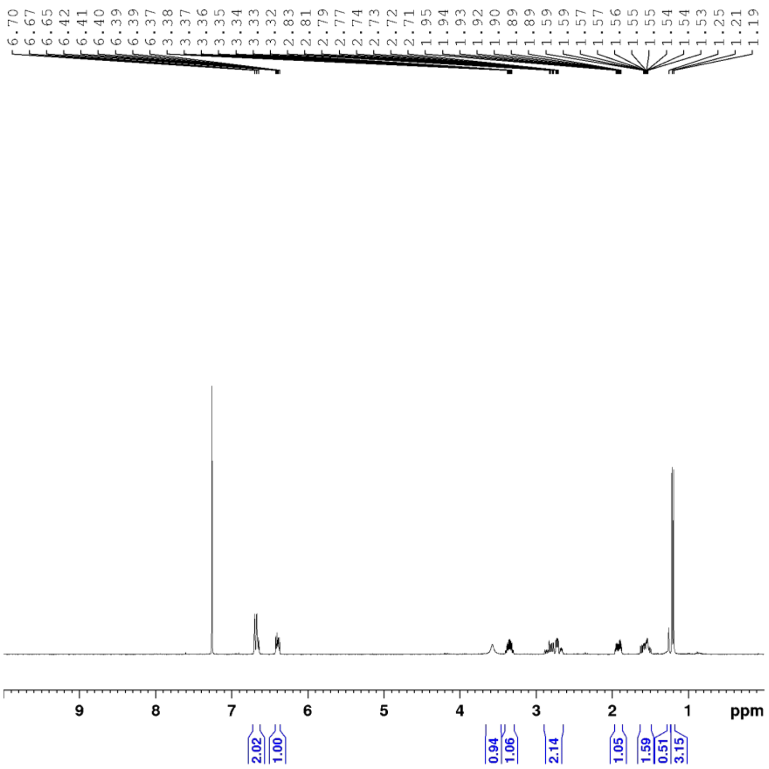

Figure S47. ^{1}H NMR spectrum of **2o** in $CDCl_3$.

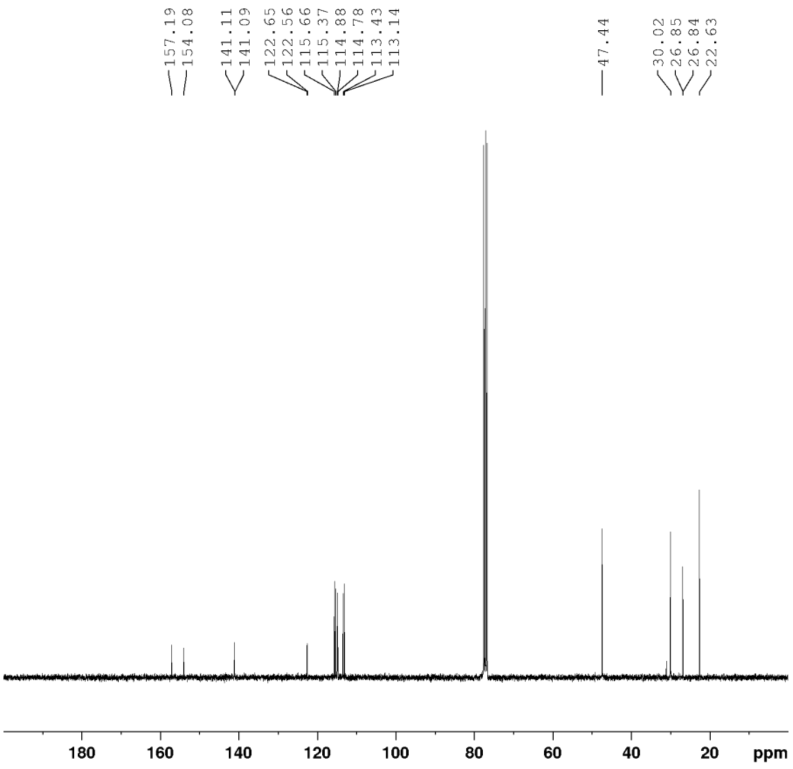

Figure S48. $^{13}C\{^{1}H\}$ NMR spectrum of **2o** in $CDCl_3$.

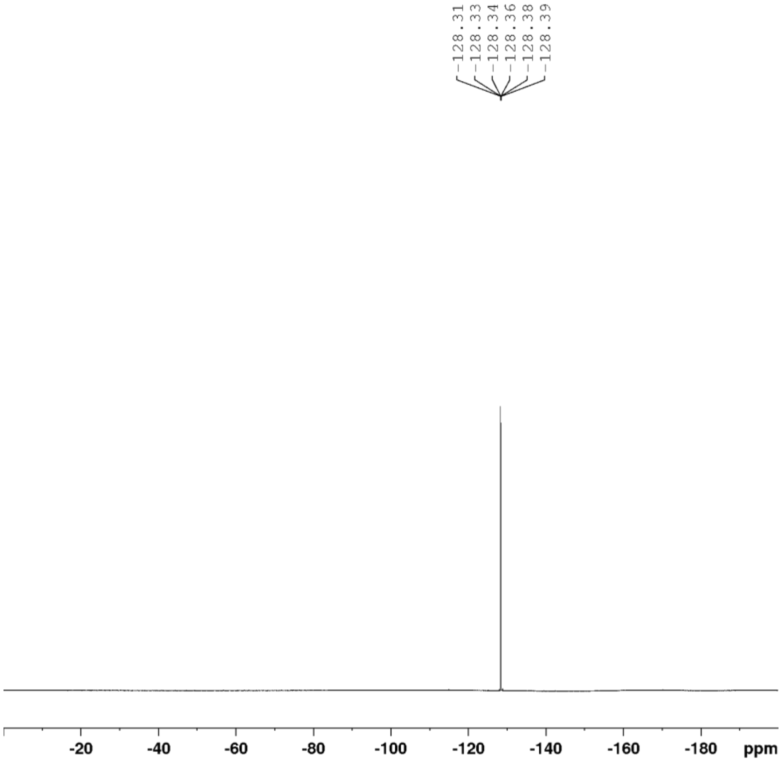

Figure S49. ^{19}F NMR spectrum of **2o** in $CDCl_3$.

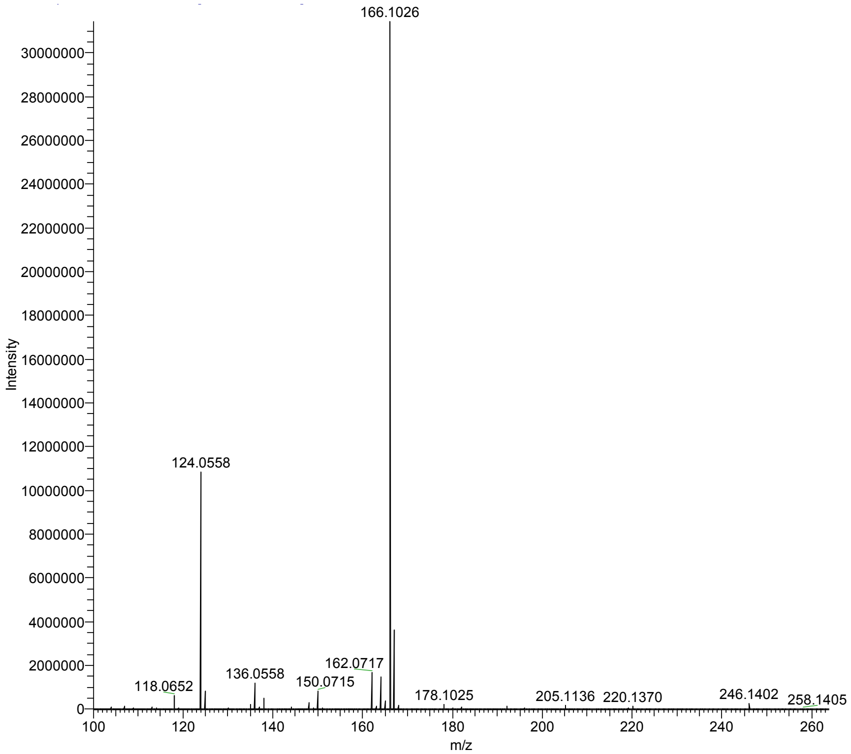

Figure S50. HRMS of **2o**.

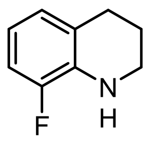

2p

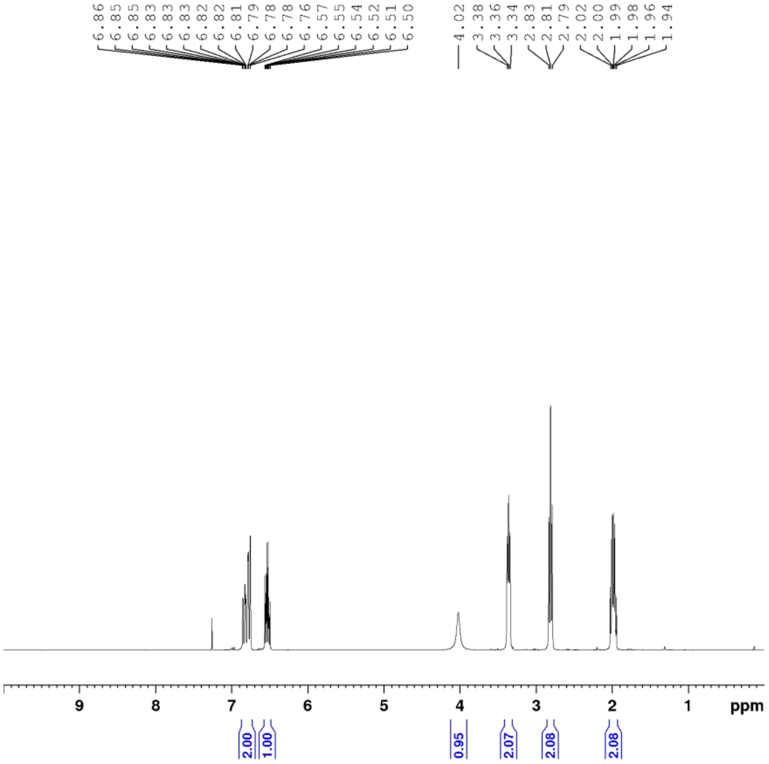

Figure S51. ^{1}H NMR spectrum of **2p** in $CDCl_3$.

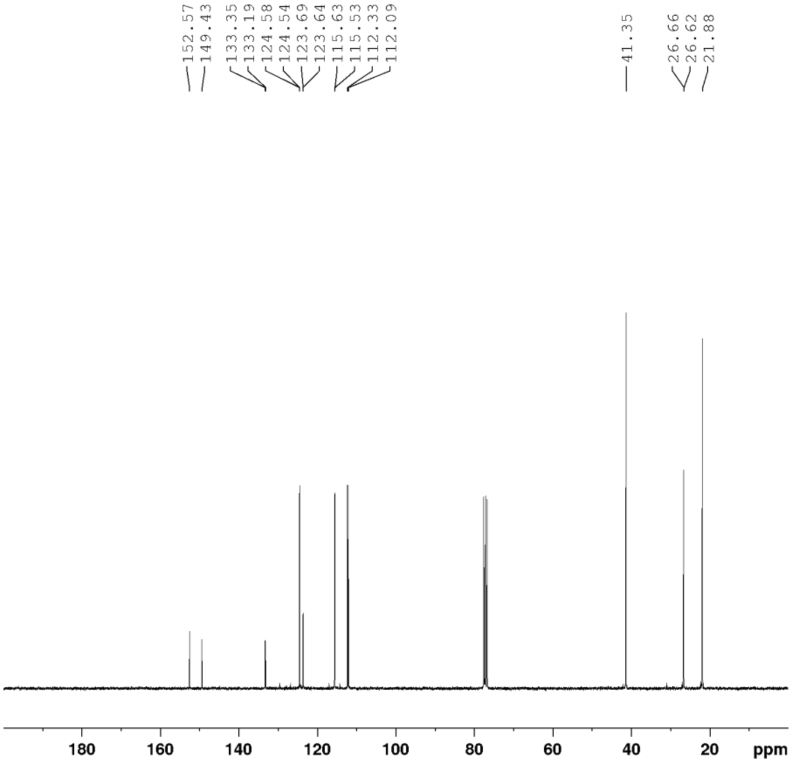

Figure S52. $^{13}C\{^{1}H\}$ NMR spectrum of **2p** in $CDCl_3$.

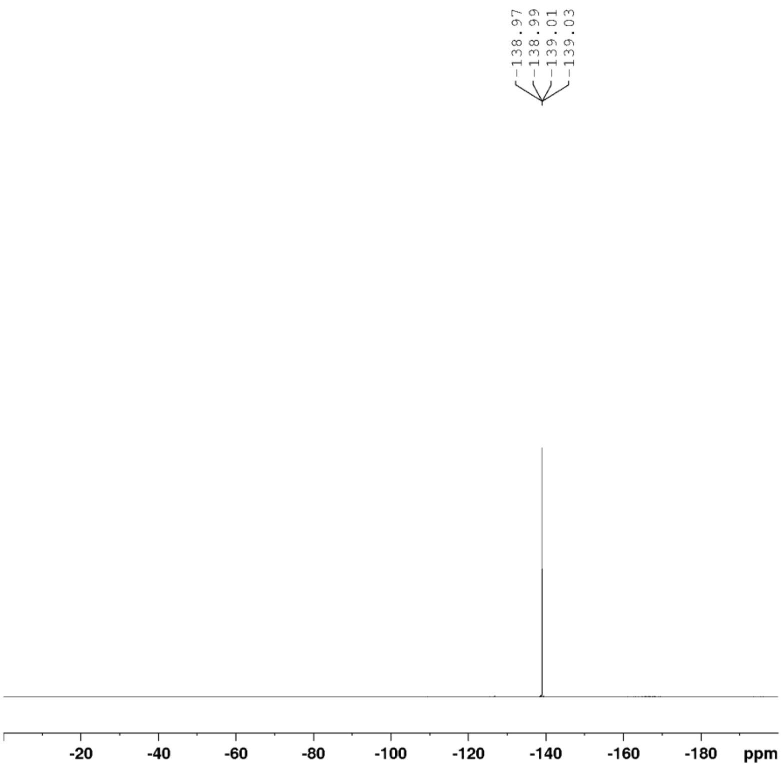

Figure S53. ^{19}F NMR spectrum of **2p** in $CDCl_3$.

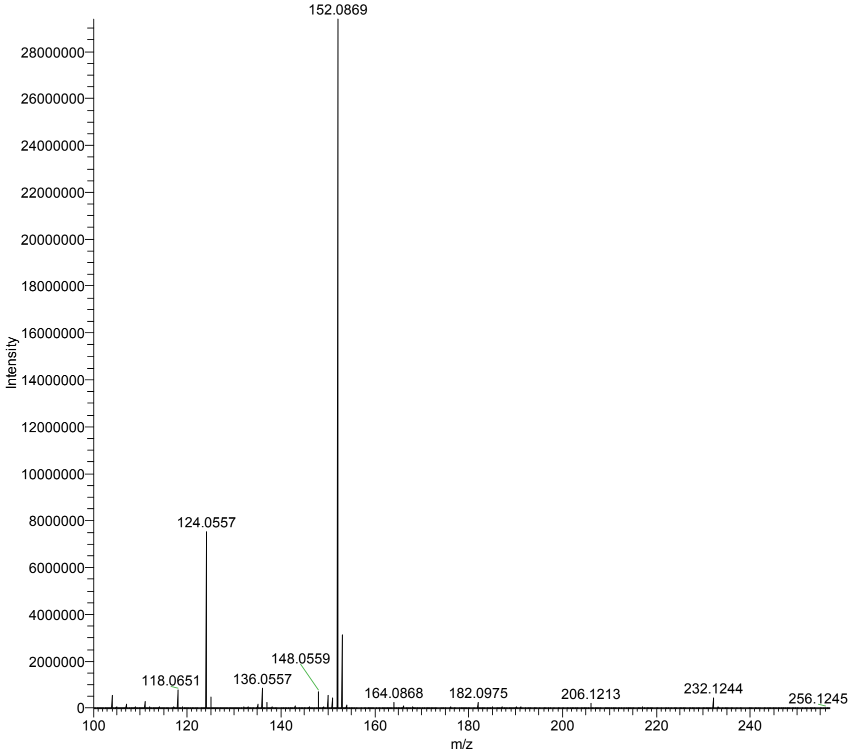

Figure S54. HRMS of **2p**.

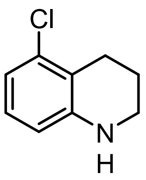

2q

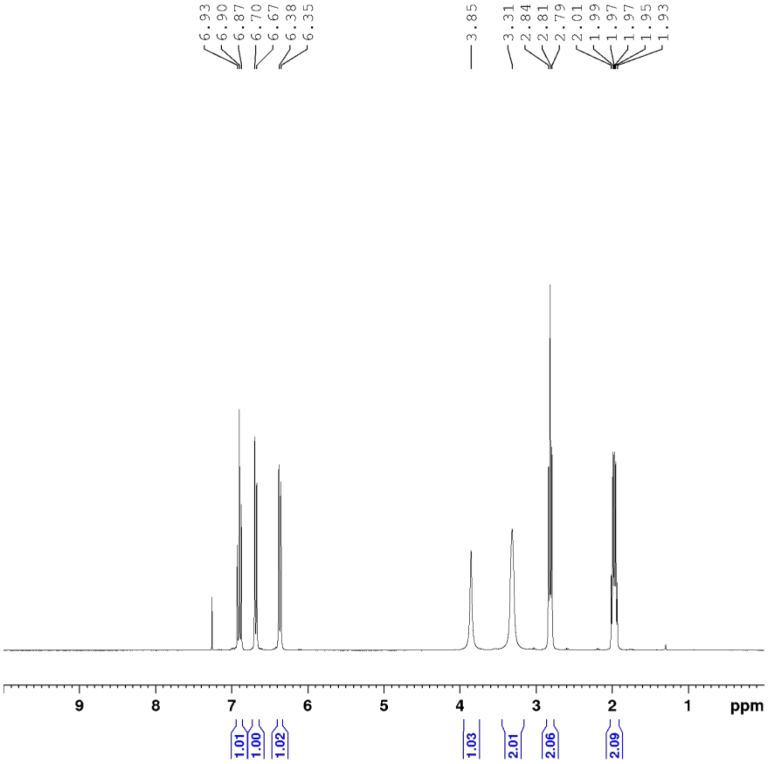

Figure S55. ^{1}H NMR spectrum of **2q** in $CDCl_3$.

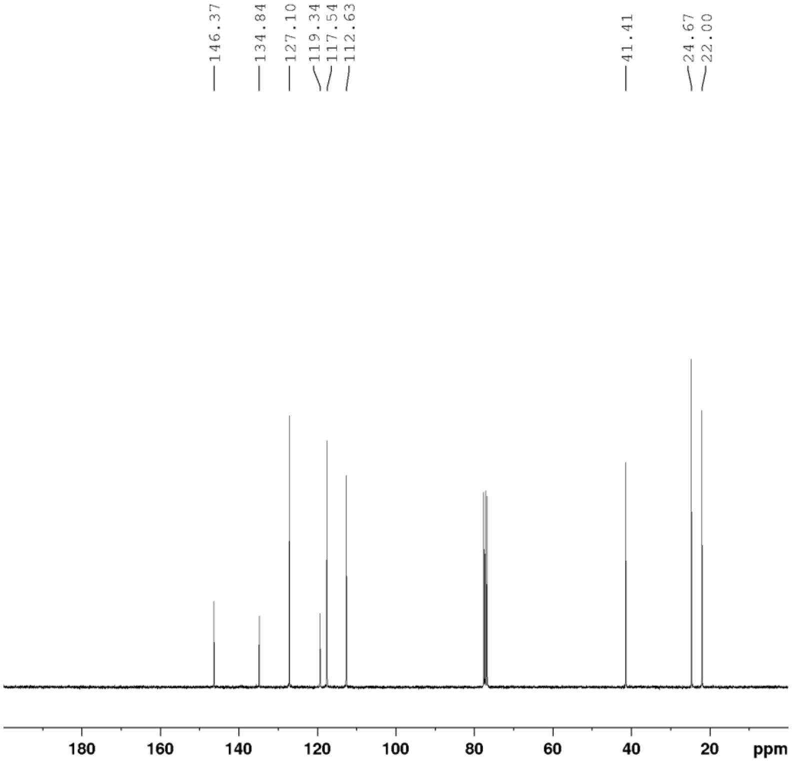

Figure S56. $^{13}C\{^{1}H\}$ NMR spectrum of **2q** in $CDCl_3$.

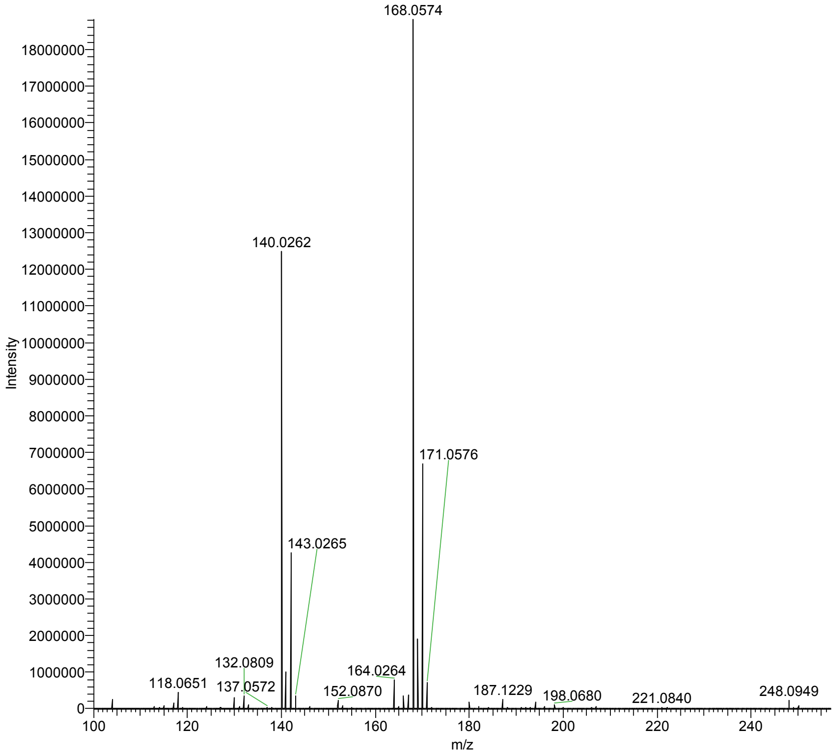

Figure S57. HRMS of **2q**.

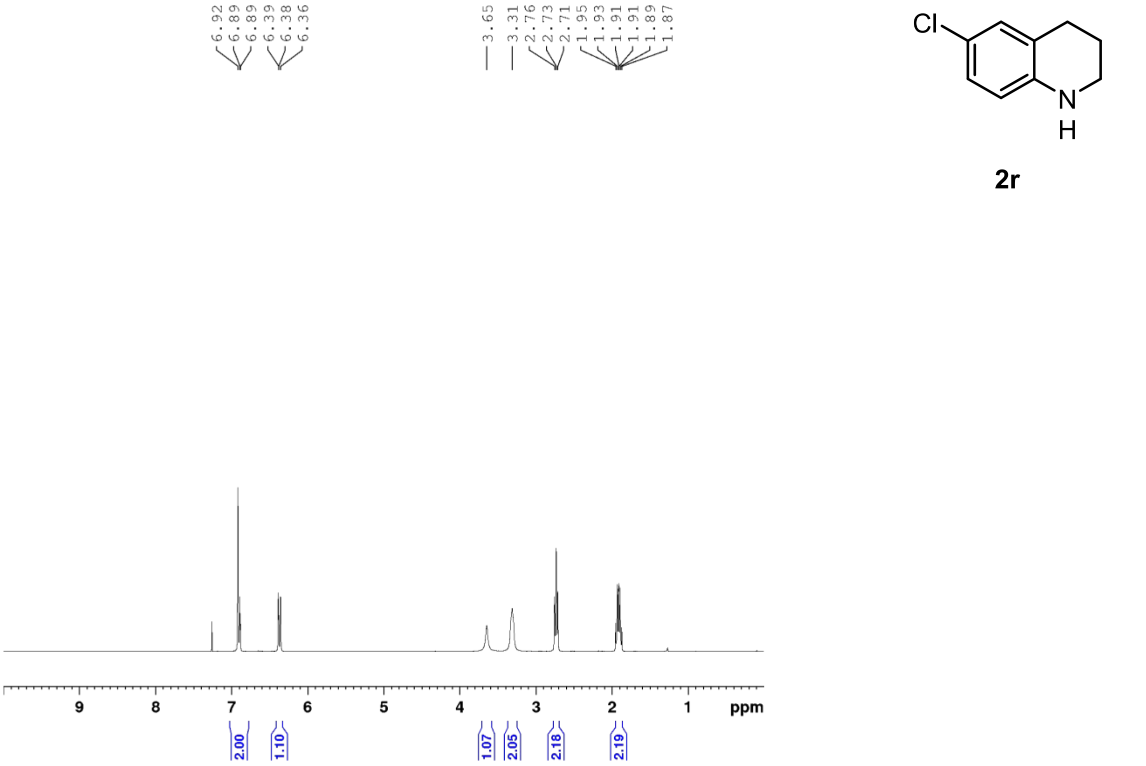

2r

Figure S58. ^{1}H NMR spectrum of **2r** in $CDCl_3$.

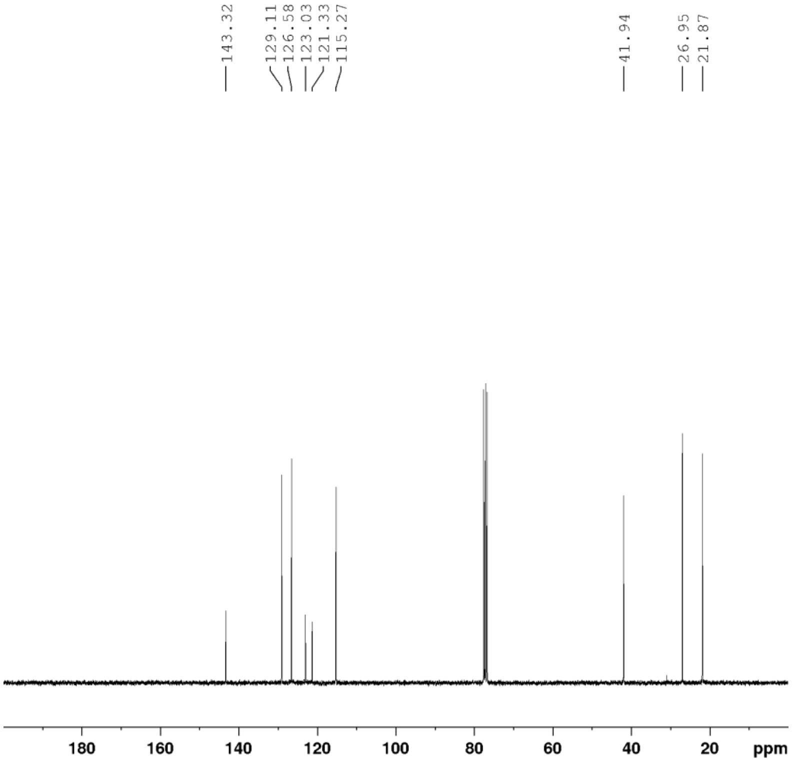

Figure S59. $^{13}C\{^1H\}$ NMR spectrum of **2r** in $CDCl_3$.

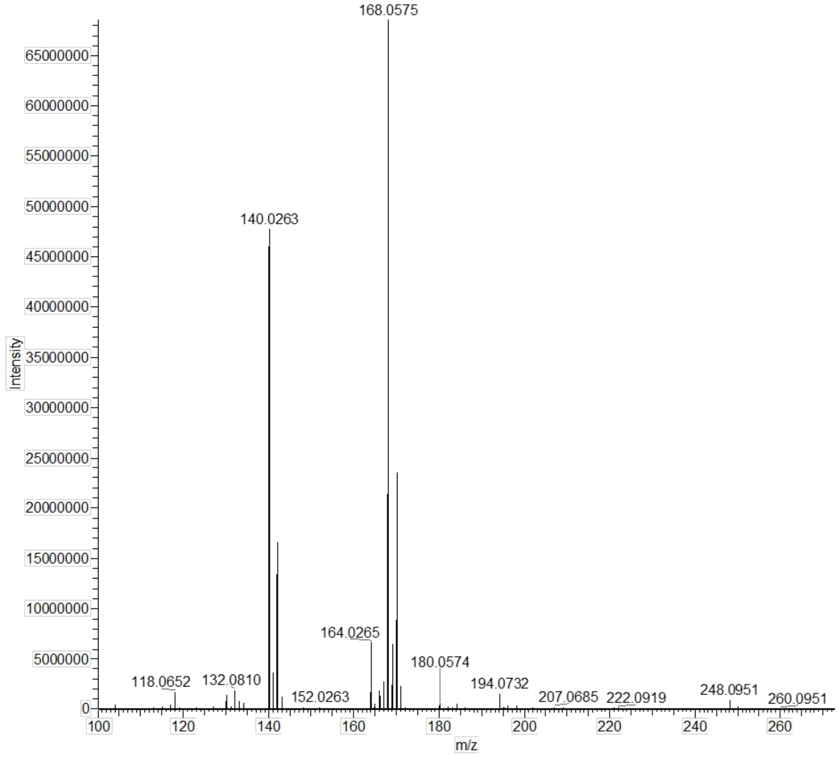

Figure S60. HRMS of **2r**.

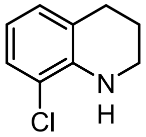

2s

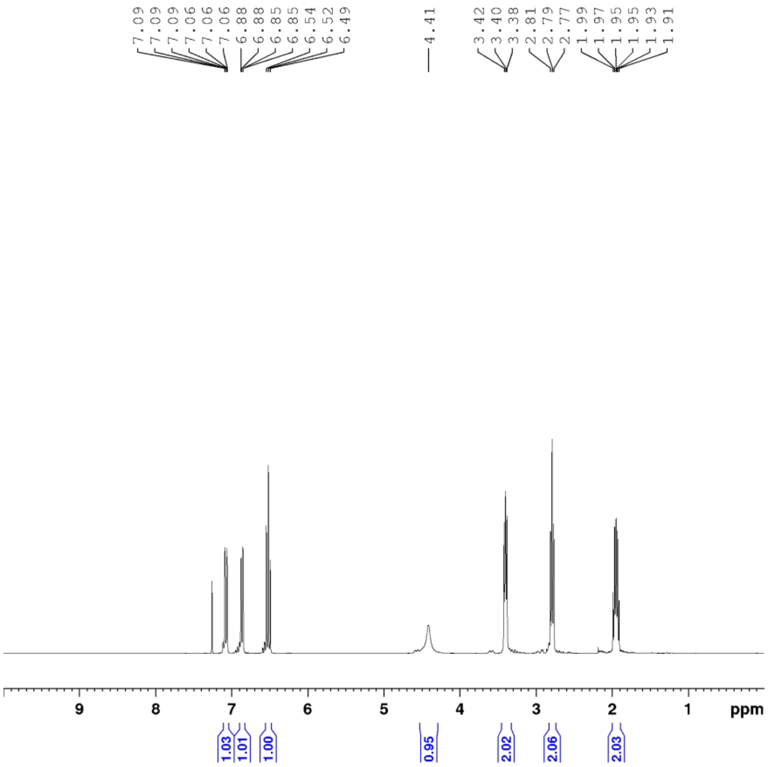

Figure S61. ^{1}H NMR spectrum of **2s** in $CDCl_3$.

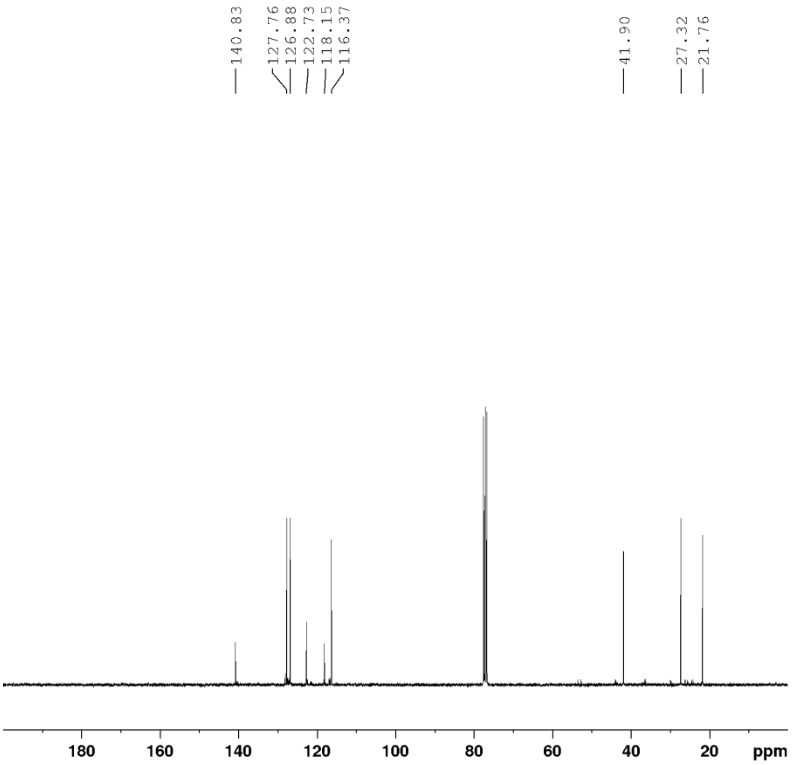

Figure S62. $^{13}C\{^{1}H\}$ NMR spectrum of **2s** in $CDCl_3$.

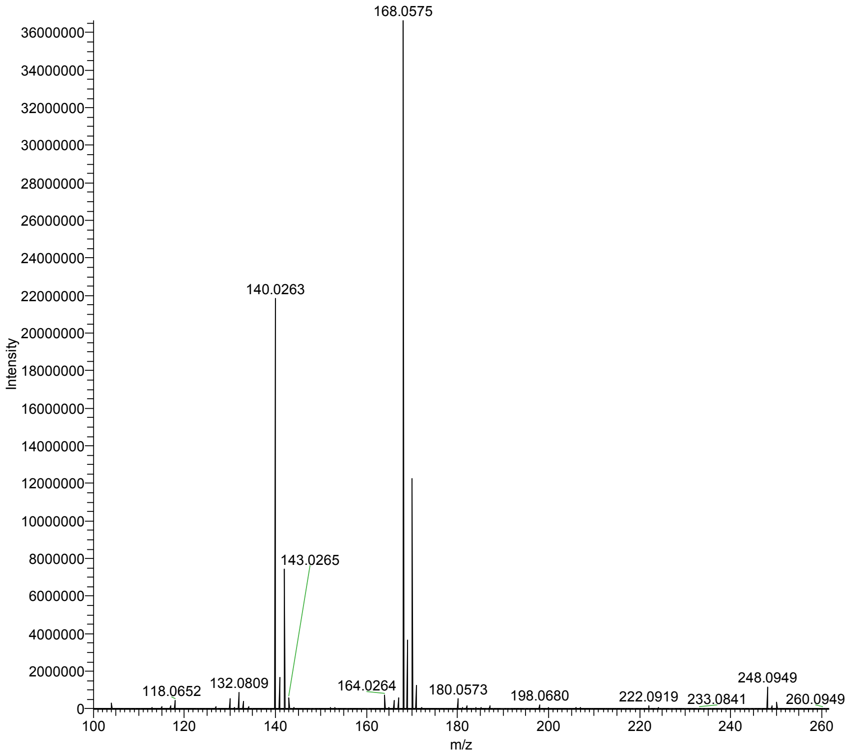

Figure S63. HRMS of **2s**.

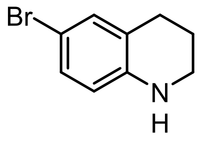

2t

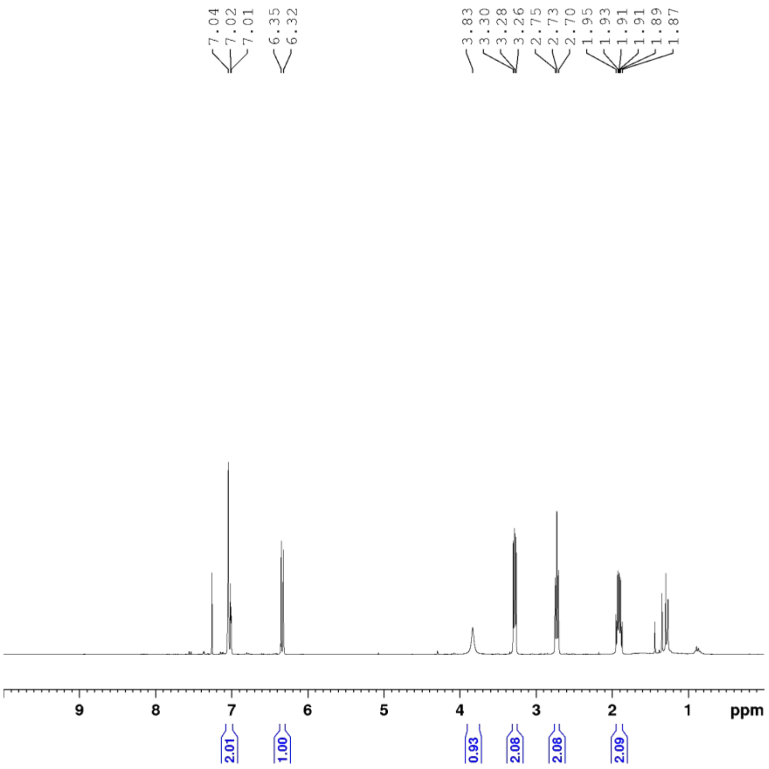

Figure S64. ^{1}H NMR spectrum of **2t** in $CDCl_3$.

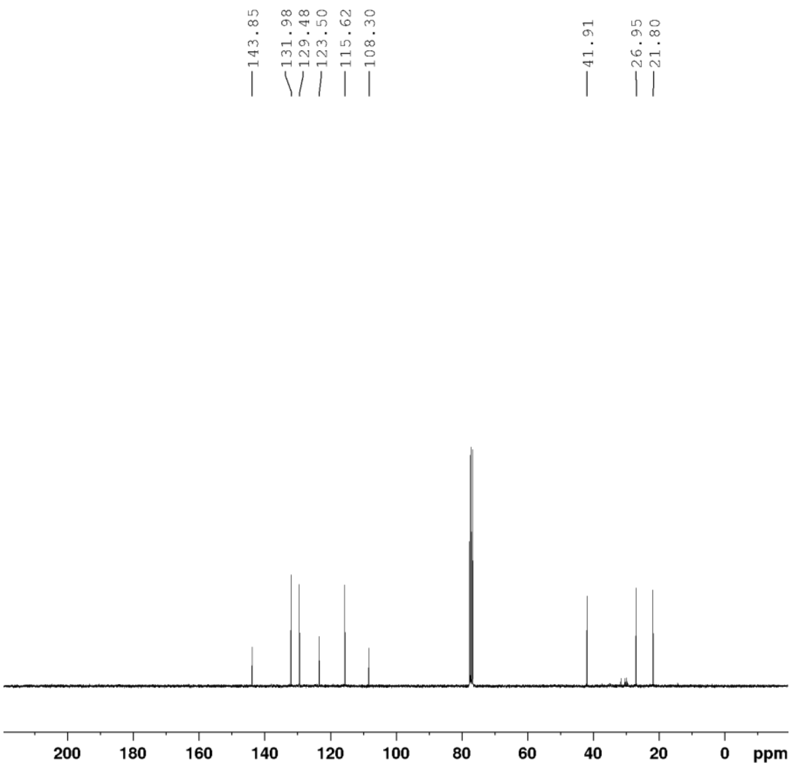

Figure S65. $^{13}C\{^{1}H\}$ NMR spectrum of **2t** in $CDCl_3$.

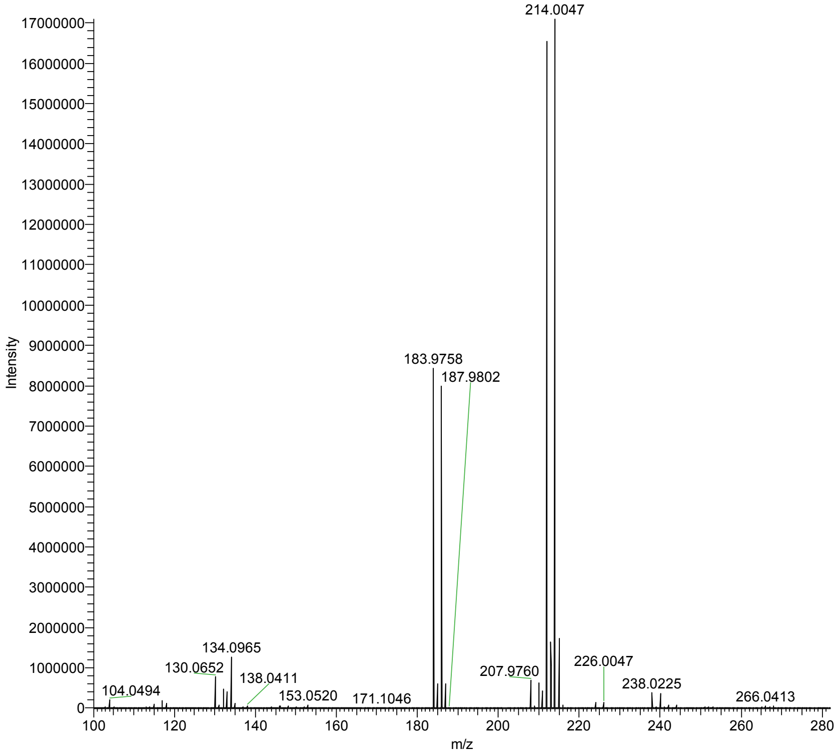

Figure S66. HRMS of **2t** (dominant peak corresponds to ^{81}Br).

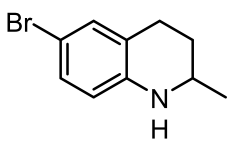

2u

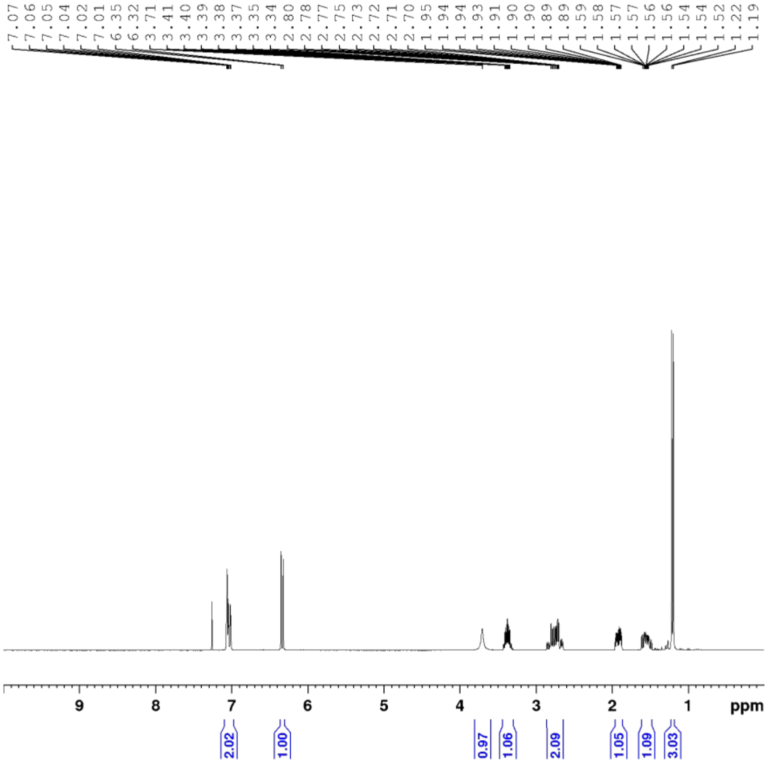

Figure S67. ^{1}H NMR spectrum of **2u** in $CDCl_3$.

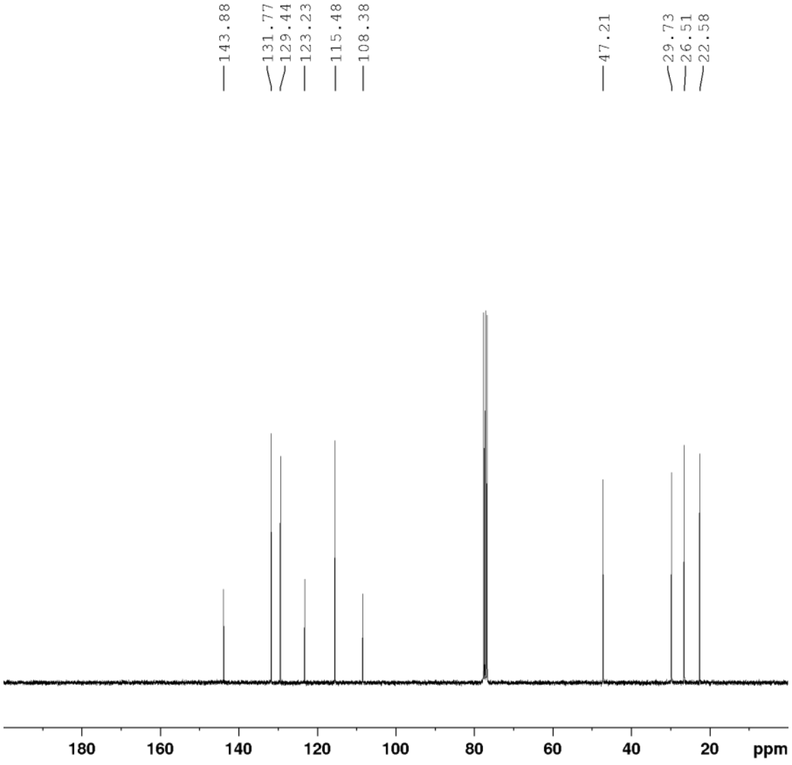

Figure S68. $^{13}C\{^{1}H\}$ NMR spectrum of **2u** in $CDCl_3$.

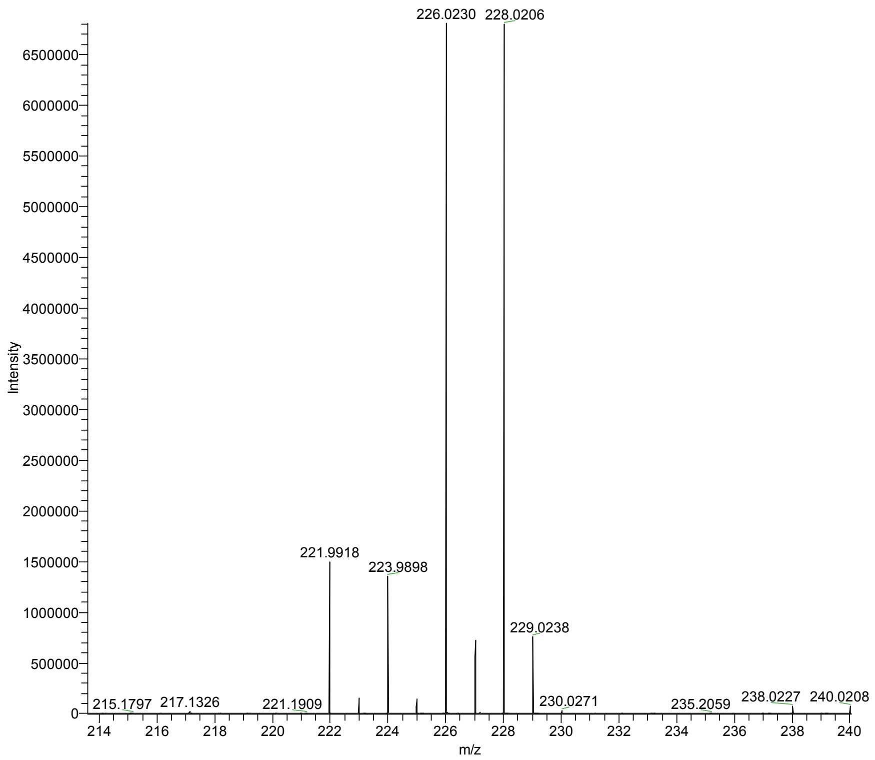

Figure S69. HRMS of **2u**.

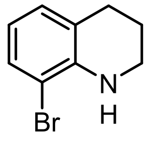

2v

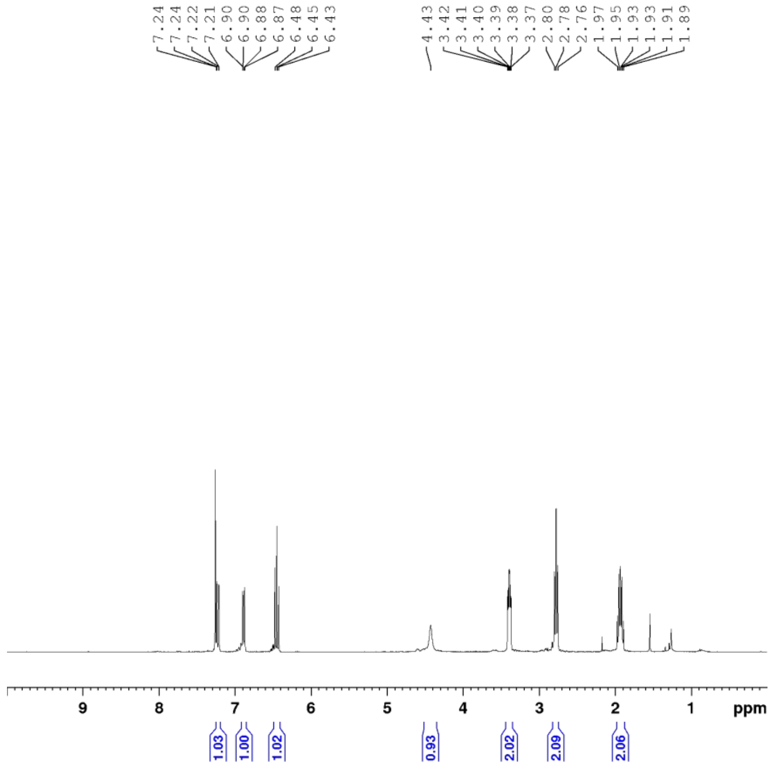

Figure S70. ^{1}H NMR spectrum of **2v** in $CDCl_3$.

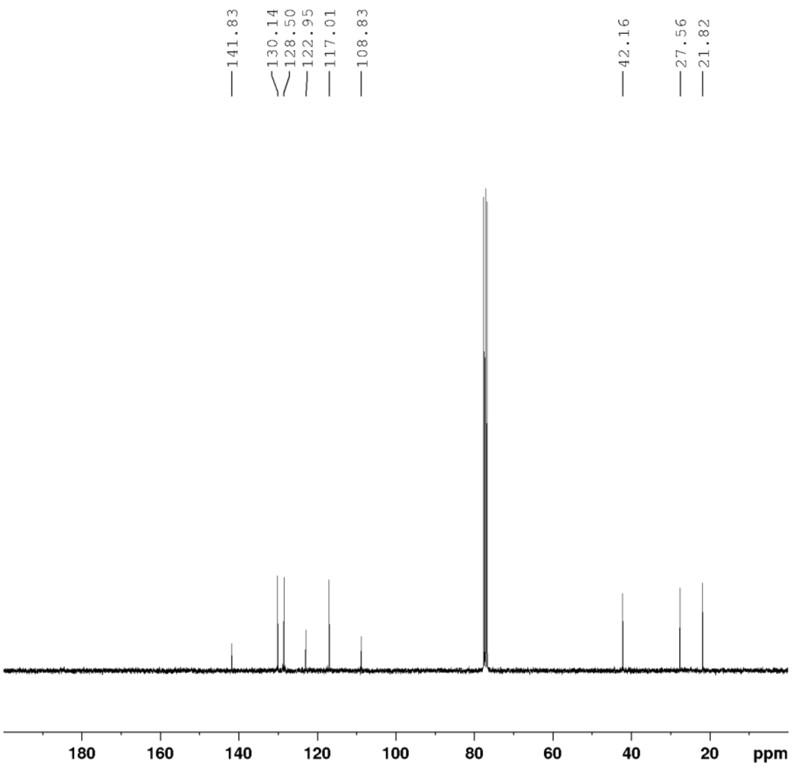

Figure S71. $^{13}C\{^1H\}$ NMR spectrum of **2v** in $CDCl_3$.

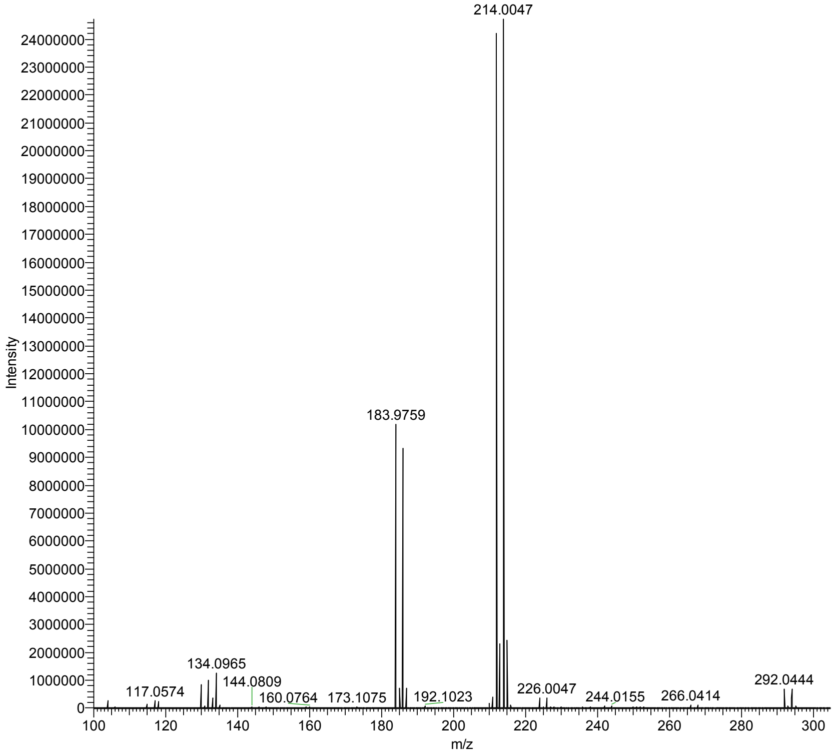

Figure S72. HRMS of **2v** (dominant peak corresponds to ^{81}Br).

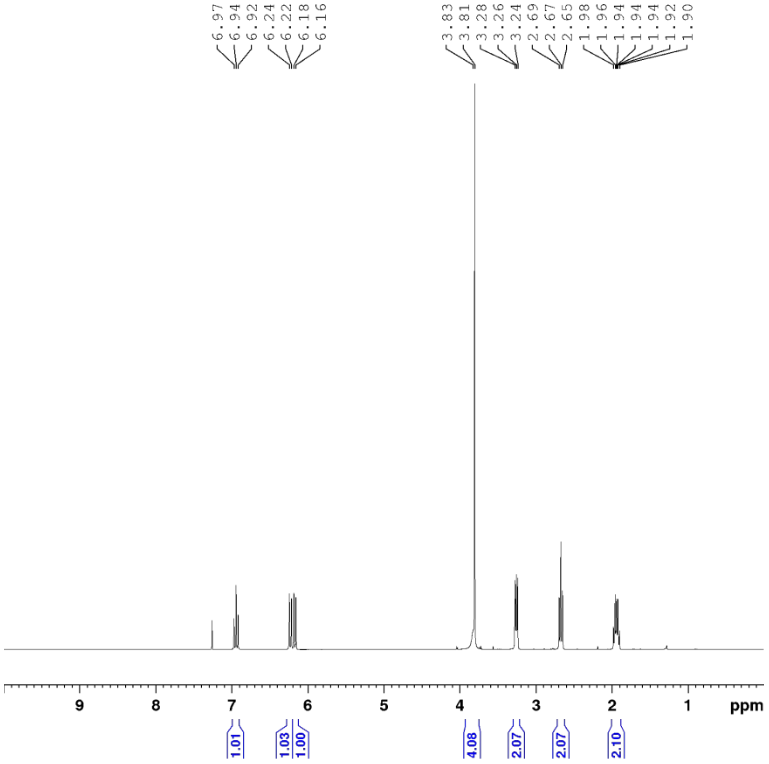

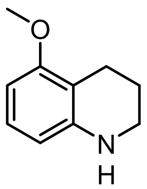

2w

Figure S73. ^{1}H NMR spectrum of **2w** in $CDCl_3$.

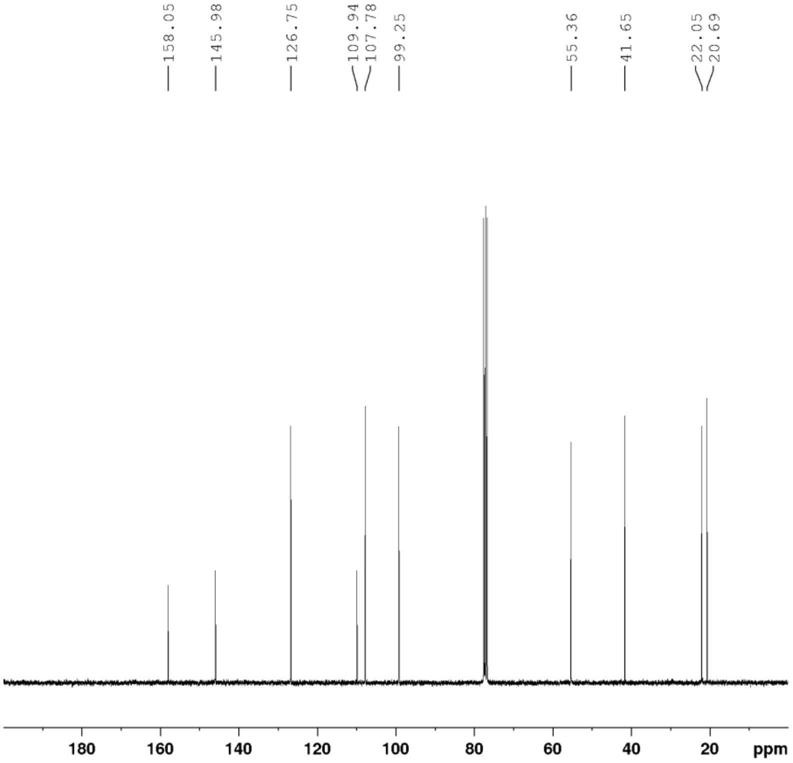

Figure S74. $^{13}C\{^{1}H\}$ NMR spectrum of **2w** in $CDCl_3$.

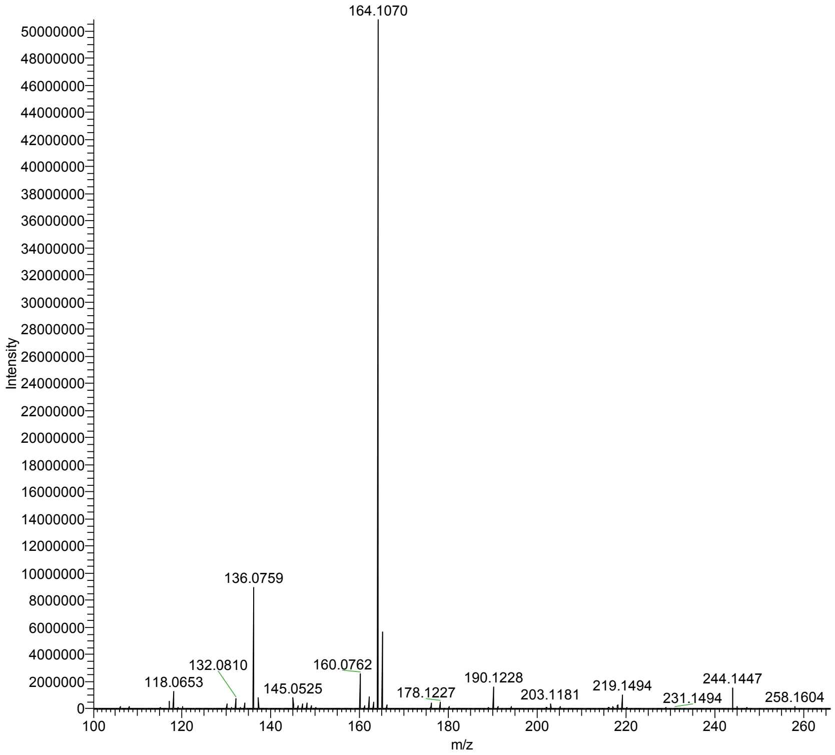

Figure S75. HRMS of **2w**.

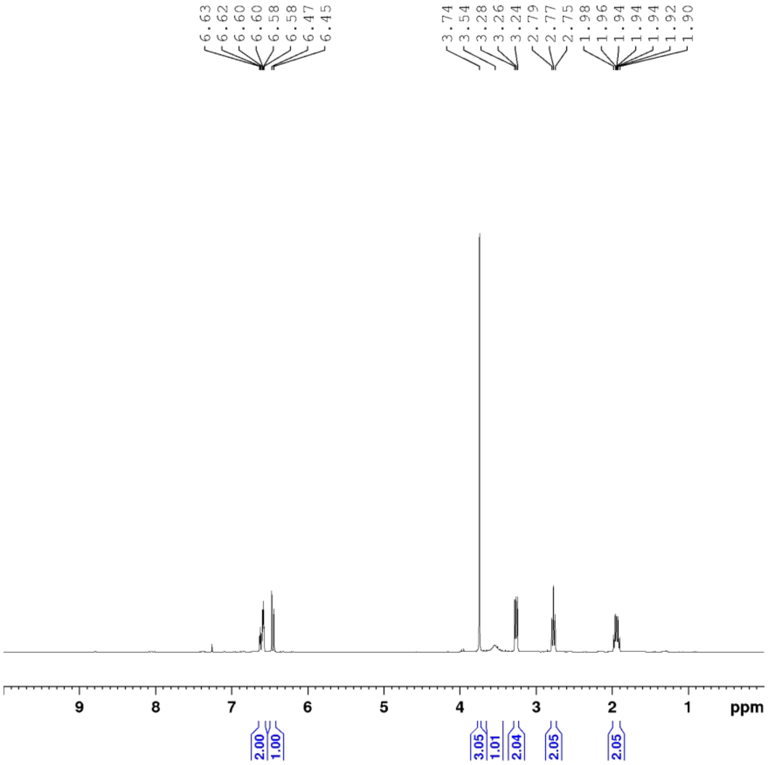

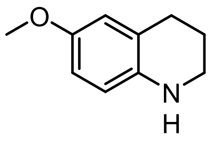

2x

Figure S76. ^{1}H NMR spectrum of **2x** in $CDCl_3$.

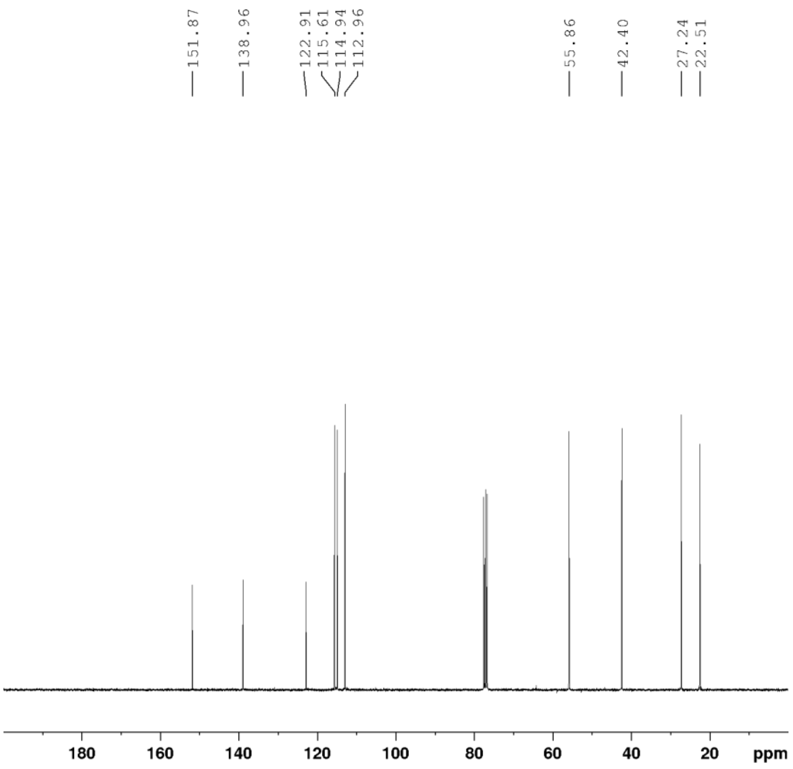

Figure S77. $^{13}C\{^{1}H\}$ NMR spectrum of **2x** in $CDCl_3$.

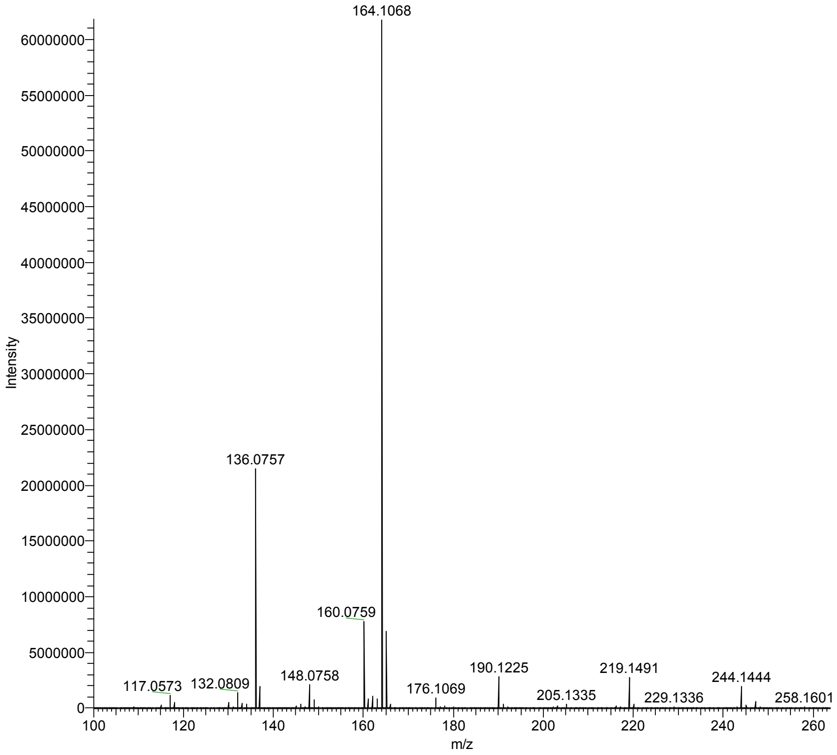

Figure S78. HRMS of **2x**.

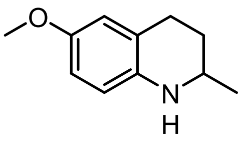

2y

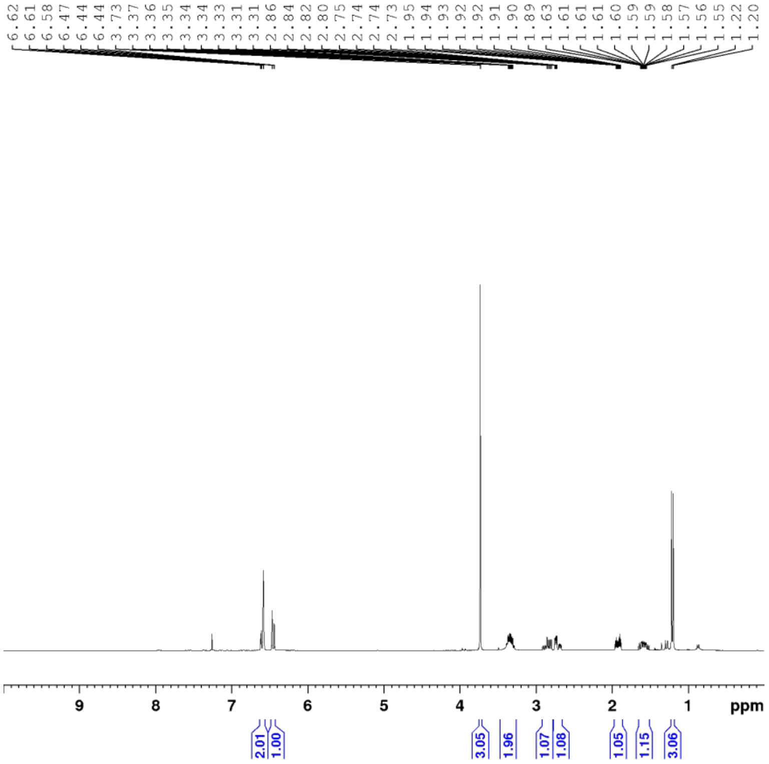

Figure S79. ^{1}H NMR spectrum of **2y** in $CDCl_3$.

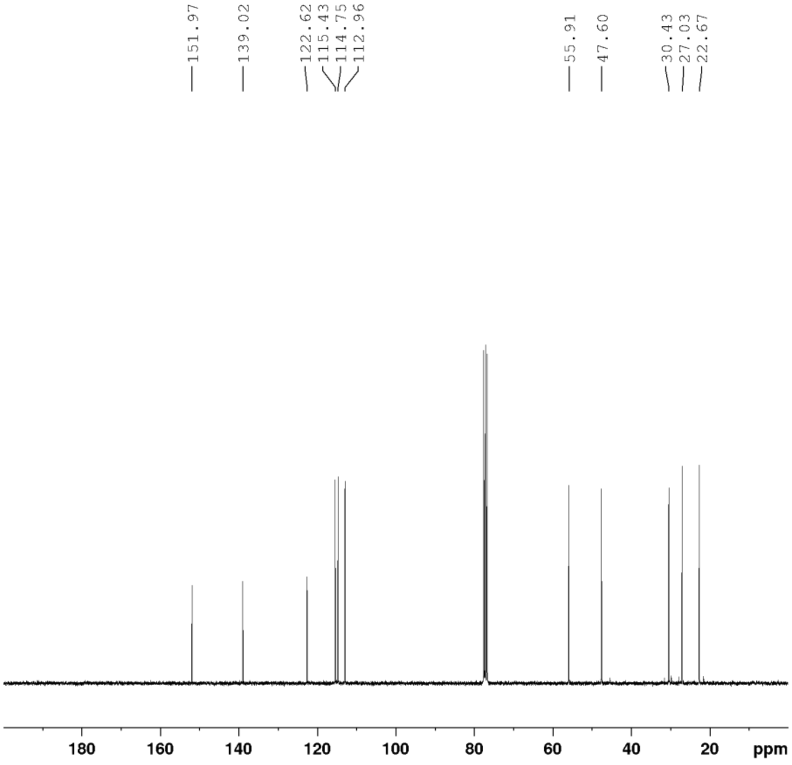

Figure S80. $^{13}C\{^{1}H\}$ NMR spectrum of **2y** in $CDCl_3$.

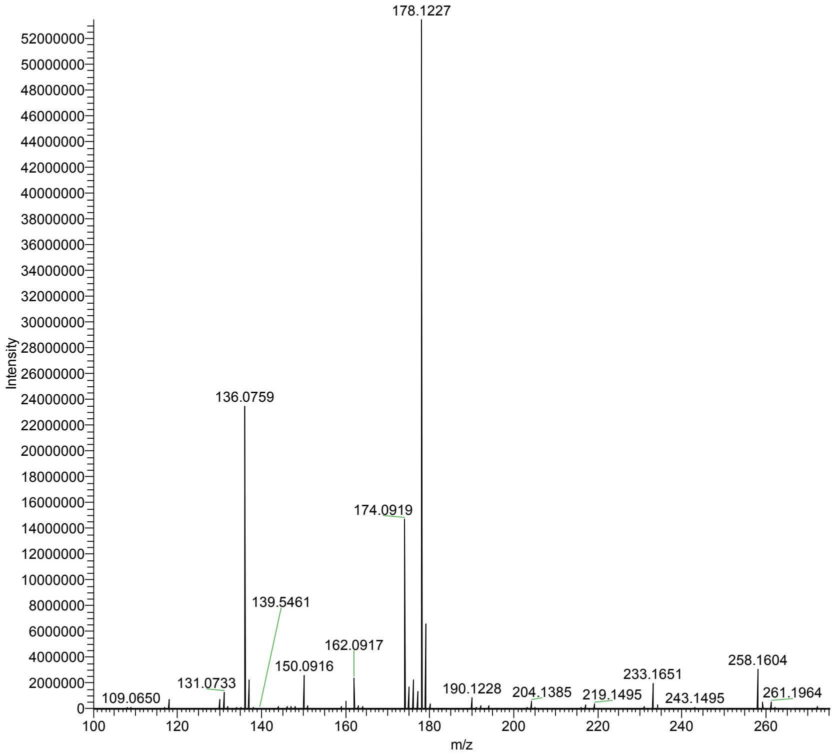

Figure S81. HRMS of **2y**.

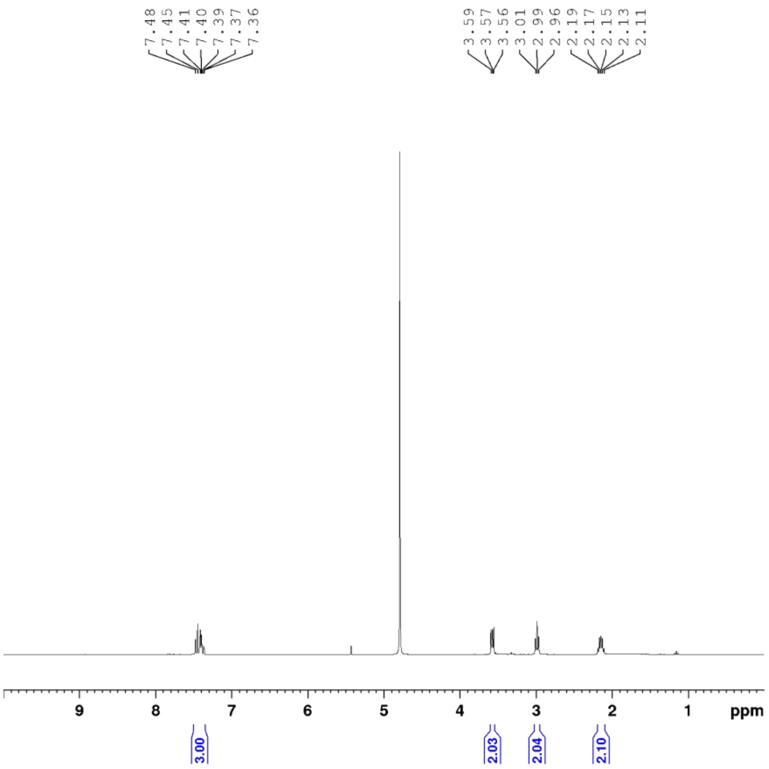

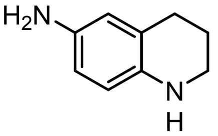

2z

Figure S82. ^{1}H NMR spectrum of the hydrochloride salt of **2z** in D_2O.

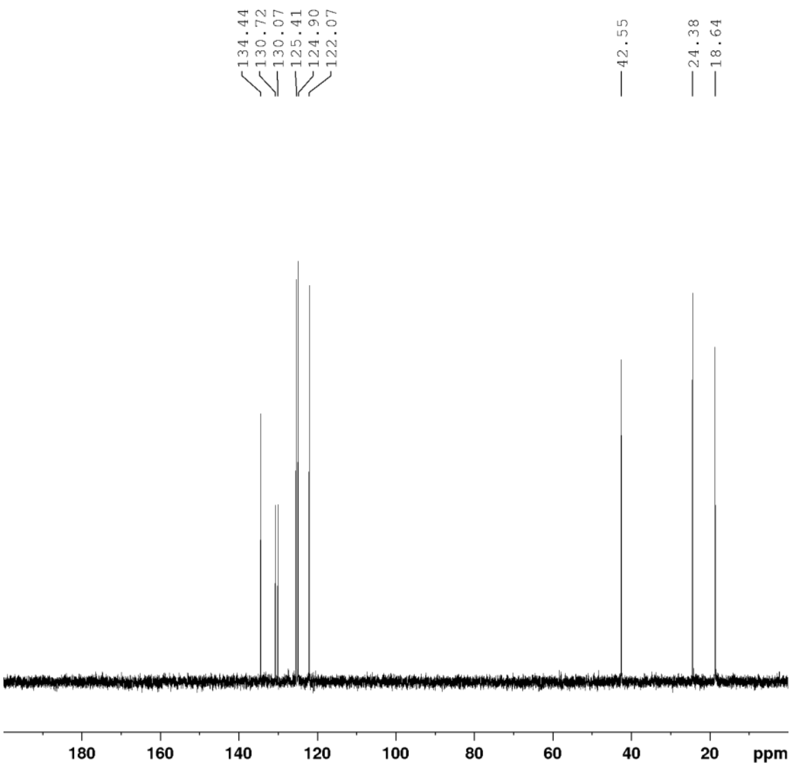

Figure S83. $^{13}C\{^1H\}$ NMR spectrum of the hydrochloride salt of **2z** in D_2O.

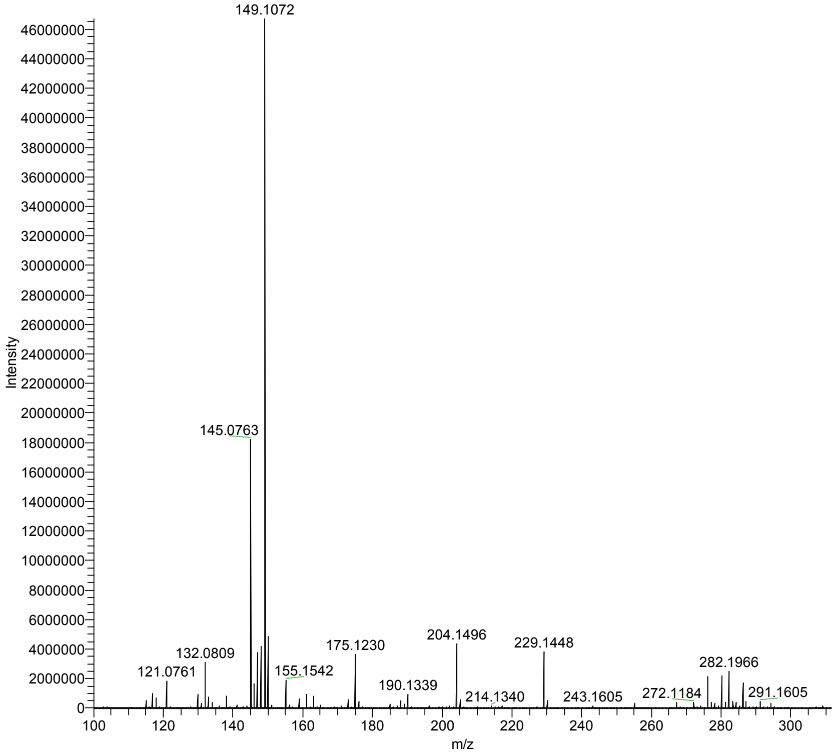

Figure S84. HRMS of **2z**.

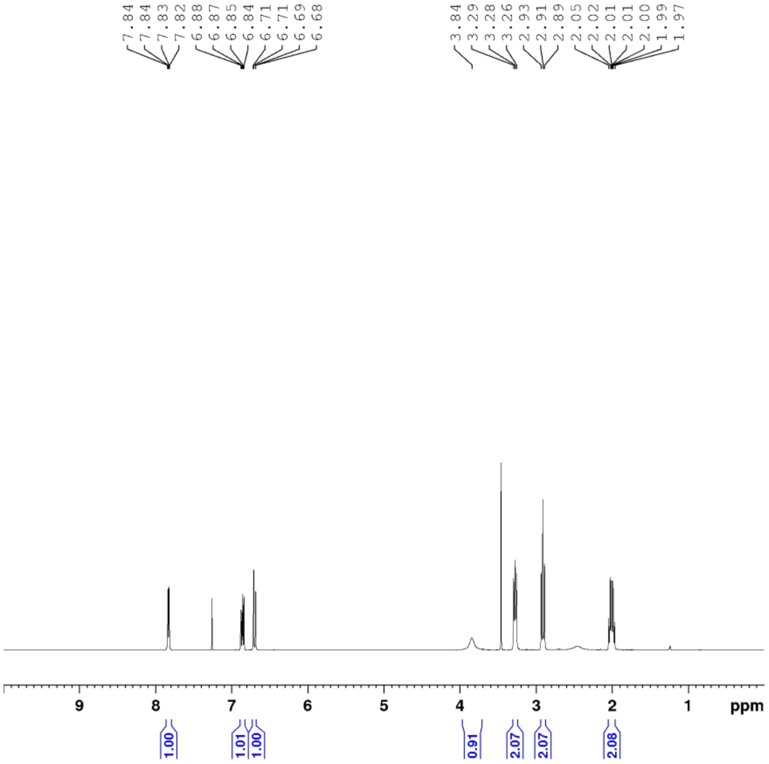

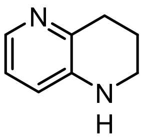

2ab

Figure S85. ^{1}H NMR spectrum of **2ab** in $CDCl_3$.

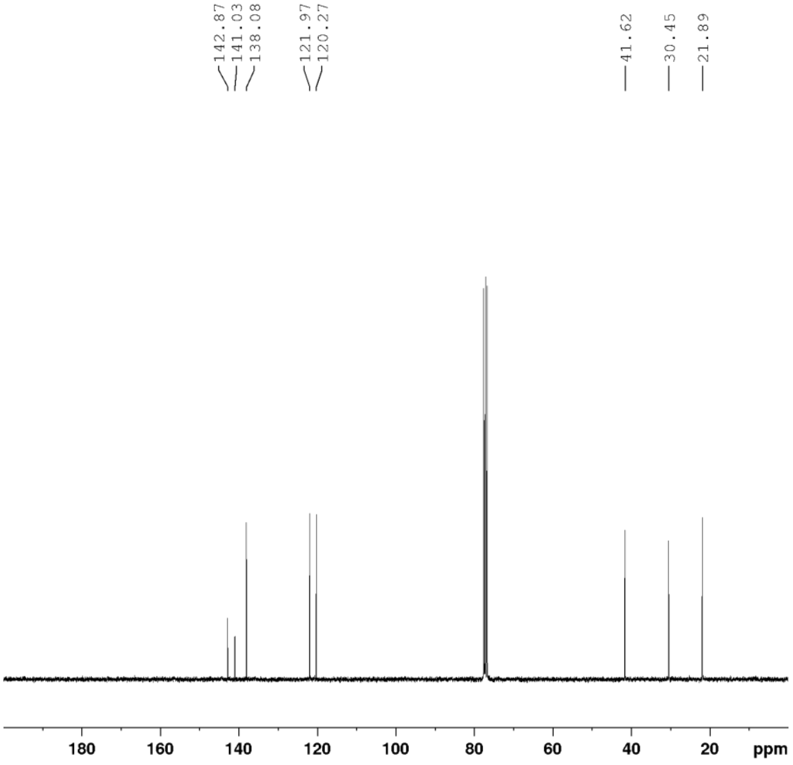

Figure S86. $^{13}C\{^{1}H\}$ NMR spectrum of **2ab** in $CDCl_3$.

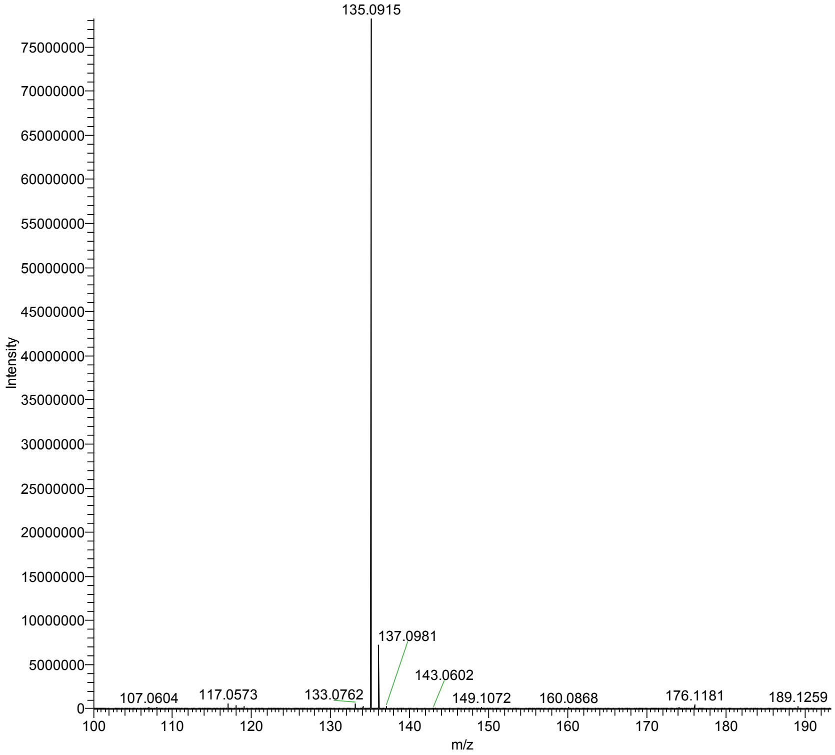

Figure S87. HRMS of **2ab**.

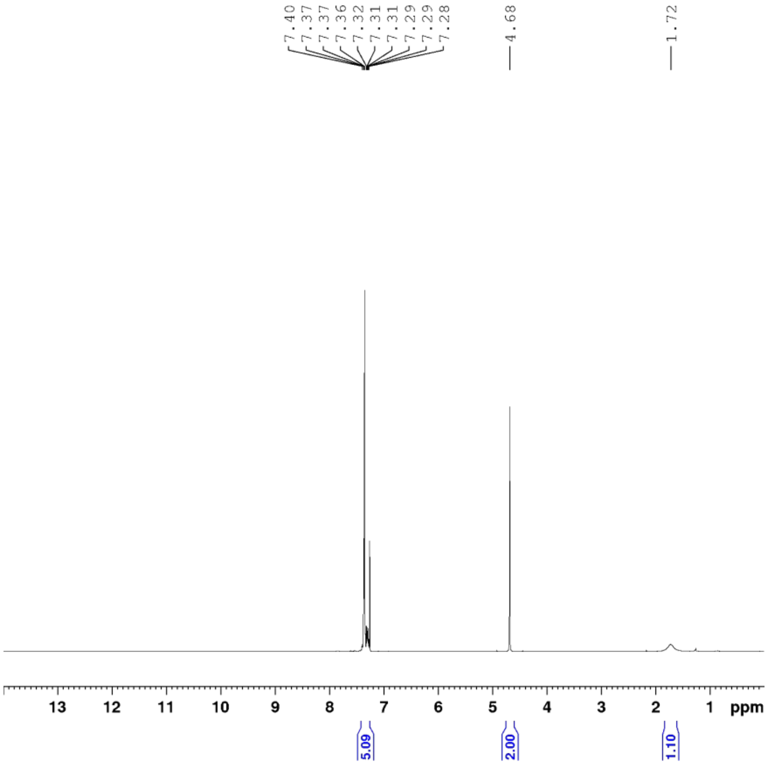

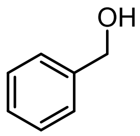

4a

Figure S88. ^{1}H NMR spectrum of **4a** in $CDCl_3$.

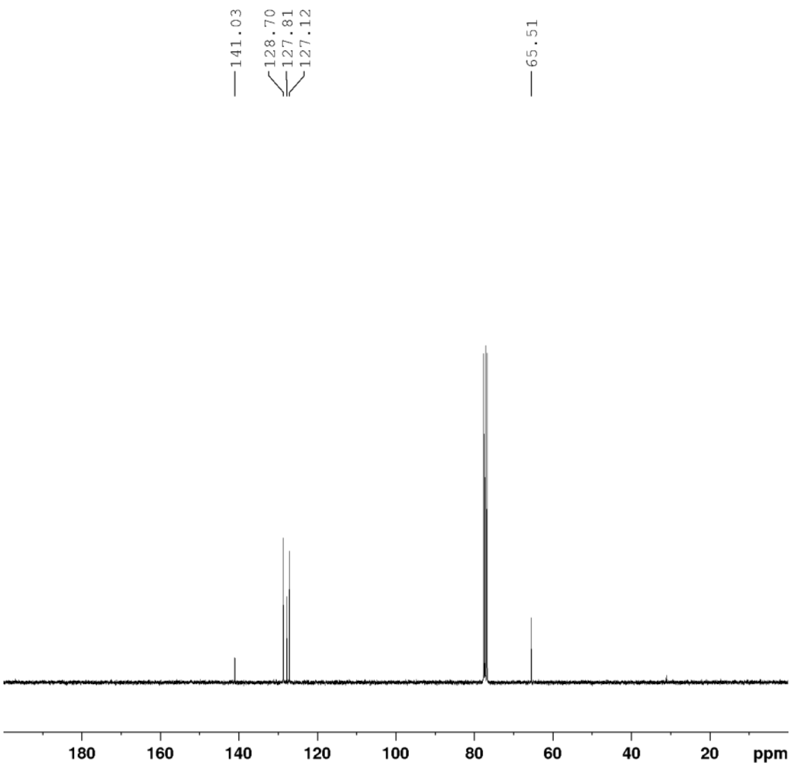

Figure S89. $^{13}C\{^1H\}$ NMR spectrum of **4a** in $CDCl_3$.

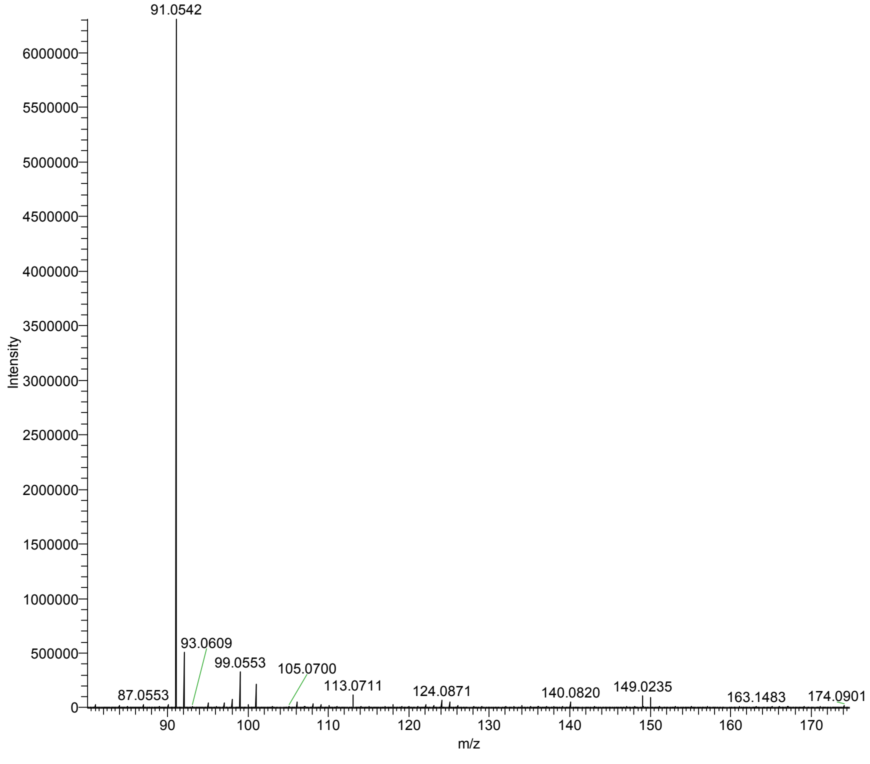

Figure S90. HRMS of **4a** ($MH^+ - H_2O$).

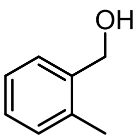

4b

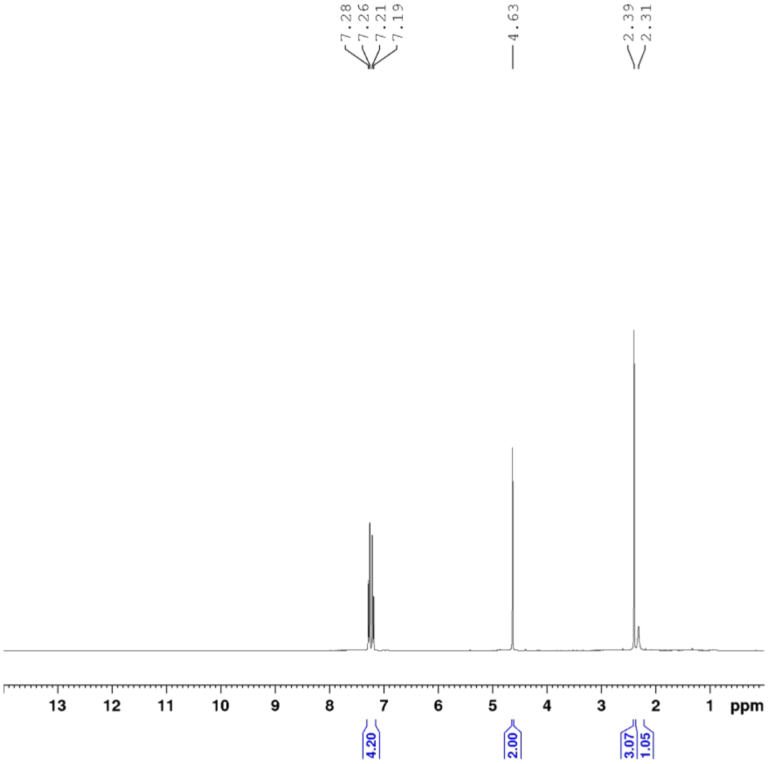

Figure S91. ^{1}H NMR spectrum of **4b** in $CDCl_3$.

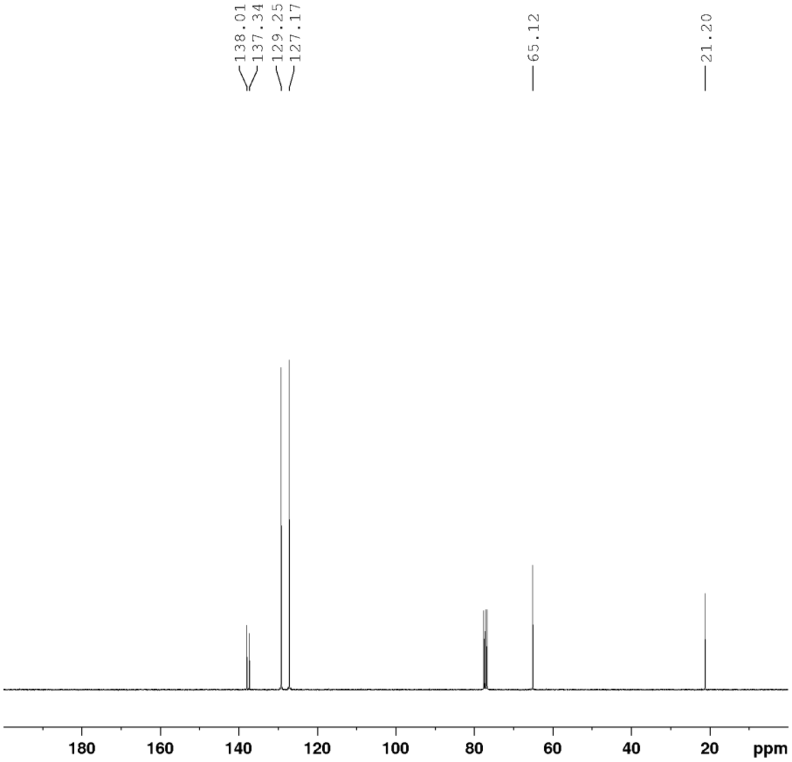

Figure S92. $^{13}C\{^{1}H\}$ NMR spectrum of **4b** in $CDCl_3$.

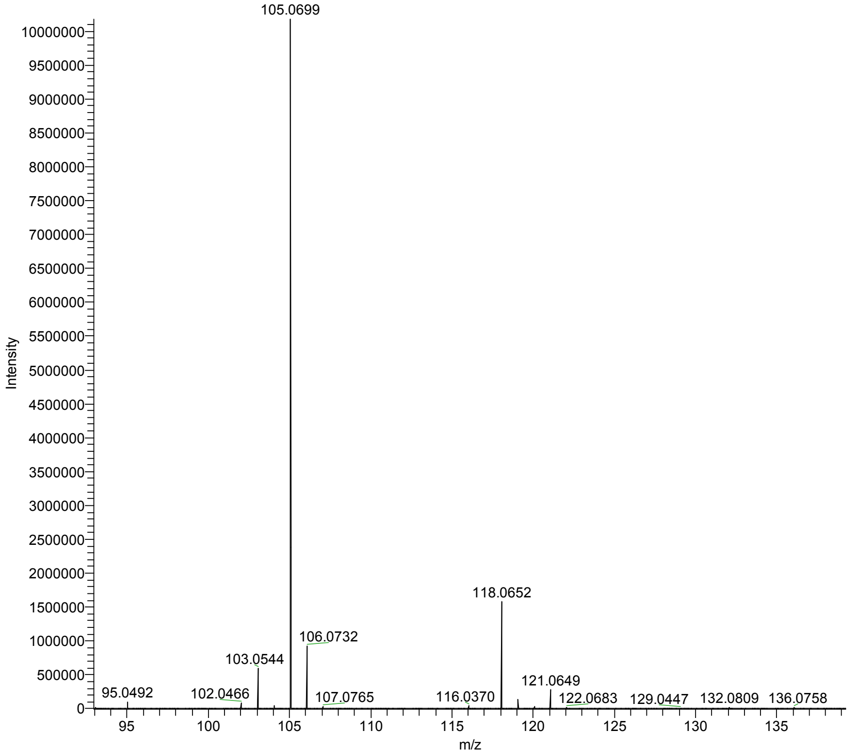

Figure S93. HRMS of **4b** ($MH^+ - H_2O$).

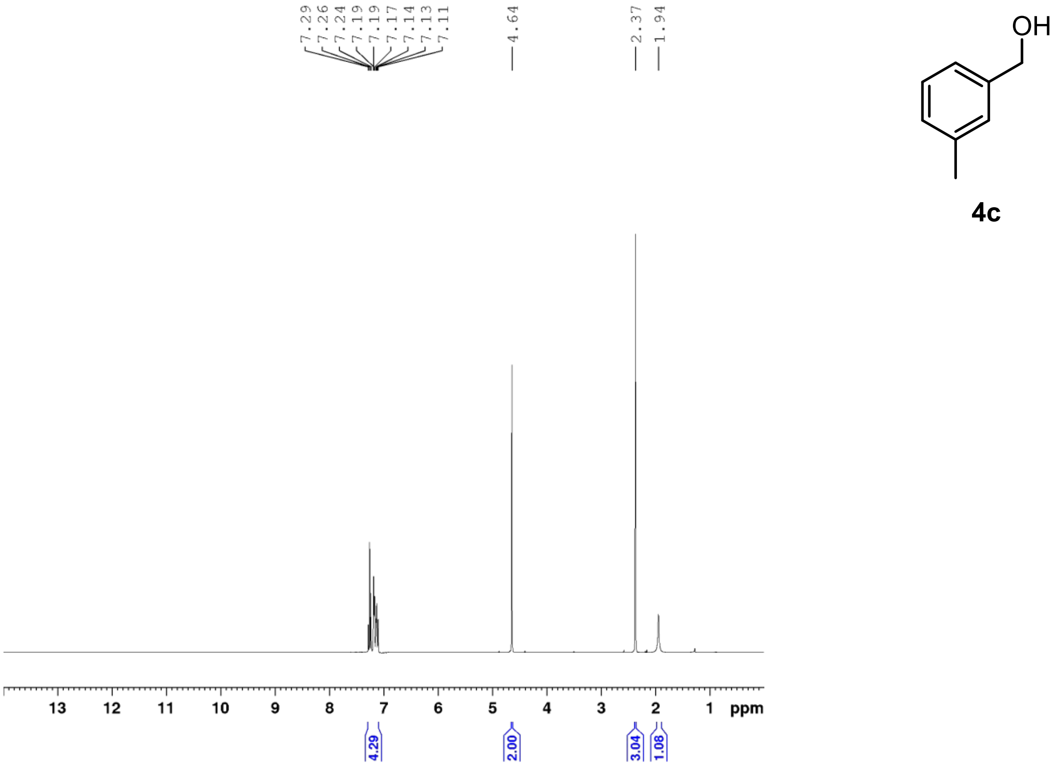

Figure S94. ^1H NMR spectrum of **4c** in $CDCl_3$.

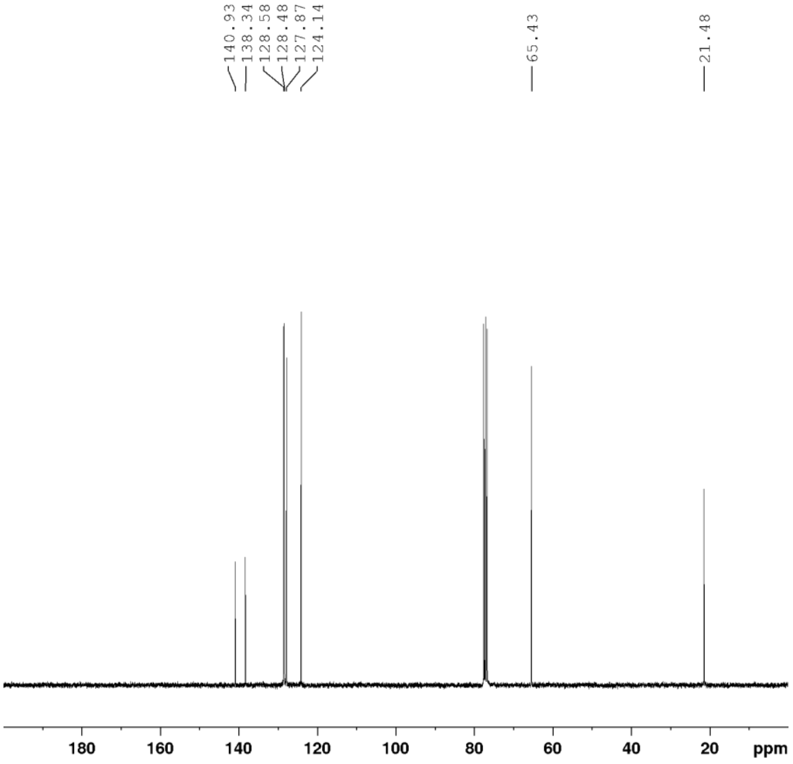

Figure S95. $^{13}C\{^{1}H\}$ NMR spectrum of **4c** in $CDCl_3$.

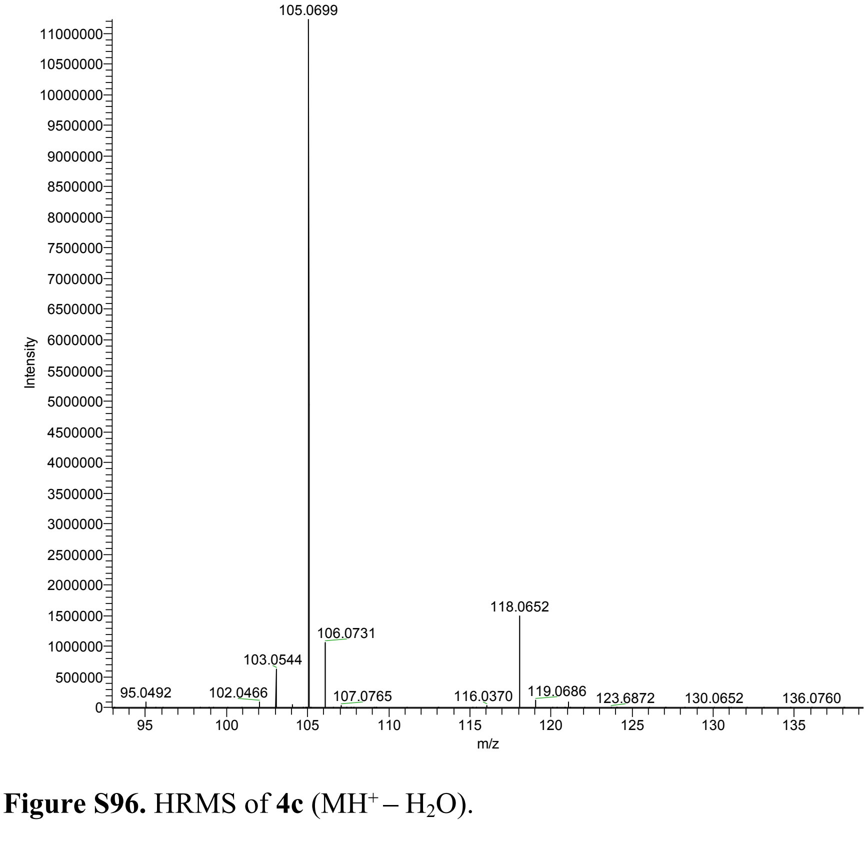

Figure S96. HRMS of **4c** ($MH^+ - H_2O$).

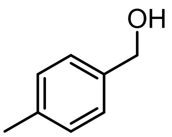

4d

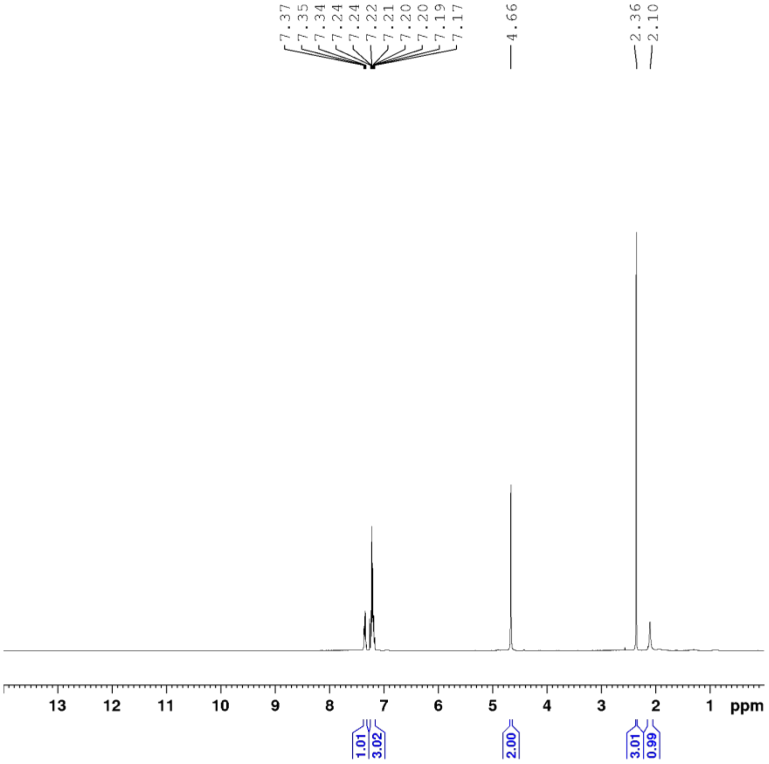

Figure S97. ^{1}H NMR spectrum of **4d** in $CDCl_3$.

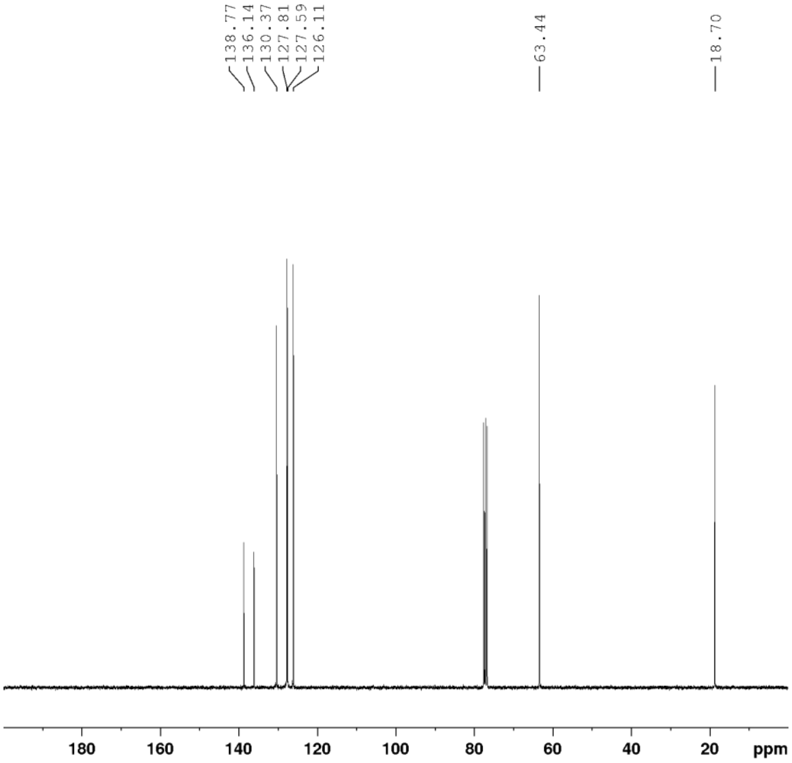

Figure S98. $^{13}C\{^{1}H\}$ NMR spectrum of **4d** in $CDCl_3$.

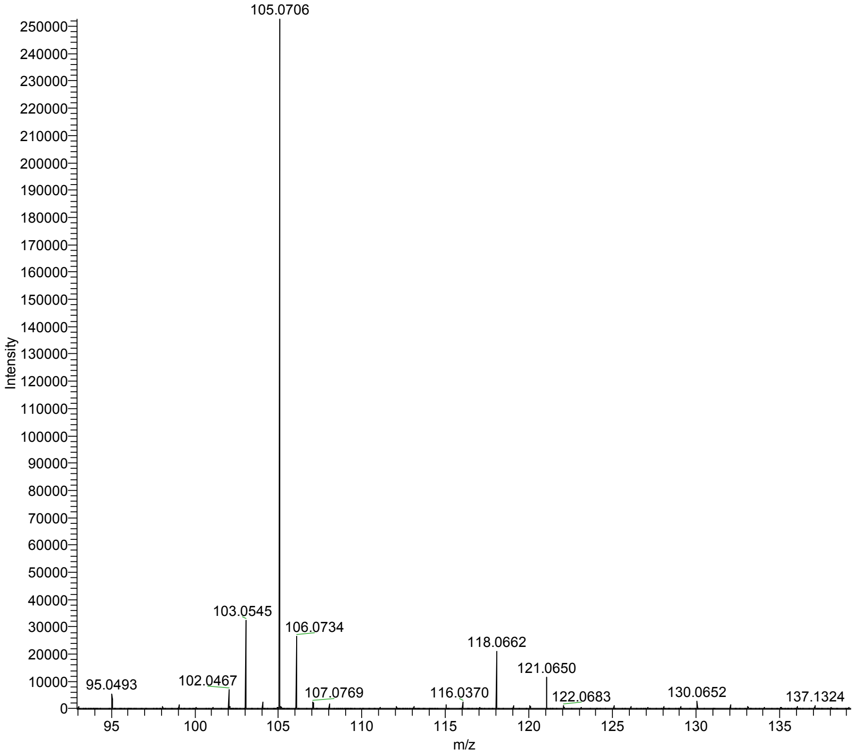

Figure S99. HRMS of **4d** ($MH^+ - H_2O$).

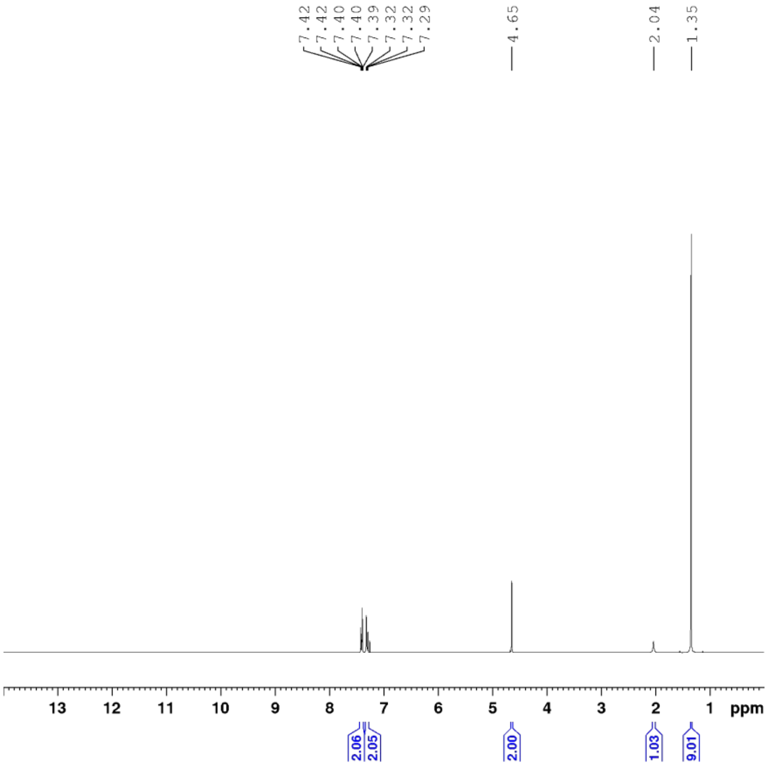

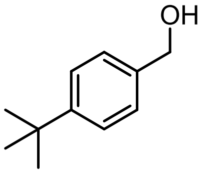

4e

Figure S100. ^1H NMR spectrum of **4e** in $CDCl_3$.

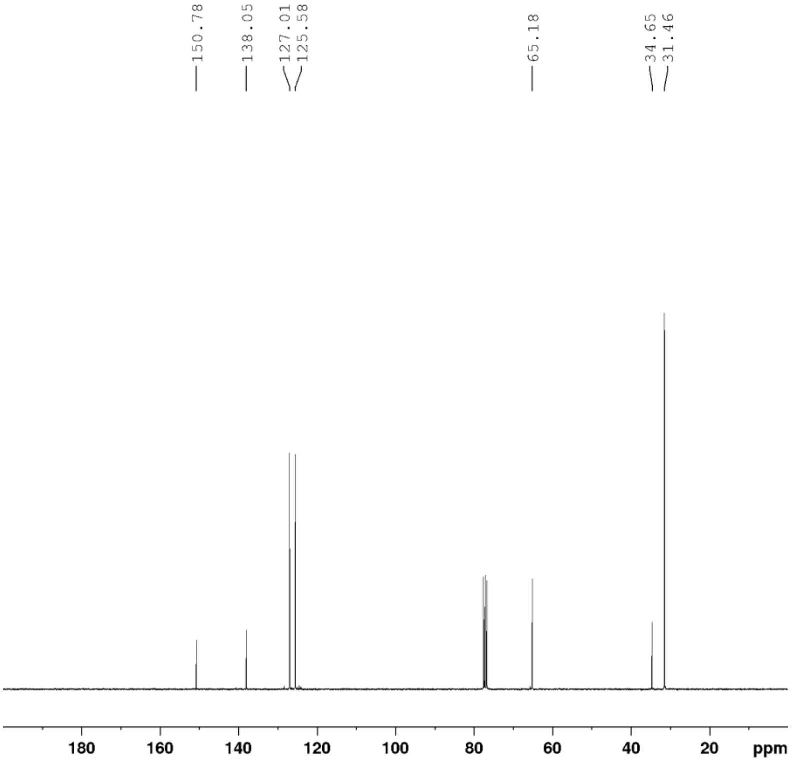

Figure S101. $^{13}C\{^{1}H\}$ NMR spectrum of **4e** in $CDCl_3$.

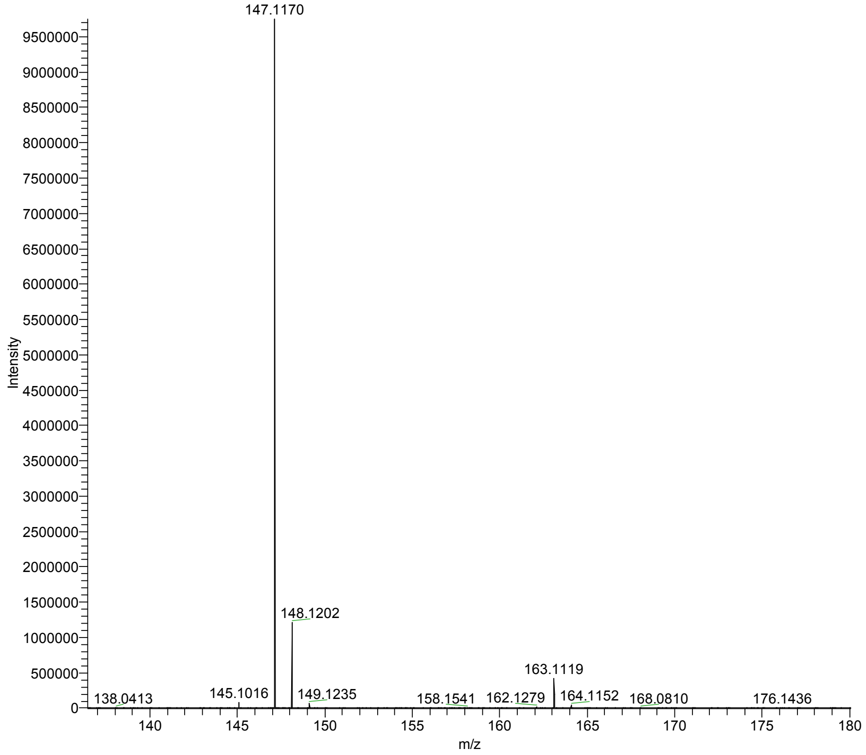

Figure S102. HRMS of **4e** ($MH^{+} – H_2O$).

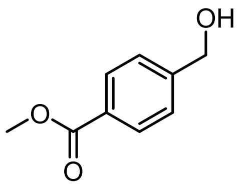

4f

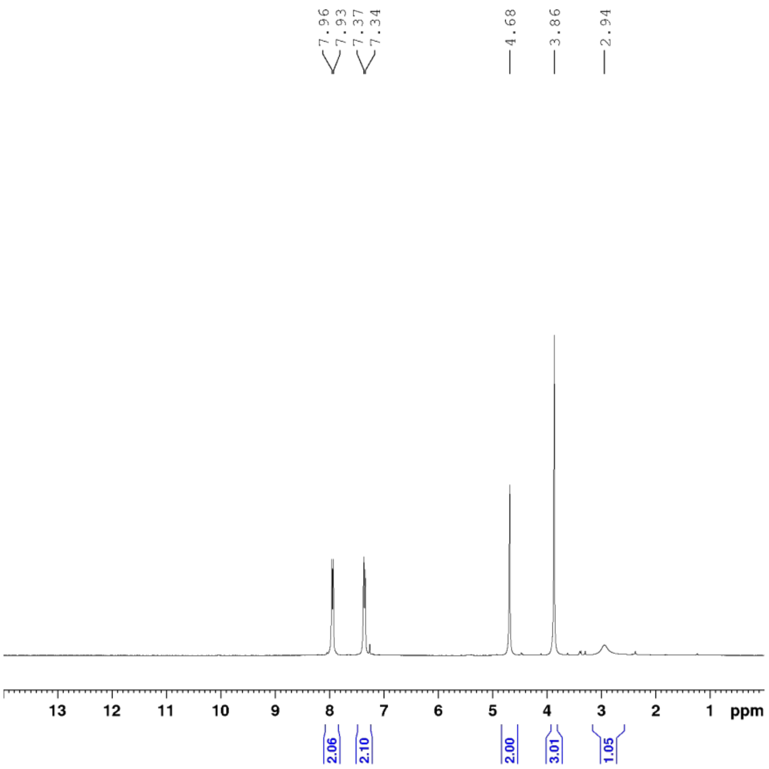

Figure S103. ^{1}H NMR spectrum of **4f** in $CDCl_3$.

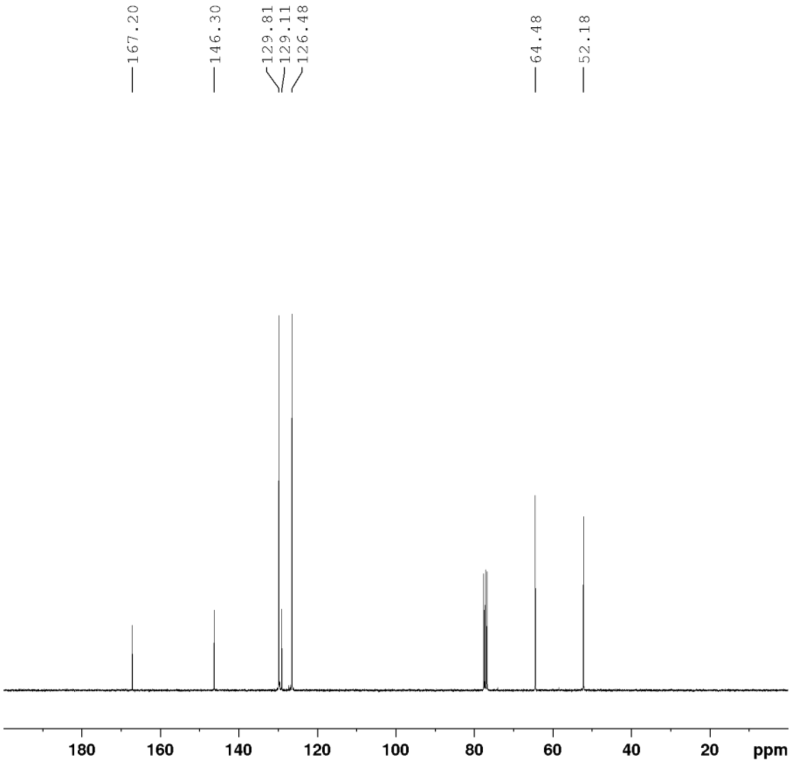

Figure S104. $^{13}C\{^{1}H\}$ NMR spectrum of **4f** in $CDCl_3$.

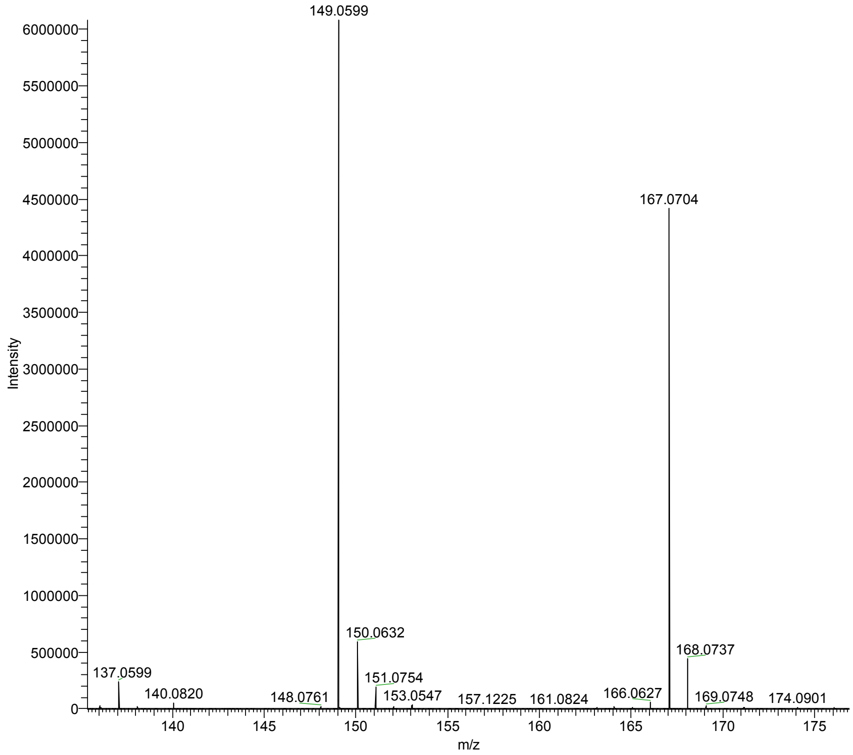

Figure S105. HRMS of **4f**.

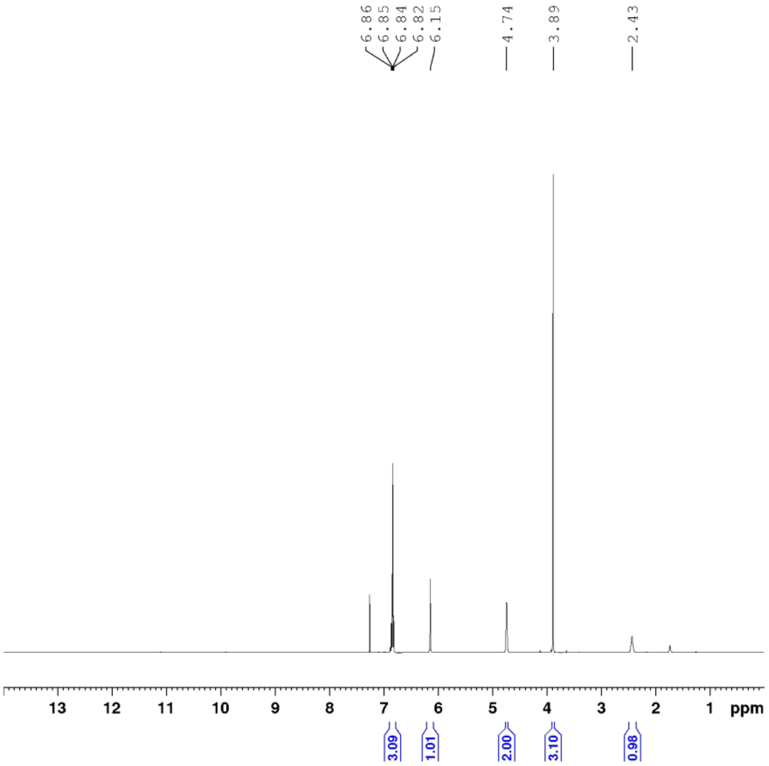

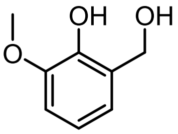

4g

Figure S106. ^{1}H NMR spectrum of **4g** in $CDCl_3$.

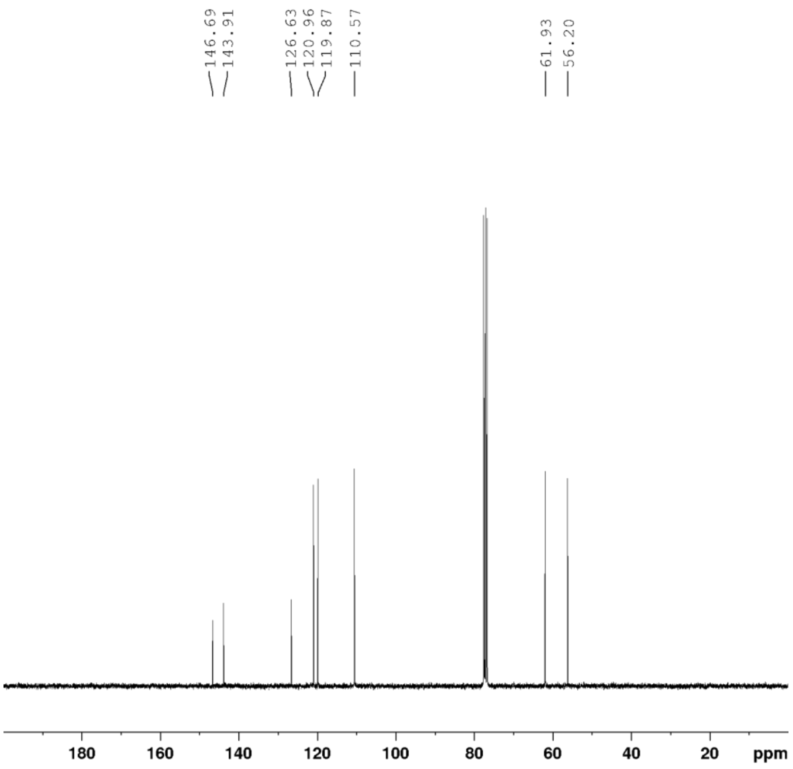

Figure S107. $^{13}C\{^1H\}$ NMR spectrum of **4g** in $CDCl_3$.

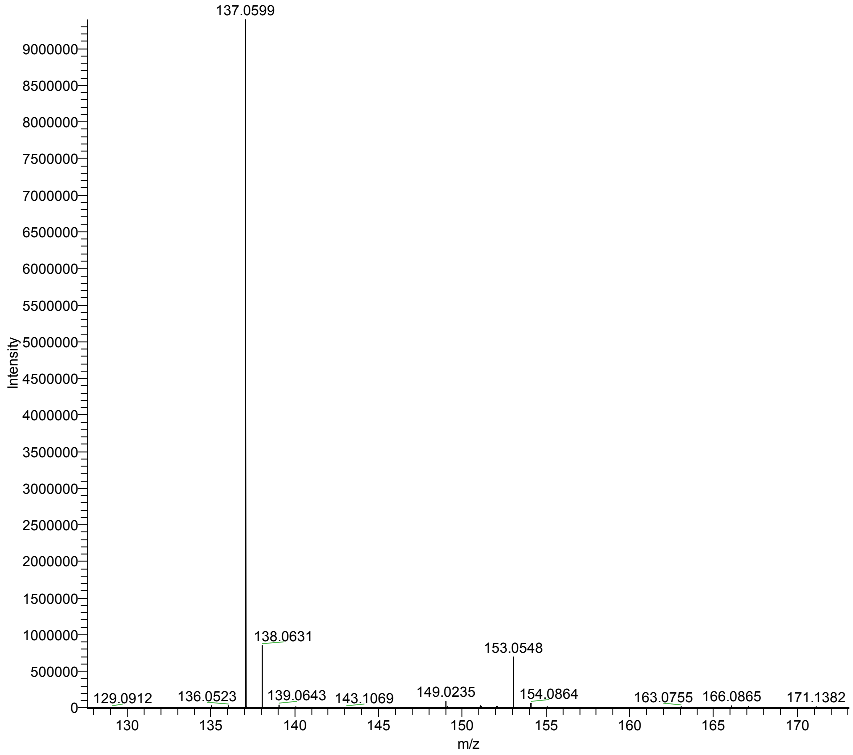

Figure S108. HRMS of **4g** ($MH^+ - H_2O$).

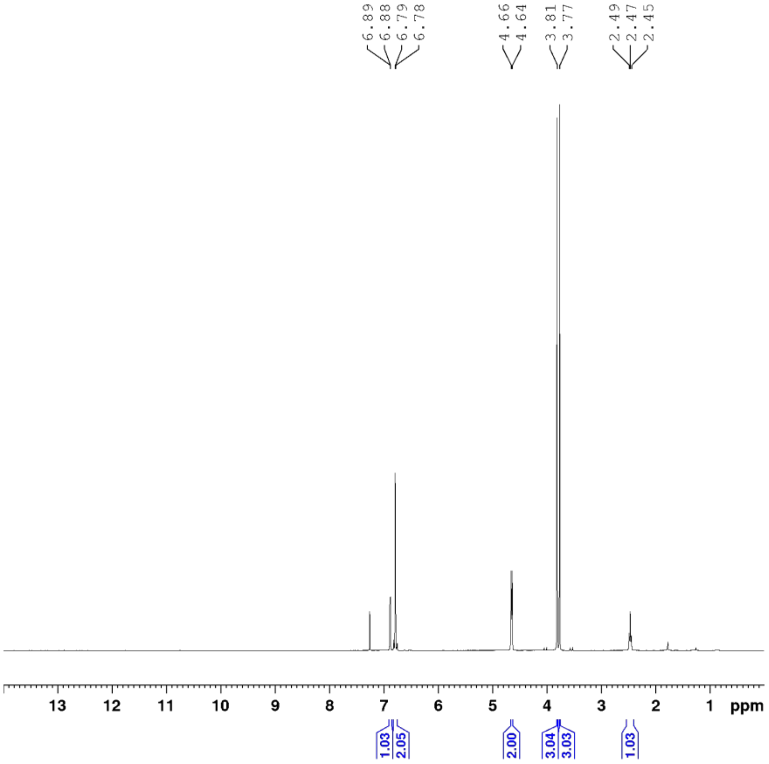

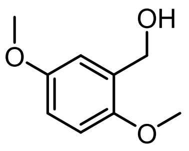

4h

Figure S109. ^{1}H NMR spectrum of **4h** in $CDCl_3$.

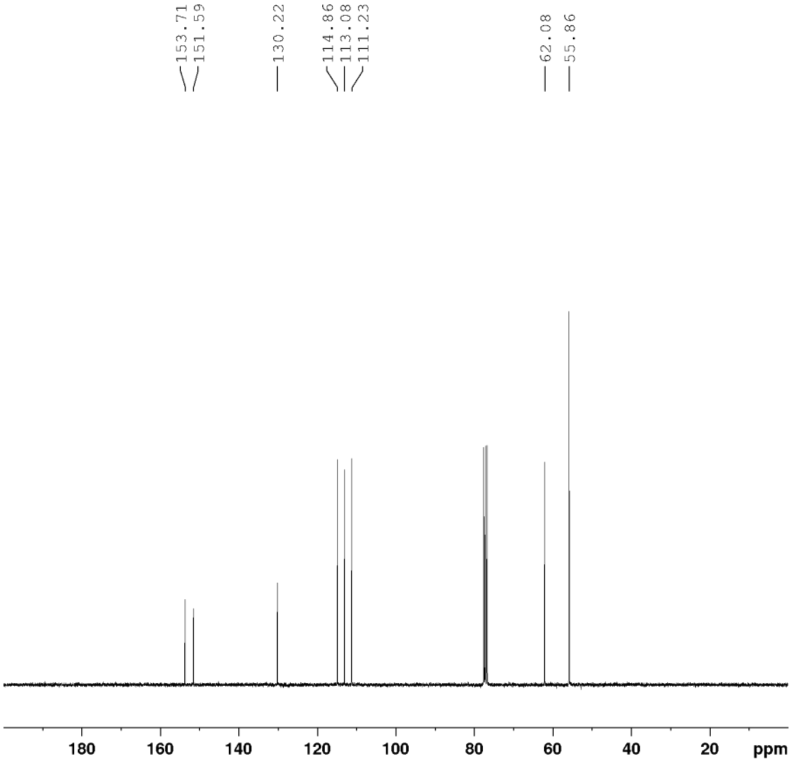

Figure S110. $^{13}C\{^{1}H\}$ NMR spectrum of **4h** in $CDCl_3$.

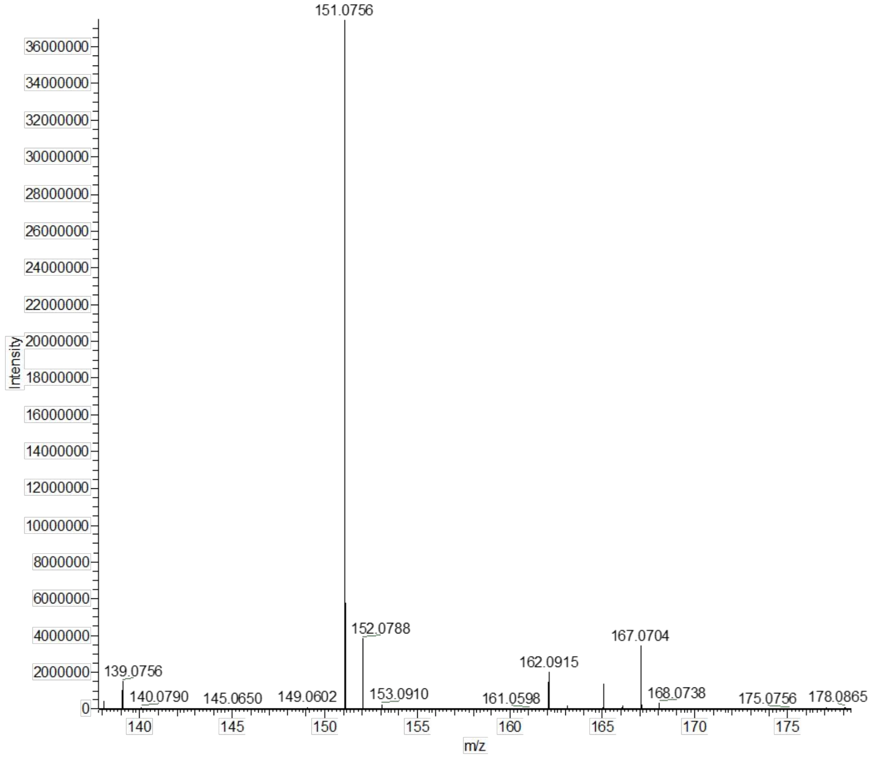

Figure S111. HRMS of **4h** ($MH^+ - H_2O$).

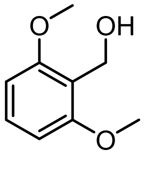

4i

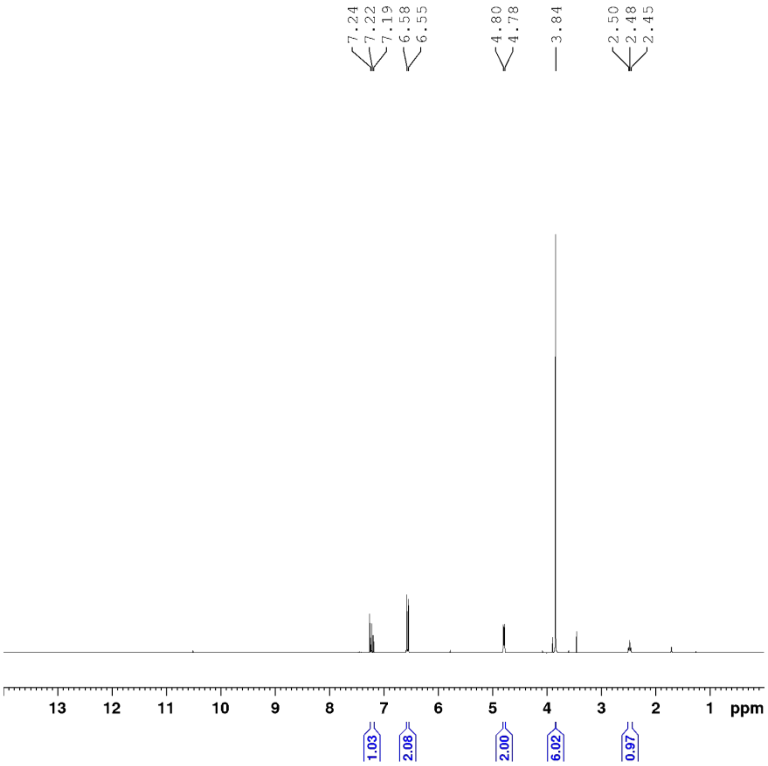

Figure S112. ^{1}H NMR spectrum of **4i** in $CDCl_3$.

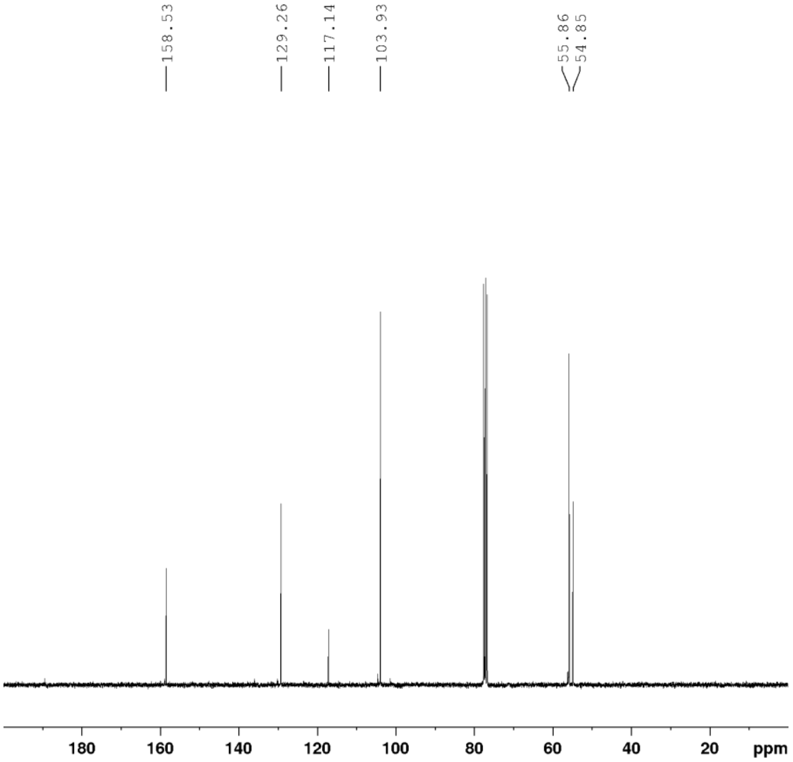

Figure S113. $^{13}C\{^1H\}$ NMR spectrum of **4i** in $CDCl_3$.

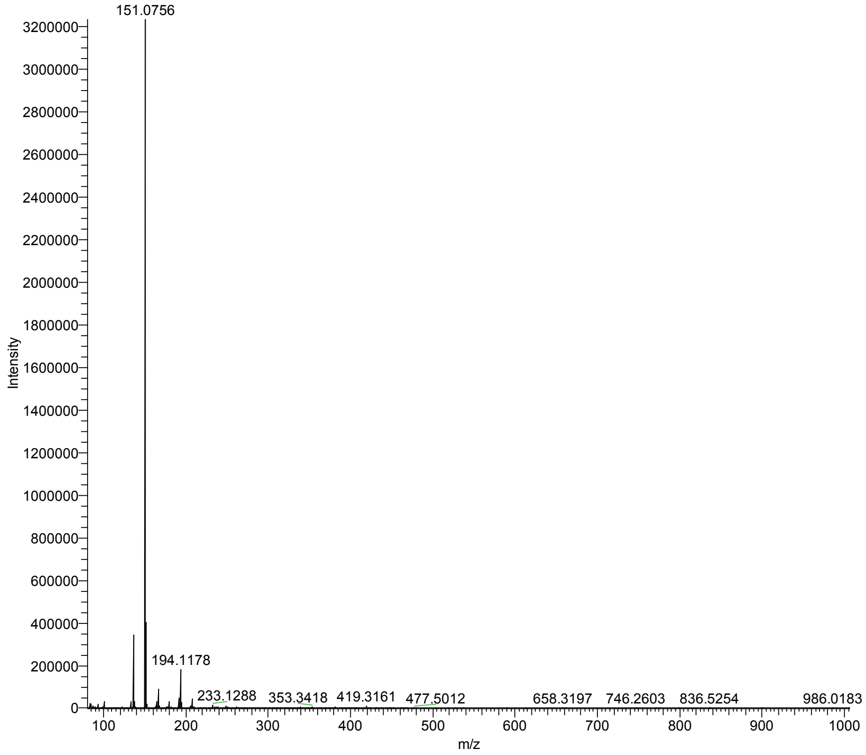

Figure S114. HRMS of **4i** ($MH^+ - H_2O$).

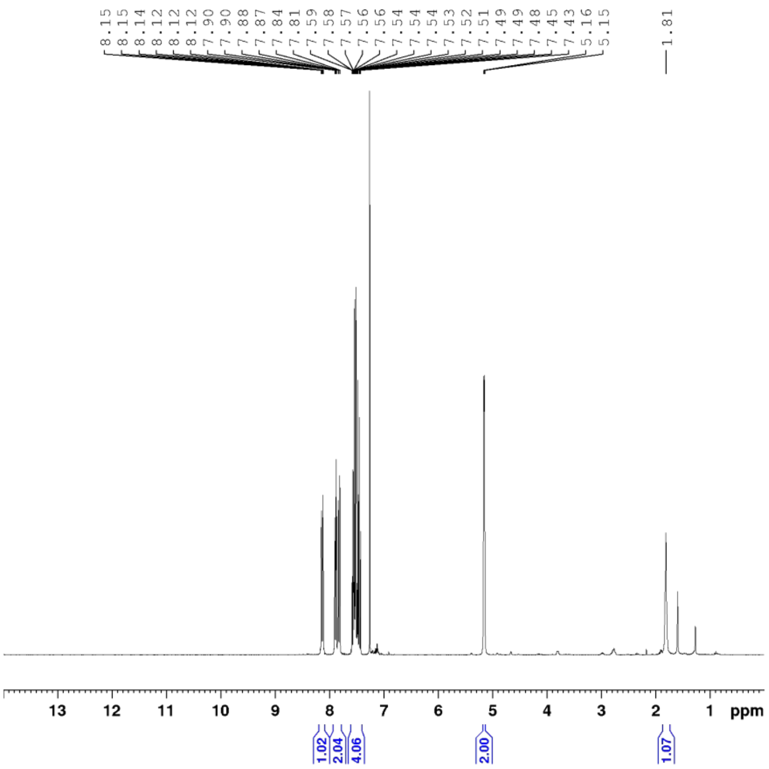

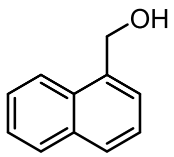

4j

Figure S115. ^{1}H NMR spectrum of **4j** in $CDCl_3$.

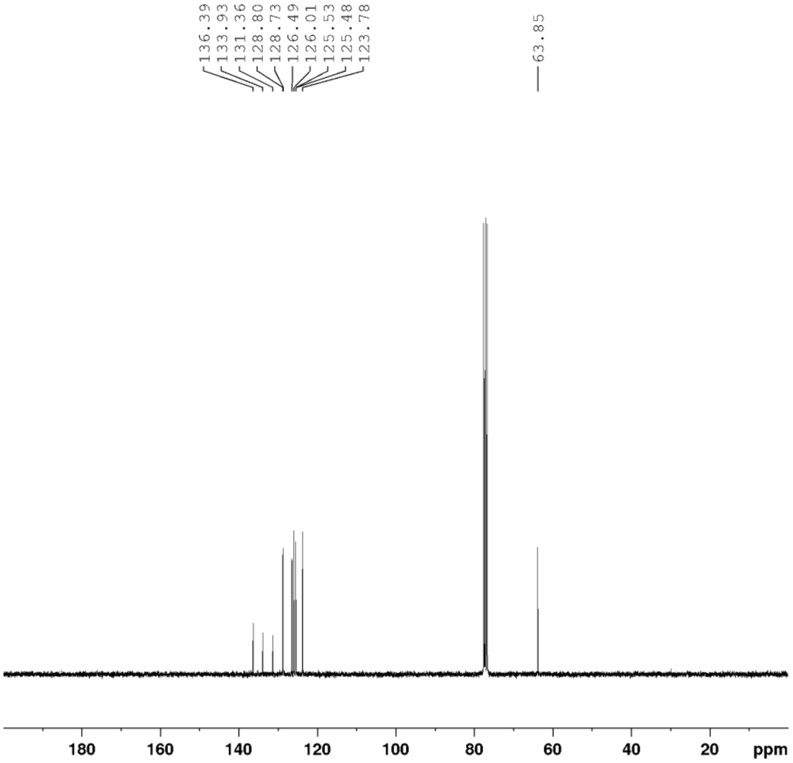

Figure S116. $^{13}C\{^{1}H\}$ NMR spectrum of **4j** in $CDCl_3$.

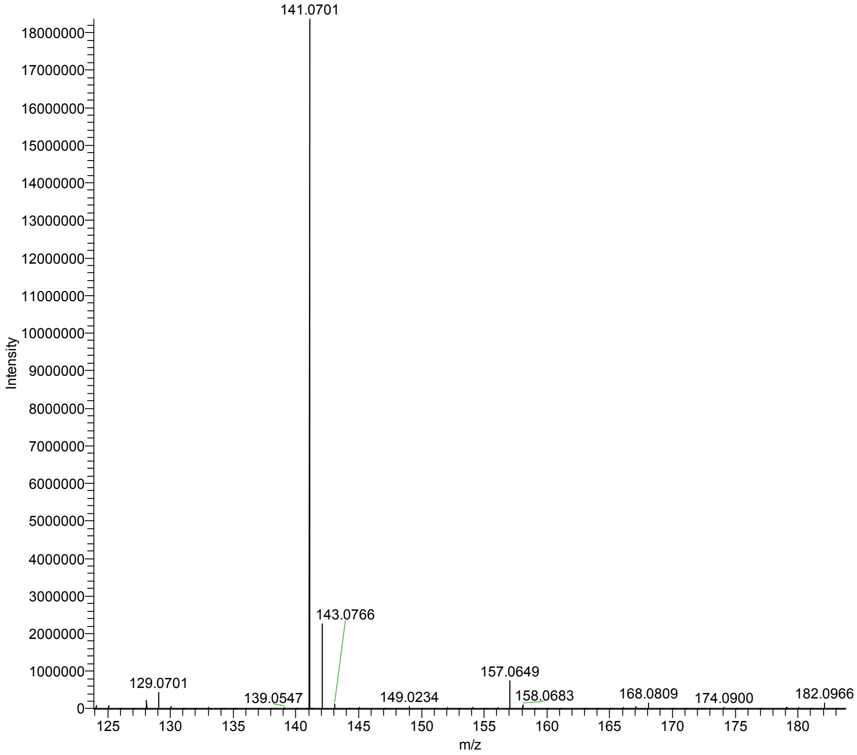

Figure S117. HRMS of **4j** ($MH^+ - H_2O$).

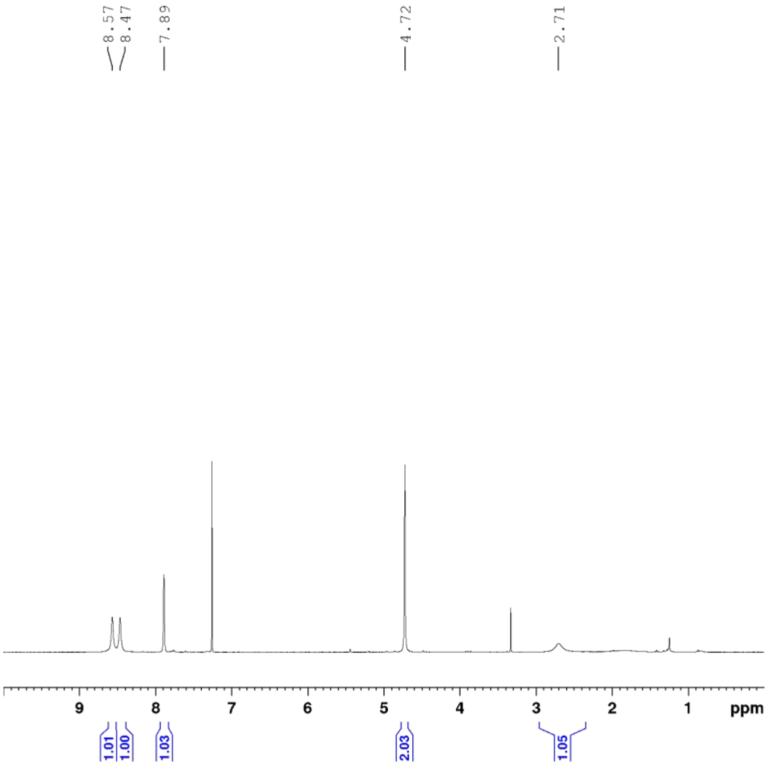

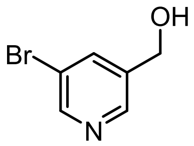

4k

Figure S118. ^1H NMR spectrum of **4k** in $CDCl_3$.

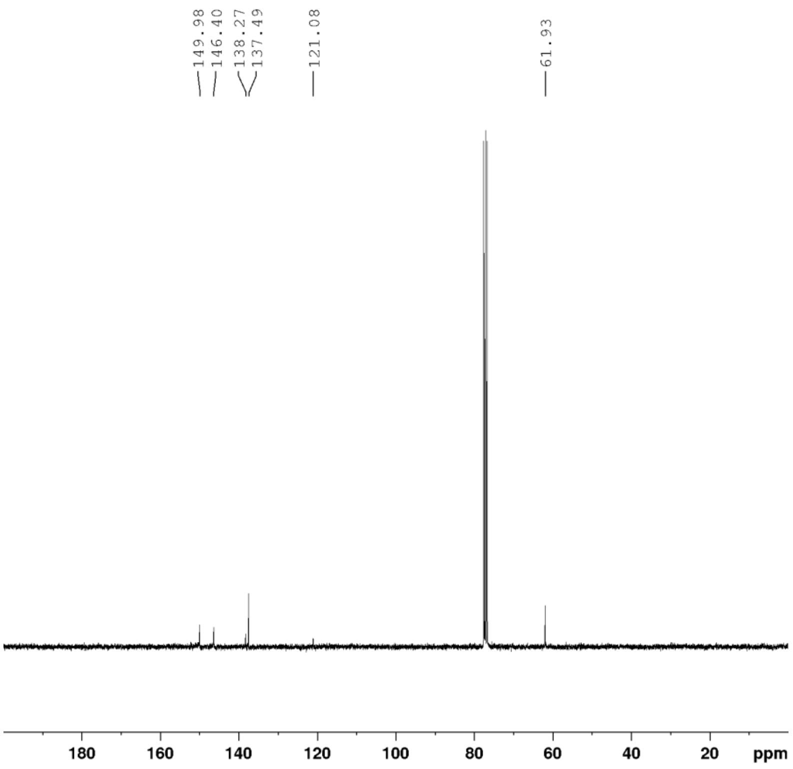

Figure S119. $^{13}C\{^{1}H\}$ NMR spectrum of **4k** in $CDCl_3$.

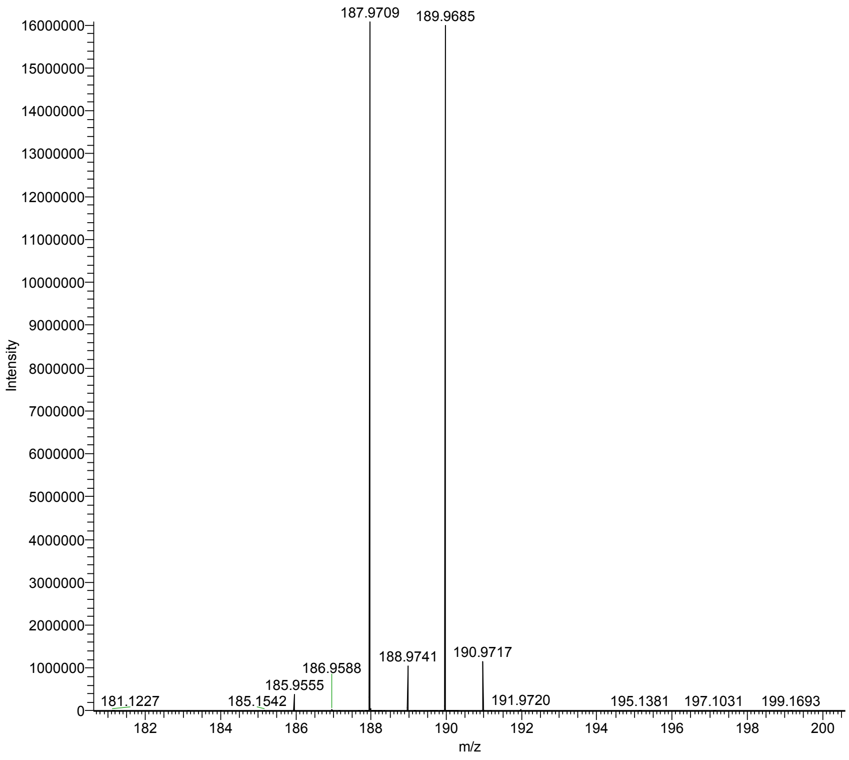

Figure S120. HRMS of **4k**.

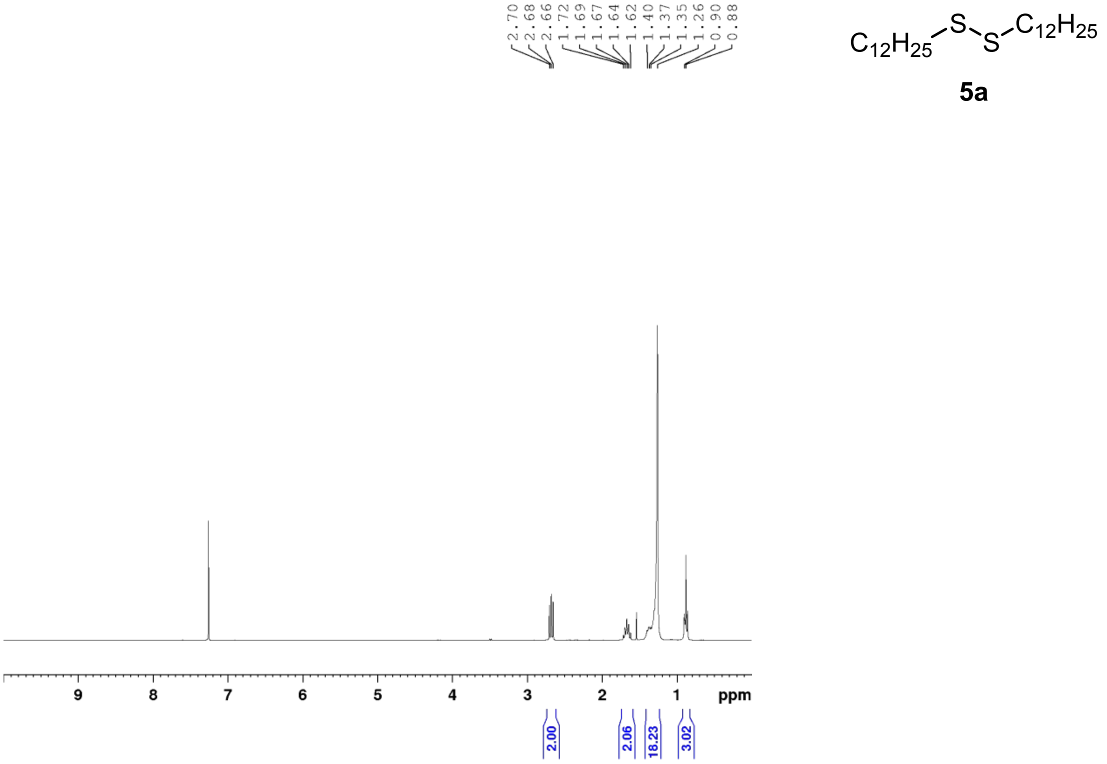

Figure S121. ^{1}H NMR spectrum of **5a**in $CDCl_3$.

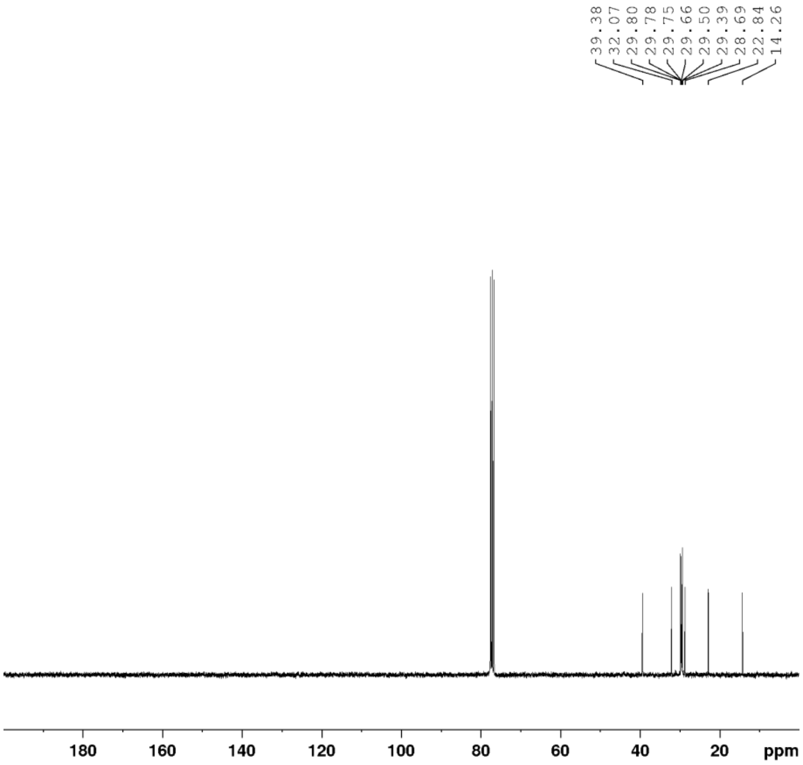

Figure S122. $^{13}C\{^1H\}$ NMR spectrum of **5a** in $CDCl_3$.

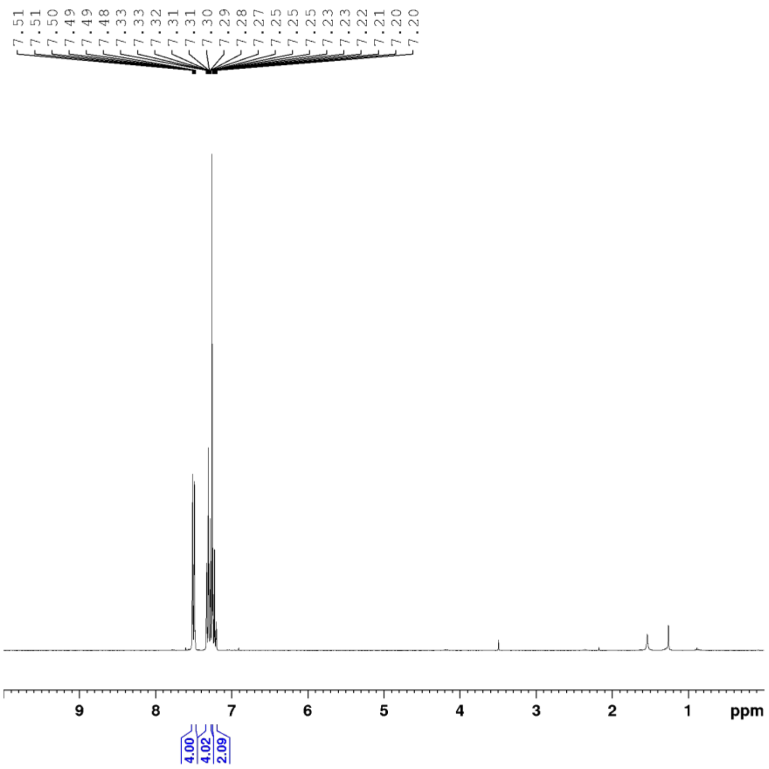

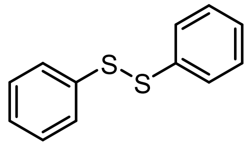

5b

Figure S123. ^{1}H NMR spectrum of **5b** in $CDCl_3$.

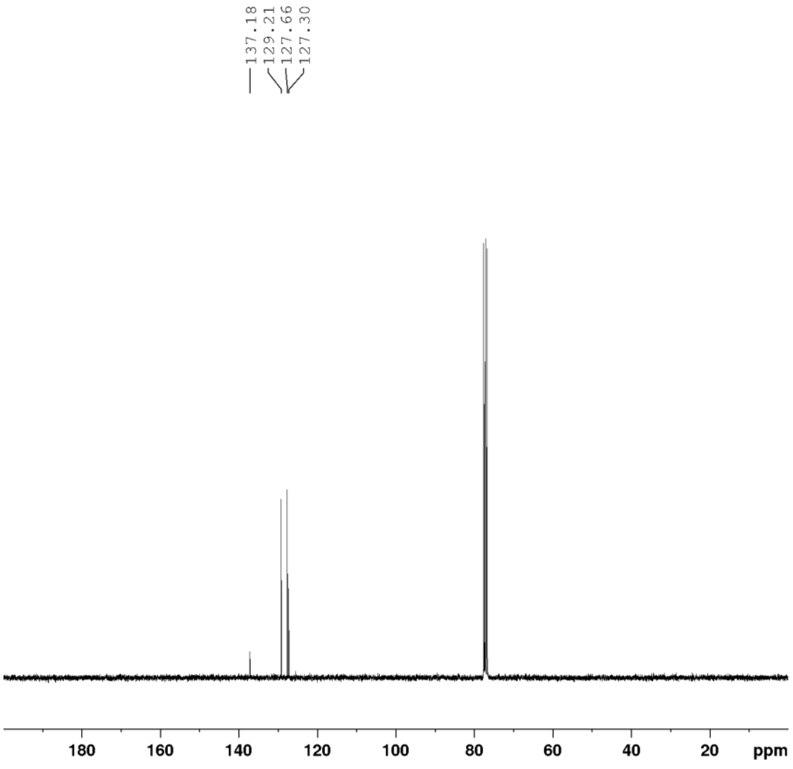

Figure S124. $^{13}C\{^{1}H\}$ NMR spectrum of **5b** in $CDCl_3$.

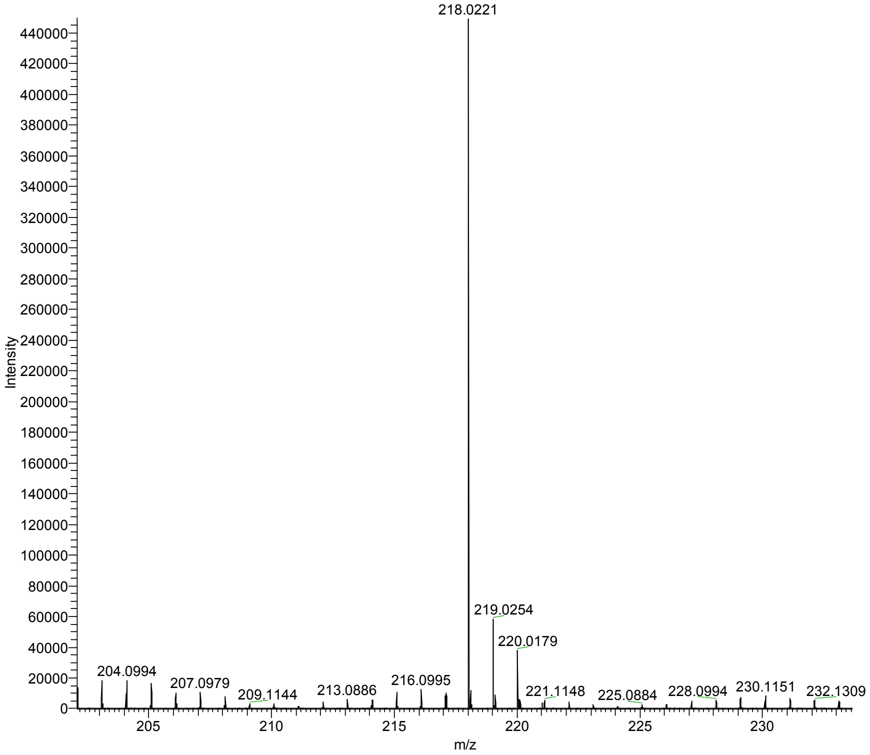

Figure S125. HRMS of **5b**.

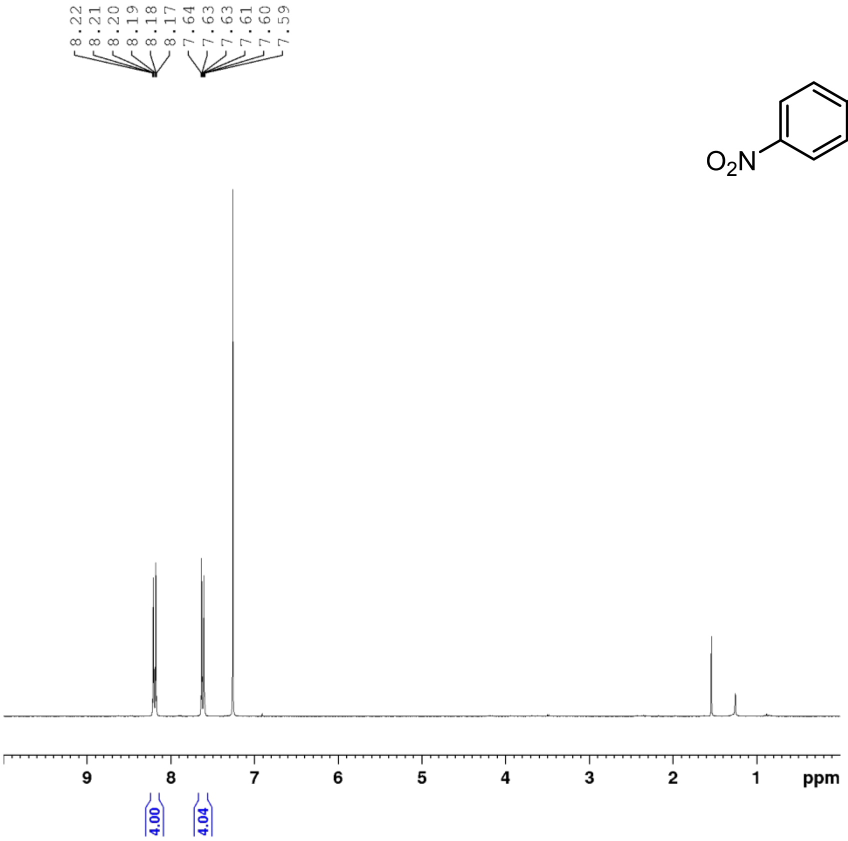

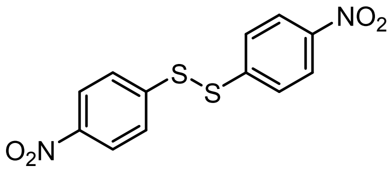

5c

Figure S126. ^{1}H NMR spectrum of **5c** in $CDCl_3$.

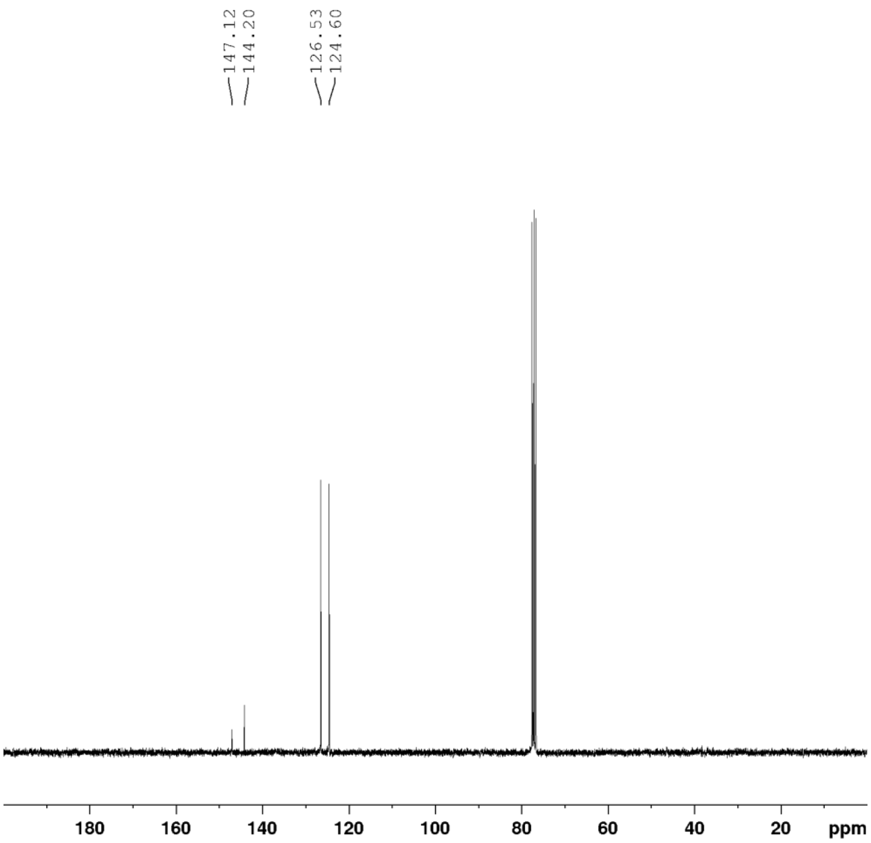

Figure S127. $^{13}C\{^1H\}$ NMR spectrum of **5c** in $CDCl_3$.

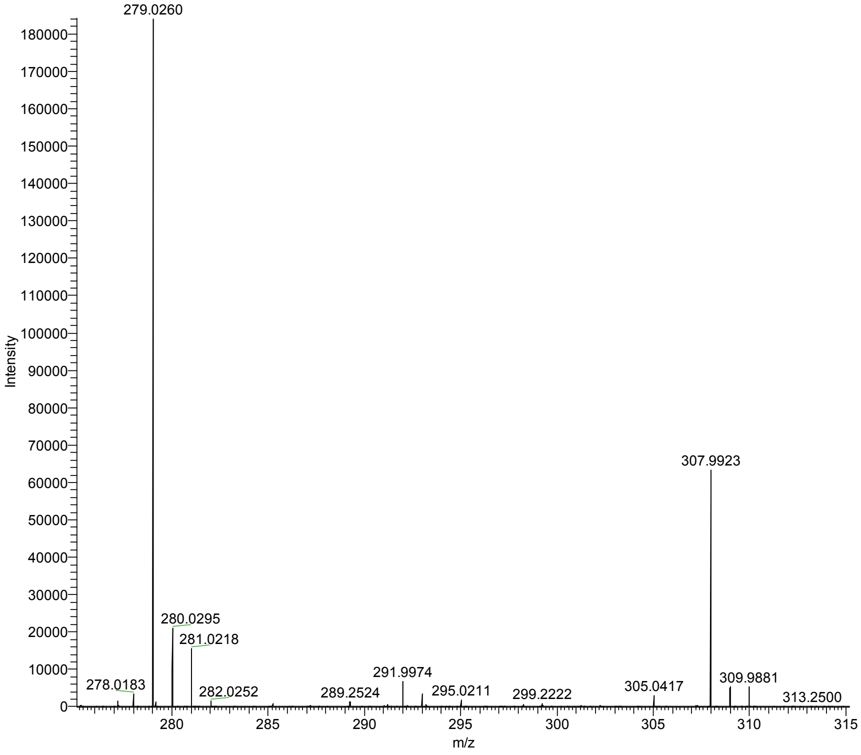

Figure S128. HRMS of **5c**.

12. References

(1) Wanjun, T.; Donghua, C.; Mechanism of Thermal Decomposition of Cobalt Acetate Tetrahydrate. *Chem. Pap.* **2007**, *61* (4), 329-332.

(2) Stoll, S.; Schweiger, A. EasySpin, a Comprehensive Software Package for Spectral Simulation and Analysis in EPR. *J. Magn. Reson.* **2006**, *178* (1), 42–55.

(3) Bolzacchini, E.; Chiavetto, L. B.; Canevali, C.; Morazzoni, F.; Orlandi, M.; Rindone, B.; Oxidation of Propenoidic Phenols Catalysed by *N*,*N′*-Ethylenebis(salicylideneiminato) Cobalt (II) [Cosalen]: Reactivity and Spectroscopic Studies *J. Mol. Catal. A: Chem.* **1996**, *112* (3), 347–351.

(4) Hamlin, J. E.; Hirai, K.; Millan, A.; Maitlis, P. M. A Simple Practical Test for Distinguishing a Heterogeneous Component in a Homogeneously Catalyzed Reaction. *J. Mol. Catal.* **1980**, *7* (4), 543–544.
